# The critical balance between dopamine D2 receptor and RGS for the sensitive detection of a transient decay in dopamine signal

**DOI:** 10.1371/journal.pcbi.1009364

**Published:** 2021-09-30

**Authors:** Hidetoshi Urakubo, Sho Yagishita, Haruo Kasai, Yoshiyuki Kubota, Shin Ishii

**Affiliations:** 1 Integrated Systems Biology Laboratory, Department of Systems Science, Graduate School of Informatics, Kyoto University, Kyoto, Japan; 2 Section of Electron Microscopy, National Institute for Physiological Sciences, Okazaki, Aichi, Japan; 3 Laboratory of Structural Physiology, Center for Disease Biology and Integrative Medicine, Faculty of Medicine, University of Tokyo, Bunkyo-ku, Tokyo, Japan; 4 International Research Center for Neurointelligence (WPI-IRCN), University of Tokyo Institutes for Advanced Study (UTIAS), Tokyo, Japan; 5 Department of Physiological Sciences, The Graduate University for Advanced Studies (SOKENDAI), Okazaki, Aichi, Japan; Instytut Biologii Doswiadczalnej im M Nenckiego Polskiej Akademii Nauk, POLAND

## Abstract

In behavioral learning, reward-related events are encoded into phasic dopamine (DA) signals in the brain. In particular, unexpected reward omission leads to a phasic decrease in DA (DA dip) in the striatum, which triggers long-term potentiation (LTP) in DA D2 receptor (D2R)-expressing spiny-projection neurons (D2 SPNs). While this LTP is required for reward discrimination, it is unclear how such a short DA-dip signal (0.5–2 s) is transferred through intracellular signaling to the coincidence detector, adenylate cyclase (AC). In the present study, we built a computational model of D2 signaling to determine conditions for the DA-dip detection. The DA dip can be detected only if the basal DA signal sufficiently inhibits AC, and the DA-dip signal sufficiently disinhibits AC. We found that those two requirements were simultaneously satisfied only if two key molecules, D2R and regulators of G protein signaling (RGS) were balanced within a certain range; this balance has indeed been observed in experimental studies. We also found that high level of RGS was required for the detection of a 0.5-s short DA dip, and the analytical solutions for these requirements confirmed their universality. The imbalance between D2R and RGS is associated with schizophrenia and DYT1 dystonia, both of which are accompanied by abnormal striatal LTP. Our simulations suggest that D2 SPNs in patients with schizophrenia and DYT1 dystonia cannot detect short DA dips. We finally discussed that such psychiatric and movement disorders can be understood in terms of the imbalance between D2R and RGS.

## Author summary

In our brain, learning and memory are strongly modulated by dopamine (DA) signals. Even a short absence of DA (0.5–2 s), called “DA dip,” triggers long-term memory formation, the underlying processes of which are hitherto largely unknown. Here, we examined how the DA dips are processed through a biochemical signaling network to generate long-term memory. Computer simulation and theoretical analyses showed that the DA-dip signal is processed only if the levels of two key molecules, DA D2 receptor (D2R) and regulators of G protein signaling (RGS), are both delicately balanced. This balance seems to be achieved in the healthy brain, whereas imbalance between D2R and RGS levels appear in patients with schizophrenia and DYT1 dystonia, both of which may manifest abnormal long-term memory. The D2R–RGS imbalances hamper DA-dip detectability, and thus disturb long-term memory formation, which may result in the symptoms of schizophrenia and dystonia. The balance between D2R and RGS appear to be finely regulated in the healthy brain to underpin normal learning and memory.

## Introduction

In animals’ reward learning, phasic dopamine (DA) signal in the brain conveys important information, called reward prediction errors [1,2]. Unexpected reward causes a phasic increase in striatal DA level for 0.2–1 s (DA burst), whereas unexpected reward omission leads to a transient decrease in DA for 0.5–2 s (DA dip) [3,4]. Such phasic DA signals are decoded into striatal synaptic plasticity that refines animal behavior to obtain larger rewards [1,5–8]. In particular, we previously found that 0.5–2-s DA dips triggered long-term potentiation (LTP) in dopamine D2 receptor (D2R)-expressing spiny-projection neurons (D2 SPNs) of the striatum [1,9]. The LTP is induced only if the DA dip coincides with the presynaptic release of glutamate and postsynaptic burst firing of a D2 SPN (pre–post pairing), under the presence of adenosine [1,10]. The DA dip needs to occur together with pre–post pairing, and this LTP is required for reward-discrimination learning [1]. Intracellularly, the DA-dip signal leads to the deactivation of D2R and then the decrease in the GTP-bound form of inhibitory G protein (G_i_-GTP). The 0.5-s DA dip is similar to the timescale of the G-protein signaling (0.1–1 s) [11], raising a question about how the DA-dip signal is so reliably encoded into these signaling molecules. The same issue might be extended to psychiatric/movement disorders, because the alteration in D2R signaling and LTP in the striatum have been implicated [2,12,13].

We thus previously demonstrated using computational modeling, how such a rapid signal is encoded in the D2R signaling [10]. In the D2 LTP model, DA-bound D2R produces G_i_-GTP, and the G_i_-GTP inhibits adenylate cyclase (AC), in particular, AC type 1 (AC1) as in our case [1,5]. In contrast, neuronal firing and adenosine signals elevate the levels of Ca^2+^ and the GTP form of stimulatory G protein (G_olf_-GTP), respectively, both of which jointly activate AC1 [14,15]. Together, G_i_, G_olf_, and Ca^2+^ signals are integrated at AC1 that functions as a coincidence detector [10,15]. For this to occur, the DA-dip signal itself must be transmitted to the AC. The AC activity triggers the longer molecular dynamics, such as increase in cyclic adenosine monophosphate (cAMP), resulting in the occurrence of LTP [1,5].

The similar DA-dip detection seems to be realized in the other subtype of AC, AC type 5 (AC5), because AC5 is abundantly expressed in the striatum [16,17]. G_olf_-GTP alone sufficiently triggers the activity of AC5, while the AC5 activity is inhibited by G_i_ [18–20], and the DA-dip detection on AC5 should affect striatal synaptic plasticity [21] as well as the change of somatic neuronal excitability [22,23]. In particular, AC5 likely functions in the soma because somatic AC activity does not require the increase in Ca^2+^ level [5,19]. D2 signaling models have demonstrated that AC5 in D2 SPNs could detect short DA dips in conjunction with accompanying G_olf_ signals, referred to as the “tone sensing” mode [19,20,24]. In summary, in either case of the type 1 or 5, DA-dip signal must be transmitted to AC if it is processed through G_i_ signaling for the coincidence detection.

The models of D2 signaling have demonstrated DA-dip detection by AC; however, these models consist of complicated signaling involving many parameters [10,19,24]. It is thus difficult to capture what components of the model parameters, i.e., molecular concentrations and kinetics constants, are essential for the transmission of DA-dip signal. Regarding our D2 LTP model [10], some of the parameters were well constrained by experimental measurements (e.g. Ca^2+^ signal [25]), while others were determined only based on order estimations or ratio constraints, and the DA-dip detection may also depend on remaining unconstrained factors. One way to examine the parameter dependence is a sensitivity analysis [26], i.e., exhaustive quantification of the changes in model output associated with changes in parameter values. However, this analysis is essentially phenomenological, which cannot address underlying principles. If the concentration dependence of the D2 model is analytically solved, it would provide precise parameter dependence as well as the underlying mechanisms simultaneously.

Further, molecular concentrations in D2 SPNs are altered depending on the age and health condition. In particular, the expression levels of D2R and its counteracting molecule, regulators of G protein signaling type 9–2 (RGS9–2), are both increased with age [27,28]. In schizophrenia, a psychiatric disorder, D2R shows supersensitivity [29,30], whereas the expression level of RGS9–2 is decreased [31,32]. In DYT1 dystonia, a movement disorder, the balance between D2R and RGS9–2 is conversely biased toward RGS9–2 [28]. Those psychiatric/movement disorders are also known to show abnormal LTP in D2 SPNs [1,2,33]. Thus, the D2 LTP model should be examined under the various concentrations of D2R and RGS9–2. However, it is still poorly understood how the concentrations of D2R and RGS9–2 are related to LTP and brain function/dysfunction.

In the present study, we selectively targeted the D2R–G_i_–AC part (D2 model) from the previous D2-LTP model to clarify the requirements for the DA-dip detection. We first examined the concentration dependence of five representative molecules (**S1 Table**), and revealed that D2R and RGS must be balanced within a narrow concentration range, which was consistent with their age-dependent co-increase observed in an experiment [28]. The balance requirement appeared to be valid under both non-competitive and competitive G_i_ inhibition of AC, and theoretical analyses further proved the universality of the balance requirements. If the balance was biased toward D2R as in schizophrenia [29–32], the increased D2R activity produced excess amount of G_i_-GTP, disrupting the DA-dip detection for LTP. If the balance was biased toward RGS as in the case of DYT1 dystonia [28], the smaller amount of G_i_-GTP also disturbed the DA-dip detection. We further discuss the relationship among the D2R–RGS balance, LTP, and disease mechanisms.

## Methods

### Overview of modeling

**Fig 1:**
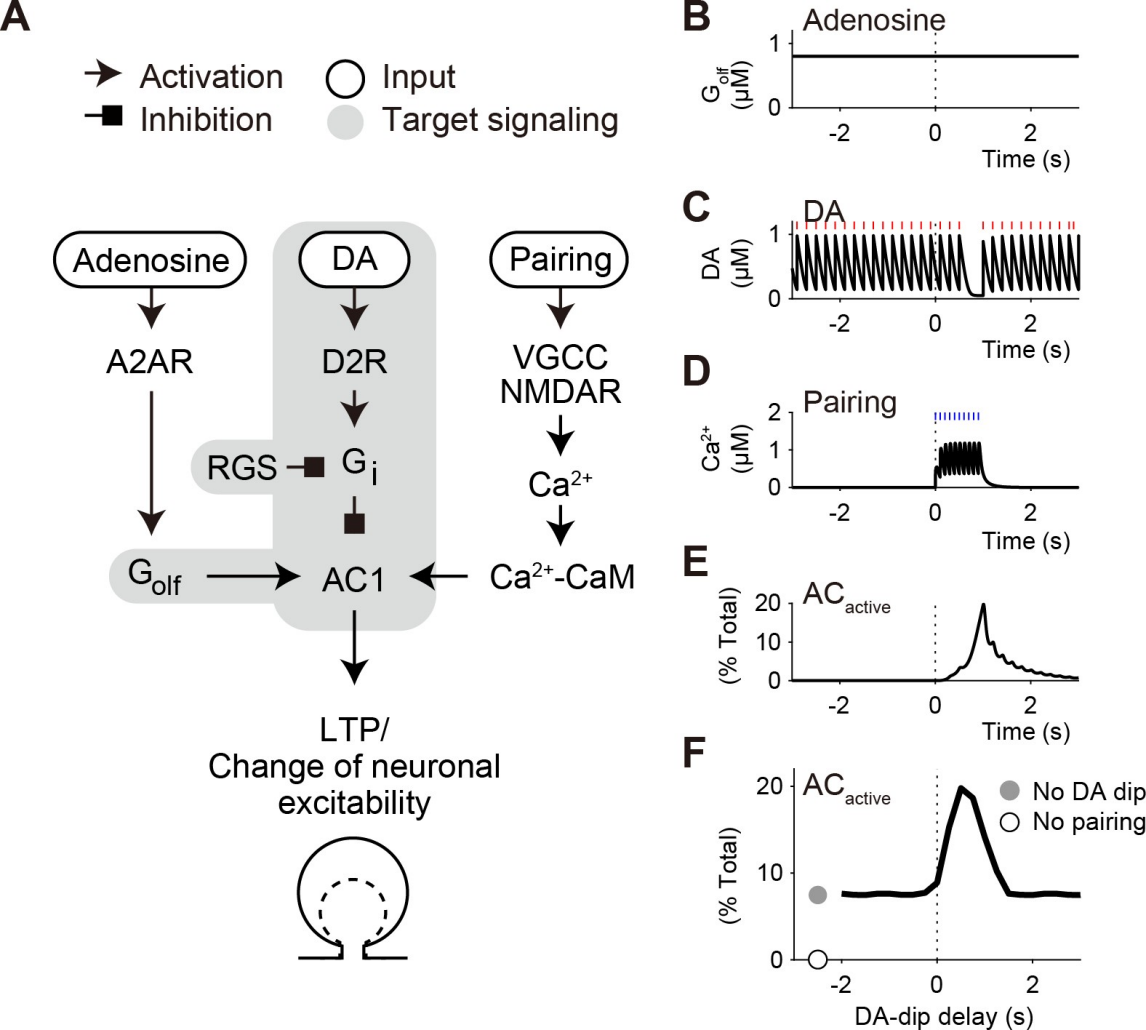
The D2 model performs the coincidence detection between DA dip and pre–post pairing. (A) Three signaling cascades toward AC1 for LTP in D2 SPNs of the striatum [10]. First, adenosine stimulates A2AR, which produces G_olf_-GTP to activate AC1. Second, basal DA signal leads to the activity of D2R, and then produces a GTP from of G_i_ (G_i_-GTP), which inhibits the AC1 activity. Third, pre–post pairing signal leads to the postsynaptic increase in Ca^2+^-CaM that stimulates AC1. Signaling in the gray shaded area is modeled in the present D2 model. (B-F) Coincidence detection between DA dip and pre–post pairing. (B) In Iino et al. (2020), A2AR were pharmacologically activated to give a continuous signal of G_olf_-GTP [1]. (C) DA fibers are optogenetically stimulated tonically at 5Hz (red lines). The tonic stimulation accompanies a 0.5-s pause (DA dip, *t*_DA,delay_ = 0.5 s), as a representation of unexpected reward omission. (D) Sensory/action signals are represented by pre–post pairing at 10 times and 10 Hz (blue lines), which gives a transient Ca^2+^ signal. (E) The G_olf_, G_i_, and Ca^2+^-CaM signals transiently activates AC1 (*AC*_active_). (F) A timing window for DA-dip delay on the maximal amplitudes of *AC*_active_. The D2 model is simulated under the non-competitive binding among G_olf_, G_i_, and Ca^2+^-CaM (standard non-competitive model; see **S1 Fig and section A in S1 Appendix**).

We selected the D2R–G_i_–AC part from the previous kinetic model of LTP in striatal D2 SPNs (**Fig 1A**, gray shaded area) [10], and examined the “D2 model” to address whether DA-dip signal was transmitted to AC with a time resolution of 0.5~2 s [3,4]. In our primary target experiment [1], D2 SPNs accepted three types of input stimulation: and a continuous pharmacological activation of adenosine A2A receptors (A2AR; **Fig 1B**), tonic DA signal and its pause (DA dip; **Fig 1C**), and phasic pre–post pairing (**Fig 1D**). According to the knowledge of intracellular signaling [9], the tonic signal of DA activates D2R, leading to the GDP/GTP exchange in G_i_. The produced G_i_-GTP inhibits the activity of AC1, in particular, in the presence of G_βγ_ (**S1 Table**) [18]. The G_i_-GTP is rapidly hydrolyzed due to GTPase activating proteins, especially, RGS9–2 in the striatum [27,34,35]. Thus, a pause of tonic DA increases the GDP-bound form of G_i_ (G_i_-GDP), which results in the detachment of G_i_-GDP from AC1, and the G_i_-free AC1 is disinhibited. In contrast, pre–post pairing generates a transient signal of Ca^2+^/calmodulin (Ca^2+^-CaM) (**Fig 1D**), and A2AR activity produces a continuous signal of G_olf_-GTP (**Fig 1B**). The Ca^2+^-CaM and G_olf_-GTP both bind and activate AC1 in a synergistic manner (**Fig 1E**) [14,15]. The AC1 activity produces cAMP, which activates cAMP-dependent protein kinase (PKA), and enhances the induction of LTP or other neuronal functions. The DA-dip also disinhibits another subtype of AC, AC5 [20,24]. The disinhibited AC5 is activated by the binding of G_olf_-GTP alone [18,36], and Ca^2+^-CaM is not required for the activation (**S1B and S2B Figs**) [37]. We chose the shared part of both AC1 and AC5 as the D2 model to examine the DA-dip detectability in AC (see the subsection “Readouts”).

In the following subsection, we described the basic principles of kinetic formulation, input signals, and readouts of the D2 model. The detailed description of the D2 model is provided in **section A in S1 Appendix** and **S1 and S2 Figs**, and molecular concentrations and kinetic constants are summarized in **S1 and S2 Tables**, respectively. Compared to the previous D2 LTP model, some parameters were updated according to experimental evidence. We thus confirmed that the updated set of parameters provided a time window as shown in the previous D2 LTP model (**Fig 1F**) [10], and it was used as a standard set of parameters. Computer simulation of the D2 model was carried out using MATLAB SimBiology (R2020a; MathWorks). The developed MATLAB code and its SBML-style files are available at the public repository GitHub (https://github.com/urakubo/ModelRP2.git).

### Binding and enzymatic reactions

All molecular interactions in the D2 model were represented by binding and enzymatic reactions under the mass assumption [10,38,39]. In the formulation, “:” denotes non-covalent binding between molecules. G_β_:G_γ_ is exceptionally denoted by G_βγ_, because G_β_:G_γ_ is known to form a tight complex [40]. GTP and GDP forms of G_X_ are represented by G_X_-GTP and G_X_-GDP, respectively, and Ca^2+^/calmodulin is represented by Ca^2+^-CaM. [*X*] denotes time-varying molecular concentration, [*X*]_tot_ denotes the total concentration, and [*X*]_buff_ denotes the buffered concentration. [*X*]_basal_ denotes molecular concentration at the basal state (*d*[*X*]/*dt* = 0 under [*DA*] = [*DA*]_basal_), [*X*]_dip_ denotes molecular concentration at the DA-dip state (*d*[*X*]/*dt* = 0 under [*DA*] = [*DA*]_dip_). [*DA*]_basal_ and [*DA*]_dip_ are described in the subsection “Inputs.”

A binding reaction in which *A* binds to *B* to form *A*:*B* was expressed by the following equation:

$$A+B\begin{array}{c} k_{\mathrm{on}}\\ ⇄\\ k_{\mathrm{off}} \end{array}A:B,$$

$$\frac{d[A:B]}{dt}=k_{on}[A][B]-k_{off}[AB],$$ (1)

where *k*_on_ and *k*_off_ are the rate constants for the forward and backward reactions, respectively. Here, *k*_off_/*k*_on_ is known as the dissociation constant, *K*_d_. Enzymatic reactions were modeled based on the Michaelis-Menten (MM) equation:

$$S+E\overset{\;K_{m},k_{\mathrm{cat}}\;}{\to }P+E,$$

$$\frac{d[P]}{dt}=\frac{k_{\mathrm{cat}}[E][S]}{K_{m}+[S]},$$ (2)

where *S*, *E*, and *P* denote substrate, enzyme, and product, respectively, and *K*_m_ and *k*_cat_ are the Michaelis constant and product turnover rate, respectively. We did not consider *E–S* complexes for simplicity, similarly to a previous study [41].

### Inputs

**Fig 2:**
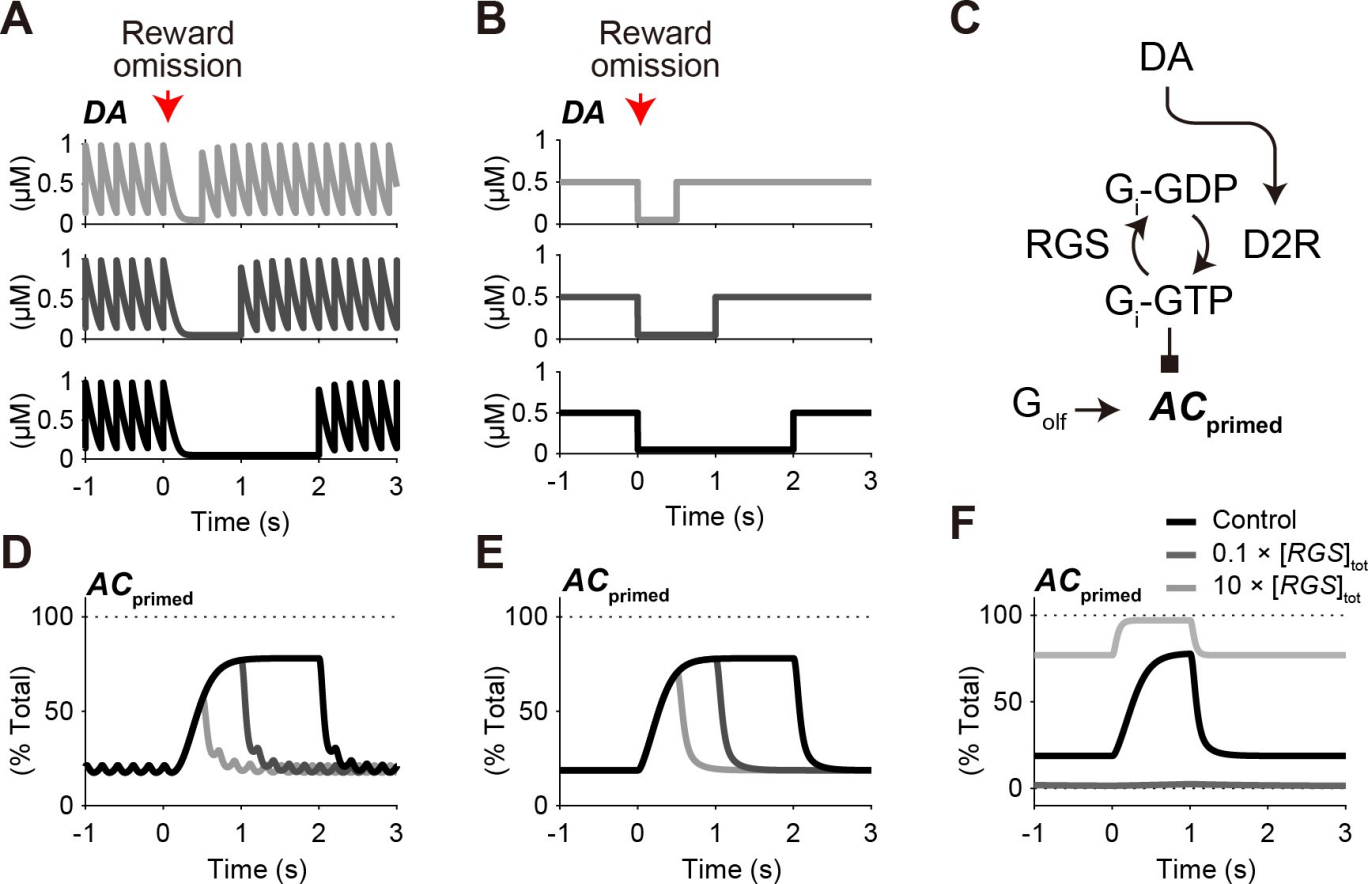
Characterization of optogenetically-evoked DA dynamics as a square wave dip. (A) Simulation of optogenetically-evoked DA dynamics (5 Hz) and their pauses for 0.5 s, 1.0 s, and 2.0 s (top, middle, and bottom, respectively). The tonic optogenetic stimulation is set to generate 0.5 μM DA on average, and the DA pause is set to give 0.05 μM DA at bottom levels (see Methods). (B) Characterization of the optogenetic DA signals as square wave dips. (C) Introduction of *AC*_primed_ as the G_i_-free and G_olf_-bound state of AC. Further Ca^2+^ stimulation leads to the activity of AC1. In the case of AC5, *AC*_primed_ corresponds to a normalized activity of AC. (D) Dynamics of *AC*_primed_ in response to the optogenetically-evoked DA signals. (E) Dynamics of *AC*_primed_ in response to the square-wave DA dips. (F) Appropriate range of [*RGS*]_tot_ is required for the response of *AC*_primed_. Square-wave DA dips are given with a duration of 1 s. *AC*_primed_ reaches almost the maximal level even with the basal DA signal if [*RGS*]_tot_ is set to be 10 times higher than the standard concentration (9 μM, light gray line), and *AC*_primed_ does not show any increase if [*RGS*]_tot_ is 10 times lower than the standard concentration (0.09 μM, dark gray line).

In Iino et al. (2020), channelrhodopsin-2-expressed DA fibers were stimulated with 5-Hz light stimulation with a 0.4-s pause, and the pause signal was successfully observed as a DA-dip signal of extracellular DA dynamics [1]. However, the penetration of an observation probe (5~8 μm diameter) might interfere with the DA dynamics, because the cycle of DA release, diffusion, and uptake is known to occur only in a span of ~10 μm [42]. Recent DA observation using an ultrafast fluorescent probe shows the faster dynamics of DA (*t*_1/2_ ~ 0.1 s) [43,44]. Thus, based on a preceding model [45], we first simulated the rapid concentration dynamics of DA, [*DA*], as follows (**Figs 1C and 2A**):

$$\{t_{\mathrm{DA}}^{i}{\}}_{i=-\infty }^{\infty }=\{\cdots ,-0.4\;s,-0.2\;s,0\;s,T_{\mathrm{dip}},T_{\mathrm{dip}}+0.2\;s,T_{\mathrm{dip}}+0.4\;s,\cdots \},$$

$$\frac{d}{dt}[DA]={\left[DA\right]}_{\mathrm{opto}}\sum_{i}\delta (t-t_{\mathrm{DA}}^{i}-t_{DAdelay})-\frac{k_{\mathrm{cat},\mathrm{DAT}}K_{m,\mathrm{DAT}}[DAT]({\left[DA]-[DA\right]}_{dip})}{\left(K_{m,\mathrm{DAT}}+[DA])(K_{m,\mathrm{DAT}}+{\left[DA\right]}_{dip}\right)},$$ (3)

where *T*_dip_ is the duration of a DA pause, *t*_DA,delay_ is the onset time of the DA pause, [*DA*]_dip_ is the bottom concentration of produced DA dip, and [*DA*]_opto_ is the amplitude of DA signal by a single light pulse. *k*_cat,DAT_[*DAT*] and [*DA*]_opto_ were determined so as to give an average concentration of 0.5 μM and a half-valued period of 0.1 s [1,44].

We next characterized the DA-dip signal as a square wave dip of [*DA*] (**Fig 2B**):

$$[DA]=\{\begin{array}{ll} {\left[DA\right]}_{basal} & \left(t<0,T_{dip}\leq t\right)\\ {\left[DA\right]}_{dip} & \left(0\leq t<T_{dip}\right) \end{array},$$ (4)

Here, we set [*DA*]_basal_ = 0.5 μM and [*DA*]_dip_ = 0.05 μM unless otherwise stated (**Fig 2B**). Those levels of DA have been estimated based on a reference experiment (**S1 Table**) [1]. We finally gave a stepwise-decreasing signal of DA to quantify the DA-dip detection in the response of AC:

$$[DA]=\{\begin{array}{ll} {\left[DA\right]}_{\mathrm{basal}} & \left(t<0\right)\\ {\left[DA\right)}_{\mathrm{dip}} & \left(t\geq 0\right) \end{array}.$$ (5)

In Iino et al. (2020), A2AR was continuously activated to produce G_olf_-GTP with a constant rate [1]. The G_olf_-GTP is known to bind to a specific site of AC, while it is autonomously hydrolyzed and detached from the AC [46]. The dynamics of G_olf_-GTP was simply modeled as a constant concentration of buffered G_olf_, [*G*_olf_]_buff_ (**Fig 1B**).

We also simulated a pre–post pairing-induced dynamics of Ca^2+^. The pre–post pairing was constituted of 10-consecutive elemental pairs at 10 Hz (**Fig 1D**):

$$\{t_{\mathrm{pre}-\mathrm{post}}^{i}{\}}_{i=1}^{10}=\{0\;s,0.1s,0.2\;s,\ldots ,0.9\;s\}.$$ (6)

Each pairing led to Ca^2+^ influx via voltage-gated Ca^2+^ channels (VGCC) and *N*-Methyl-*D*-aspartate receptors (NMDAR). The Ca^2+^ bound to CaM, while free Ca^2+^ was uptaken by a Ca^2+^ pump. The Ca^2+^-CaM further bound to a specific site of AC. Detailed scheme and parameters of the Ca^2+^ signal are described in Urakubo et al. (2020) [10], and the MATLAB code of the full D2 model is available at the public repository GitHub (https://github.com/urakubo/ModelRP2.git).

### Readouts

G_olf_ and Ca^2+^-CaM synergistically activate AC1 [14,15], and G_olf_ alone sufficiently activate AC5 [37]. The G_olf_-dependent activity of AC5 is inhibited by G_i_, and the Ca^2+^-CaM-dependent component of AC1 activity is also inhibited by G_i_, while the G_olf_-dependent component of AC1 activity is only weakly inhibited by G_i_ [18]. We here assumed that the G_olf_- and Ca^2+^-CaM-dependent synergistic activity of AC1 was strongly inhibited by G_i_. G_olf_, G_i_, and Ca^2+^-CaM are known to have their specific binding sites at AC1/5 [36,47,48], and molecular dynamics simulation shows that AC5 forms an inactive ternary complex with G_olf_ and G_i_ [20]. Those pieces of evidence support the idea that G_i_ non-competitively inhibits AC, i.e., G_olf_, G_i_, and Ca^2+^-CaM independently bind to AC, and the G_i_-binding itself inhibits the enzymatic activity of AC (non-competitive inhibition; **S1 Fig**). In contrast, biochemical studies suggest that G_i_ competitively inhibits AC5 [36,49], i.e., G_i_ inhibits the AC activity by prohibiting the binding of activators, i.e., G_olf_ and Ca^2+^-CaM (**S2 Fig**). In short, the mechanism of G_i_ inhibition is currently obscure even in the case of well-studied AC5. Considering this situation, we examined two extreme cases of the G_i_ inhibition: 100% non-competitive binding among G_i_, G_olf_, and Ca^2+^-CaM, (standard non-competitive model) and 100% competitive binding between G_i_ and the other two molecules (competitive model), and *AC*_primed_ was introduced as a shared readout of AC1 and AC5.

#### Standard non-competitive model

The concentration of active-state AC, [*AC*_active_], was defined by the following equation (**S1 Fig**):

$$\frac{\left[{AC}_{\mathrm{active}}\right]}{{\left[AC\right]}_{tot}}=\{\begin{array}{ll} \frac{\left[{AC}_{i}^{site}\right]}{{\left[AC\right]}_{tot}}∙\frac{\left[{AC}_{\mathrm{olf}}^{\mathrm{site}}:G_{olf}\right]}{{\left[AC\right]}_{tot}}∙\sum_{i}\frac{\left[{AC}_{\mathrm{CaM}}^{\mathrm{site}}:{{Ca}^{2+}‐CaM}_{i}\right]}{{\left[AC\right]}_{tot}} & \left(AC1\right)\\ \frac{\left[{AC}_{i}^{site}\right]}{{\left[AC\right]}_{tot}}∙\frac{\left[{AC}_{\mathrm{olf}}^{\mathrm{site}}:G_{olf}\right]}{{\left[AC\right]}_{tot}} & \left(AC5\right) \end{array},$$ (7)

where ${AC}_{i}^{\mathrm{site}}$, ${AC}_{\mathrm{olf}}^{\mathrm{site}}$, and ${AC}_{\mathrm{CaM}}^{\mathrm{site}}$ denote the binding sites of AC for G_i_, G_olf_, and Ca^2+^-CaM, respectively, and *i* = 1,…,9 denote the states of Ca^2+^-CaM. Here, $\left[{AC}_{i}^{\mathrm{site}}\right]$ denotes the concentration of G_i_-free AC, i.e., $\left[{AC}_{i}^{\mathrm{site}}]={\left[AC\right]}_{\mathrm{tot}}-[{AC}_{i}^{\mathrm{site}}:G_{i}\right]$. We then selected the shared part of AC1 and AC5, as a primed state of AC for the activity:

$${AC}_{\mathrm{primed}}=\frac{\left[{AC}_{i}^{site}\right]}{{\left[AC\right]}_{tot}}∙\frac{\left[{AC}_{\mathrm{olf}}^{\mathrm{site}}:G_{olf}\right]}{{\left[AC\right]}_{tot}}∙\frac{K_{d,\mathrm{Golf}}+{\left[G_{\mathrm{olf}}\right]}_{buff}}{{\left[G_{\mathrm{olf}}\right]}_{buff}}.$$ (8)

where [*G*_olf_]_buff_/{*K*_d,Golf_+[*G*_olf_]_buff_} denotes the binding ratio of AC to G_olf_. If [*G*_olf_]_buff_ is a constant,

$$\frac{\left[{AC}_{\mathrm{olf}}^{\mathrm{site}}:G_{olf}\right]}{{\left[AC\right]}_{tot}}=\frac{{\left[G_{\mathrm{olf}}\right]}_{buff}}{K_{d,\mathrm{Golf}}+{\left[G_{\mathrm{olf}}\right]}_{buff}}.$$ (9)

Thus, *AC*_primed_ is simply $\left[{AC}_{i}^{site}\right]/{\left[AC\right]}_{tot}$ under the constant [*G*_olf_]_buff_. All simulation and analyses were conducted on the standard non-competitive model unless otherwise stated.

#### Competitive model

We defined the concentration of active-state AC, [*AC*_active_], under the situation of 100%-competitive binding between G_i_ and the other two molecules, G_olf_ and Ca^2+^-CaM (**S2 Fig**):

$$\frac{\left[{AC}_{\mathrm{active}}\right]}{{\left[AC\right]}_{tot}}=\{\begin{array}{ll} \sum_{i}\frac{\left[G_{olf}:AC:{{Ca}^{2+}‐CaM}_{i}\right]}{{\left[AC\right]}_{tot}} & \left(AC1\right)\\ \frac{\left[G_{olf}:AC\right]}{{\left[AC\right]}_{tot}} & \left(AC5\right) \end{array},$$ (10)

where [*G*_olf_: *AC*: *Ca*^2+^-*CaM*_*i*_] denotes the concentration of AC that binds to both G_olf_ and Ca^2+^-CaM, but not to G_i_. The G_olf_ binding and G_i_ unbinding are shared requirements for the activities of AC1 and AC5 (**S2 Fig**). We thus picked up the shared part of AC1 and AC5, as a primed state of AC for the activity, *AC*_primed_, as:

$${AC}_{\mathrm{primed}}=\frac{\left[G_{olf}:AC\right]}{{\left[AC\right]}_{tot}}∙\frac{K_{d,\mathrm{Golf}}+{\left[G_{\mathrm{olf}}\right]}_{buff}}{{\left[G_{\mathrm{olf}}\right]}_{buff}},$$ (11)

In both non-competitive and competitive models, *AC*_primed_ was set to be a readout. *AC*_primed_ is a dimensionless value (0 ≤ *AC*_primed_ ≤ 1), and 0% ≤ *AC*_primed_ ≤ 100% was used in the description. We also introduced *AC*_basal_ and *AC*_dip_ to represent two steady states of *AC*_primed_, i.e., ${AC}_{basal}=\underset{t\to \infty }{\lim}{AC}_{primed}(t)$ where [*DA*] = [*DA*]_basal_, and ${AC}_{dip}=\underset{t\to \infty }{\lim}{AC}_{primed}(t)$ where [*DA*] = [*DA*]_dip_.

### DA-dip duration dependence in *AC*_primed_

Here, we quantified the DA-dip duration-dependent response of *AC*_primed_ using the following equation:

$${\langle {AC}_{primed}\rangle }_{Tdip}=\frac{1}{T_{dip}}\int_{0}^{T_{dip}}{AC}_{primed}(t)dt.$$ (12)

〈*AC*_primed_〉_Tdip_ represents the average increase in *AC*_primed_ during the DA-dip period [0, *T*_dip_] in Eq (4).

### Concentrations of D2R and RGS under healthy and pathologic conditions

Four pairs of [D2R]_tot_ and [RGS]_tot_ were set to represent a healthy adult, healthy infant, and patients with DYT1 dystonia and schizophrenia. [D2R]_tot_ and [RGS]_tot_ in the standard set of parameters were used for the healthy-adult model, and 0.5 × [*D2R*]_tot_ and 0.5 × [*RGS*]_tot_, were set for the healthy-infant model, because Bonsi et al. have observed a ~0.4-fold simultaneous decrease in their expression levels in the striatum of infant mice (postnatal day 7) [28]. Such a decrease has been seen in other studies as well [50,51]. A parameter set of 4.0 × [*D2R*]_tot_ and 0.5 × [*RGS*]_tot_ was used for the schizophrenia model, because D2 blockers need to occupy 70~85% of the total D2R to give clinical effects while avoiding side effects [52,53], and the level of RGS seems to decrease by 10~75% in schizophrenia [31,32]. A parameter set of 0.5 × [*D2R*]_tot_ and 2.0 × [*RGS*]_tot_ was used for the dystonia model, because Bonsi et al. have observed a 0.7-fold D2R decrease and 1.6-fold RGS increase in the protein expression levels of the striatal detergent-resistant membranes (DRM) in a mouse model of DYT1 dystonia [28], and another study has shown a ~0.25-fold decrease in the activity of striatal D2R in another mouse model of DYT1 dystonia [54]. Note that those values were determined only for exemplifying purpose, and the actual concentrations depend on the subjects.

## Results

### Characterization of DA pause as a square wave dip

The updated D2 model was first simulated to confirm the occurrence of a time window for DA-dip delay on the activity of AC1 (**Fig 1**) [10]. In this simulation, we applied the D2 model to three types of inputs (**Fig 1A**): a constant signal of G_olf_ (**Fig 1B**), optogenetically-evoked tonic DA signal with a 0.5-s pause (**Fig 1C**), and 1-s pre–post pairing (**Fig 1D**). Those inputs resulted in a transient activity of AC1, *AC*_active_ (**Fig 1E**), and the AC1 activity depended on the delay of DA pause, *t*_DA,delay_ (**Fig 1F**), as shown in the previous D2 LTP model [10]. The activity of AC1 is known to produce cAMP, leading to neuronal functions such as synaptic plasticity (**Fig 1A**).

Among the three input signals, DA pause is particularly interesting. The rapid dynamics of DA resulted in a fluctuating DA signal even under the basal state, and the 0.5-s DA pause appeared to be a minor event (**Fig 1C**). We thus examined how the DA-pause signal was transmitted to AC, by characterizing it as a square wave dip of DA (**Fig 2A and 2B**), and the normalized level of G_i_-free and G_olf_-bound AC, *AC*_primed_, was observed as a readout (see Methods; **Fig 2C**). In the dynamics of *AC*_primed_, the optogenetically-evoked signals of DA were well characterized by the square wave dips of DA (**Fig 2D and 2E**), because the fluctuation in the basal DA signal was attenuated through the D2R−G_i_−AC signaling pathway (**S3 Fig**).

The response of *AC*_primed_ was dependent not only on the DA dynamics, but also on the concentrations of other constituent molecules (**Fig 2F**). If the concentration of RGS, [*RGS*]_tot_, was set to be 10 times higher than the standard concentration (**S1 Table**), *AC*_primed_ showed ~80% of the maximal activity even with the basal DA signal (**Fig 2F**, light gray line). In this case, there was only small room for further activation. By contrast, if [*RGS*]_tot_ was set to be 10 times lower than the standard concentration, *AC*_primed_ did not show any activity even during the DA dip (**Fig 2F**, dark gray line). We thus raised a next question: what are requirements on the parameters, i.e., molecular concentration and kinetic constants, for the DA-dip detection?

### Amplitudes of *AC*_primed_ for DA-dip detection

**Fig 3:**
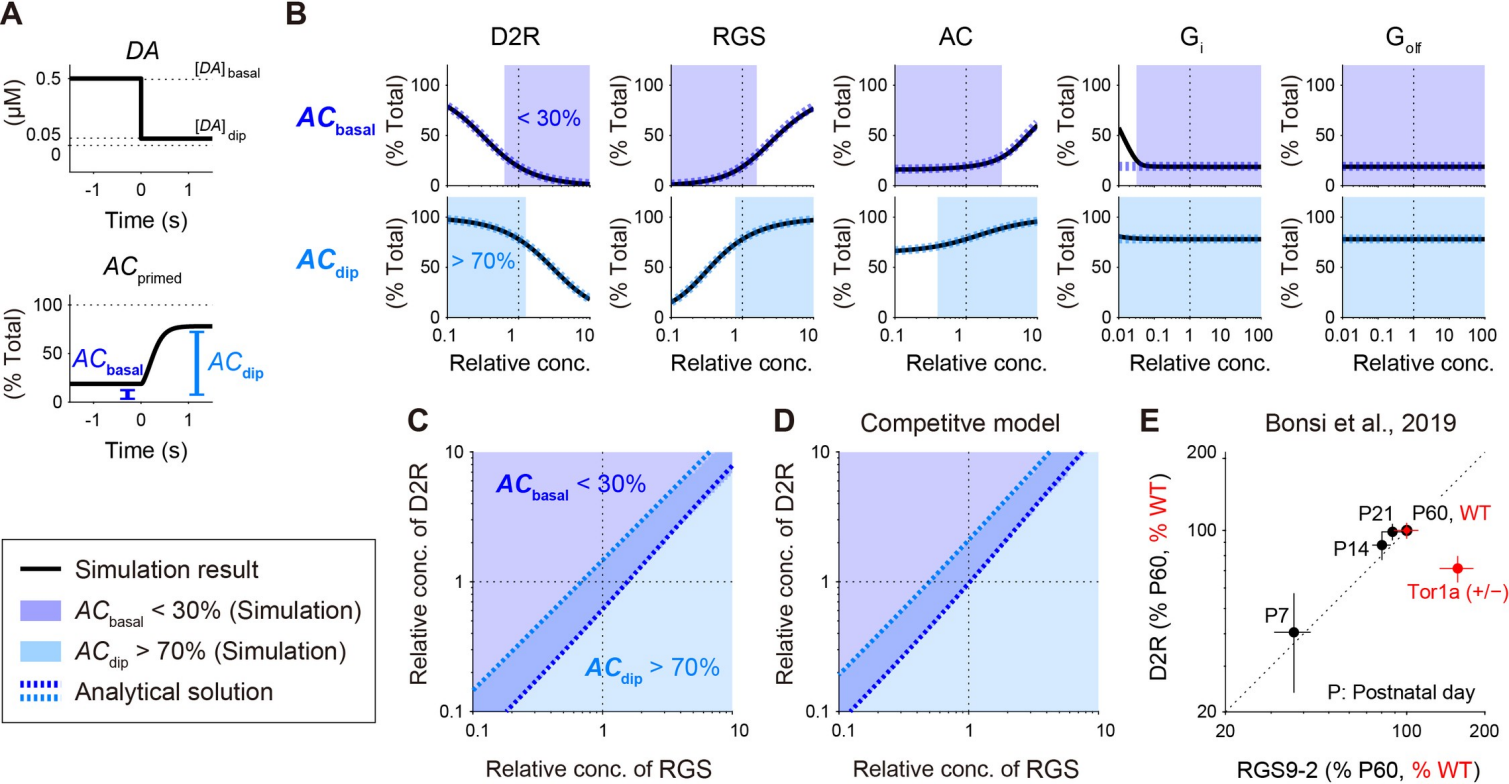
DA-dip detectable region appears between two increasing boundaries in the space of D2R and RGS. (A) Introduction of two measures, *AC*_basal_ and *AC*_dip_, to quantify DA-dip detectability. *AC*_primed_ under the basal DA signal, *AC*_basal_, should be low, whereas that during a DA dip, *AC*_dip_, should be high. (B) Concentration requirements for the DA-dip detection under the two measures. While D2R, RGS, AC, G_i_, and G_olf_ are targeted, *AC*_basal_ and *AC*_dip_ are measured under the altered concentrations of one of the target molecules. Simulation results (black solid lines) and analytical solutions (blue and light blue dotted lines, Eq (23)) are plotted. *AC*_basal_ < 30% and *AC*_dip_ > 70% are highlighted as the regions necessary for DA-dip detection. (C) *AC*_basal_ < 30% and *AC*_dip_ > 70% in the space of [*D2R*]_tot_ and [*RGS*]_tot_. Analytical isolines of *AC*_basal_ = 30% and *AC*_dip_ = 70% are overlaid. (D) Same as panel C, but the D2 model is based on the competitive binding between G_olf_ and G_i_ (See **S2 Fig**). (E) Age-dependent expression levels of RGS9–2 and D2R proteins (black points) and their altered levels in a mouse model of DYT1 dystonia (*Tor1a* (+/−), red points) in Bonsi et al. [28]. Data are taken from the DRM fraction of the mouse striatum (Figs 1A and 2C of Bonsi et al. [28]; modified under Creative Commons Attribution 3.0). P7,…, P60 denote mouse postnatal days. Data are normalized by the expression levels in P60 or wild type (WT).

Then, using a stepwise decreasing signal of DA (Eq (4)), we examined the molecular concentrations required for DA-dip detection (**Fig 3A**, top). Here, the DA-dip detectability was quantified by two variables: *AC*_basal_ and *AC*_dip_ (**Fig 3A**). *AC*_basal_ denotes the steady-state level of *AC*_primed_ under [*DA*] = [*DA*]_basal_ (**Fig 3A**, dark blue; see Methods), and *AC*_dip_ denotes the steady-state level of *AC*_primed_ under [*DA*] = [*DA*]_dip_ (**Fig 3A**, light blue). We observed *AC*_basal_ and *AC*_dip_ if the concentration of one of the five constituent molecules, [*D2R*]_tot_, [*RGS*]_tot_, [*AC*]_tot_, [*G*_i_]_tot_, and [*G*_olf_]_buff_, was varied ranging from 0.1-fold to 10-fold, while the concentrations of other molecules were kept unchanged (**Fig 3B** and **S1 Table**). First, we observed that neither *AC*_basal_ nor *AC*_dip_ was sensitive to [*G*_i_]_tot_ and [*G*_olf_]_buff_ if they were higher than 4% of the standard concentration (**Fig 3B**, second right and right, black lines), while *AC*_basal_ and *AC*_dip_ were both highly sensitive to [*D2R*]_tot_ and [*RGS*]_tot_ (**Fig 3B**, left and second left, black lines). This is because the concentrations of D2R and RGS determined the available amount of G_i_-free AC for the activity. Here, for convenience, the regions that satisfies *AC*_basal_ < 30% and *AC*_dip_ > 70% were highlighted as the regions that enabled DA-dip detection (blue and light-blue shaded areas, respectively; **Fig 3B**). In the cases of [*D2R*]_tot_, [*RGS*]_tot_, and [*AC*]_tot_, the regions that satisfied *AC*_basal_ < 30% (blue) and those that fulfilled *AC*_dip_ > 70% (light blue) occupied the opposite ends of the concentrations, and the intersection of the two regions satisfying both of them were limited within narrow concentration ranges. Note that the requirement of higher [*D2R*]_tot_ for smaller *AC*_basal_ has been shown as the requirement of higher [*DA*]_basal_ in a previous study (**Fig 3B**, left, top) [19], and the requirement of lower [*D2R*]_*tot*_ for higher *AC*_dip_ has also been shown as the requirement of lower [*DA*]_dip_ in another study [24].

Because the windows for DA detection in [*D2R*]_tot_ and [*RGS*]_tot_ were particularly narrow, we further plotted their two-way relationship in a 2D space (**Fig 3C**). The DA-dip detectable region in [*D2R*]_tot_ had a clear positive relationship with that in [*RGS*]_tot_; the higher [*D2R*]_tot_ requires the higher [*RGS*]_tot_ for the detection of a DA dip (**Fig 3C**). Very interestingly, Bonsi et al. (2019) have shown that the expression level of striatal RGS9–2 has a similar positive relationship with that of D2R (**Fig 3E**, black points) in postnatal development of mice, during which both their expressions are increased [28]. They have further examined a DYT1 dystonia model (*Tor1a*^+/−^-knock-out mice), and it shows a simultaneous decrease in the gross expression levels of D2R and RGS9–2. However, because DYT1 dystonia alters protein trafficking, the expression level of RGS9–2 is selectively increased in the fraction of DRM where D2Rs is located [28,55], and the perpendicular change appeared in the space of D2R and RGS9–2 (**Fig 3D**, red points). In the scheme of the D2 model, the disruption of the D2R–RGS balance makes DA dip undetectable, thus DYT1 dystonia cannot show DA-dip dependence in striatal LTP.

The requirement of the D2R–RGS balance appeared under [*DA*]_basal_ = 0.5 μM and [*DA*]_dip_ = 0.05 μM. The concentrations of DA were determined based on experimental measurements (**S1 Table**) [1]; however, at least [*DA*]_basal_ is known to depend on the specific situation [56,57]. We thus simulated multiple cases of [*DA*]_basal_ and [*DA*]_dip_, and found that they affected the regions of *AC*_basal_ < 30% and *AC*_dip_ > 70%, and the region that satisfied both of them disappeared depending on the pair of [*DA*]_basal_ and [*DA*]_dip_ (**S5 Fig**). Nevertheless, the DA-dip detectable region in [*D2R*]_tot_ always had a positive relationship with that in [*RGS*]_tot_ (**S5A and S5B Fig**; left), and the D2R–RGS balance was always required regardless of the pair of [*DA*]_basal_ and [*DA*]_dip_, if we considered analytical solutions that explain these boundaries (**S5 Fig**, dotted lines; see the subsection “Analytical formulation”).

All the simulations so far were based on the D2 model under 100%-non-competitive binding between G_olf_ and G_i_ (standard non-competitive model; **S1 Fig** and **section A in S1 Appendix**). It is known that G_olf_ stimulates both AC1 and AC5, and G_i_ inhibits their activities [58]. However, even in well studied AC5, it is still under investigation whether AC is inhibited by G_i_ through non-competitive inhibition or the allosteric exclusion of G_olf_ binding [20,59]. We thus simulated the D2 model under 100%-competitive binding between G_olf_ and G_i_ (competitive model; **S2 Fig** and **section A in S1 Appendix**). The simulation results were similar to those in the standard non-competitive model (**S4A and S4B Fig**), and the requirements of the D2R–RGS balance also appeared only with a slight bias toward RGS (**Fig 3D**). The actual G_i_ inhibition should fall in between the 100%-competitive and non-competitive models. Thus, the requirements of the D2R–RGS balance was robust to the mechanisms of G_i_ inhibition.

In the simulation, the concentration of G_olf_ affected *AC*_primed_ only in the competitive model (**Figs 3B and S4B**, right). This is because the binding of G_olf_ to AC decreased the availability of G_olf_-free AC for G_i_ inhibition, and the increase in [*G*_olf_]_buff_ led to a simultaneous increase in *AC*_basal_ and *AC*_dip_, which decreased the dynamic range of *AC*_primed_ (**S4B Fig**, right). Conversely, if [*G*_olf_]_buff_ was set to be low, the dynamic range was restored (**S4B Fig**, right); however, the maximal activity of AC became small. As a result, there appeared to be an optimal [*G*_olf_]_buff_ for Δ*AC*_active_ where ${\Delta AC}_{active}={{AC}_{active}|}_{[DA]={\left[DA\right]}_{dip}}{{-AC}_{active}|}_{[DA]={\left[DA\right]}_{basal}}$ (**S4C Fig**). It was consistent with the simulation results in a previous study [20].

### Rapid response of *AC*_primed_ for DA-dip detection

**Fig 4:**
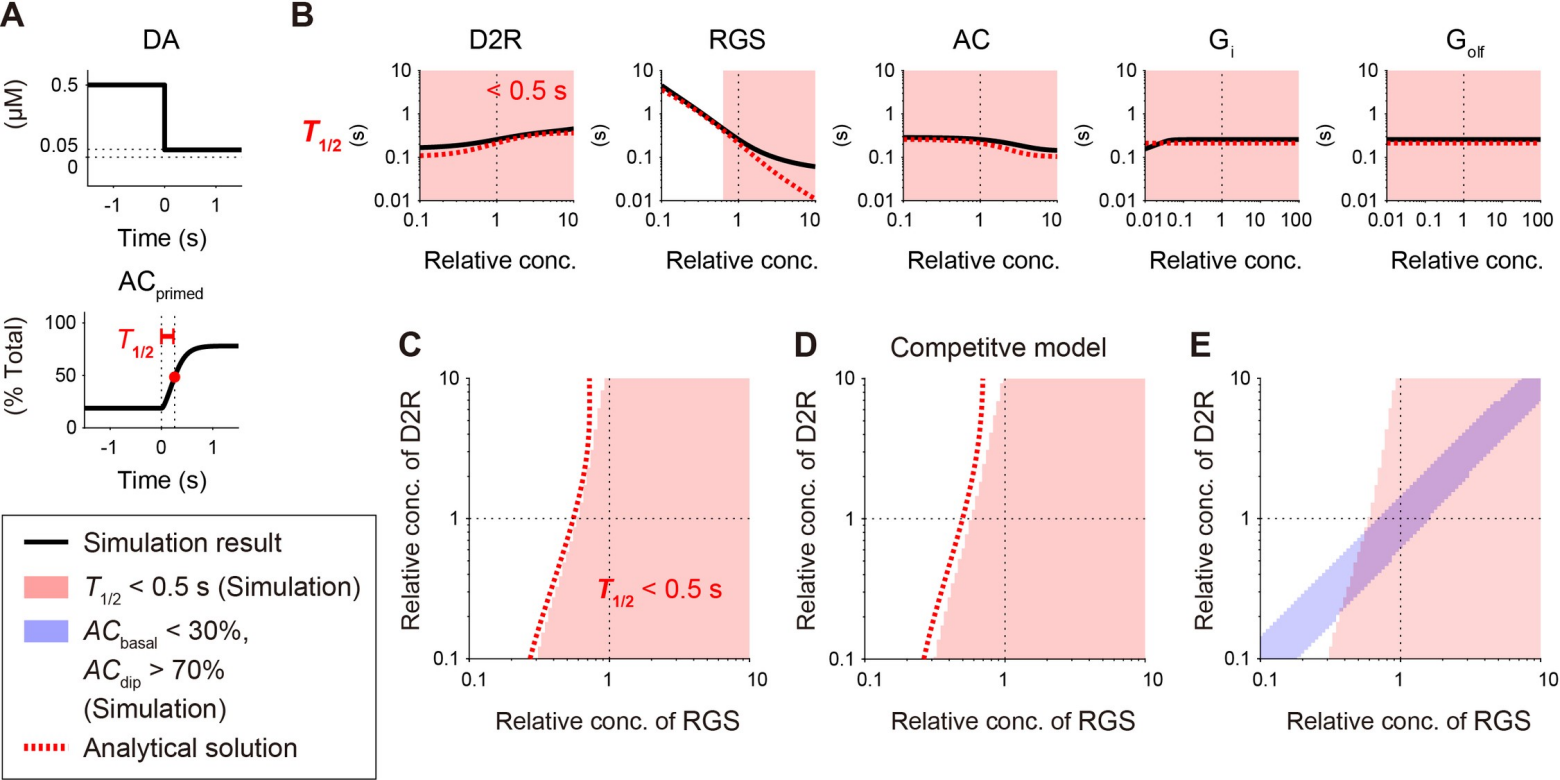
DA dip detection requires a certain concentration of RGS. (A) The third measure to quantify DA-dip detectability. Sudden decrease in the basal DA signal disinhibits AC with a half-maximal time, *T*_1/2_, which needs to be small for detection of a short (~0.5 s) DA-dip. (B) Concentration dependence of *T*_1/2_. Five molecules were targeted, and the simulation results (black solid lines) and analytical solutions (red dotted line, Eq (24)) were plotted. *T*_1/2_ < 0.5 s were highlighted in pink as the region that allows detection of the DA dip. (C) *T*_1/2_ < 0.5 s (pink) in the space of [*D2R*]_*tot*_ and [*RGS*]_*tot*_. (D) Same as panel C, but the DA model was based on the competitive binding between G_olf_ and G_i_ (See **S1 and S2 Figs**). (E) The area of *T*_1/2_ < 0.5 s (pink shaded area in (C)) was overlapped with the region that satisfies both *AC*_basal_ < 30% and *AC*_dip_ > 70% (blue shaded area). The ~0.5-s DA dips can only be detected in the overlapping area.

The DA-dip detection depends not only on the steady-state levels of *AC*_primed_, but also on its time constant. That is, the time constant of G_i_ unbinding must be shorter than the DA-dip duration, because otherwise the DA-dip signal would not appear in the change of *AC*_primed_ [19]. We thus evaluated it using a variable, *T*_1/2_, where *T*_1/2_ (> 0 s) denotes the half maximal time of *AC*_primed_ after a sudden DA decrease (**Fig 4A**). Note that exponential fits were not utilized to quantify the increasing time constant because the *AC*_primed_ response did not always grow in an exponential manner. We obtained *T*_1/2_ using the same set of molecular concentrations as in **Fig 3B**, and found that *T*_1/2_ became less than 0.5 s only if [*RGS*]_tot_ exceeded a certain level (**Fig 4B**, red shaded areas). Then, we plotted the DA-dip detectable area in the 2D space of [*D2R*]_tot_ and [*RGS*]_tot_ and found a slight dependence on [*D2R*]_tot_ (**Fig 4C**). We finally overlaid this plot with the requirements on *AC*_basal_ and *AC*_dip_ (**Fig 4E**). [*D2R*]_tot_ and [*RGS*]_tot_ were needed to fall in the overlapping region (*AC*_basal_ < 30%, *AC*_dip_ > 70%, and *T*_1/2_ < 0.5 s) for the DA-dip detection in LTP and/or the change of neuronal excitability (**Fig 4E**). As expected, this region depended on [*DA*]_basal_ and [*DA*]_dip_ (**S5 Fig**). The higher [*DA*]_basal_ and lower [*DA*]_dip_ were required for the dynamics range in the response of *AC*_primed_, while the lower [*DA*]_basal_ was better for the rapid response (**S5A Fig**, right). The requirements of the D2R–RGS balance and high [*RGS*]_*tot*_ were preserved regardless of the concentrations of DA.

All the characteristics of *T*_1/2_ were also preserved in the competitive model (**Figs 4D and S4D**), and *T*_1/2_ in the competitive model further depended on the binding/unbinding reaction rate of G_olf_ (**S6 Fig**). This is because *AC*_primed_ represented the state of G_i_-free and G_olf_-bound *AC*, and the G_olf_ binding rate became a time liming process if the G_olf_ binding process was slower than the G_i_ unbinding process. The reaction rate of G_olf_ did not affect the requirement of the D2R–RGS balance (**S6A and S6B Fig**, center), because it did not affect the steady-state level of G_olf_, but the dissociation constant, *K*_d,Golf_, should affect it. Similar reaction-rate dependency has been examined by Bruce et al. (2019) [20].

### Analytical formulation

The D2 model revealed that DA dip could be detected only in a restricted range in the space of [*RGS*]_tot_ and [*D2R*]_tot_ (Fig 3D). However, this requirement has been demonstrated only for the standard set of parameters (**S1 and S2 Tables**), while it should also depend on the other type of parameters, i.e., kinetic constants (**S2 Table**). Similarly, we defined the DA-dip detectable region, i.e., *AC*_basal_ < 30%, *AC*_dip_ > 70%, and *T*_1/2_ < 0.5 s, mainly for convenience, and they do not necessarily take exactly these values. We thus derived their analytical solutions to examine the overall parameter dependence of *AC*_basal_ and *AC*_dip_, and *T*_1/2_.

To enable it, we first made simplification on the enzymatic reactions based on the MM formulation. We introduced the catalyst-saturated approximation, *d*[*P*]/*dt* ~ [*E*]*k*_cat_ if *K*_m_ << [*S*], to the GTP/GDP exchange of G_i_-GDP, i.e.,

(a) *K*_m,exch,Gi_ << [*G*_i_:*G*_βγ_],

where *K*_m,exch,Gi_ is the Michaelis constant, and [*G*_i_:*G*_βγ_] is the substrate concentration. The constraint (a) was based on the facts that *K*_m,exch,Gi_ ~ 10 nM (**S2 Table**) and [*G*_i_]_tot_ ~ 10 μM (**S1 Table**). Thus, in almost all the situations, *K*_m,exch,Gi_ was much lower than [*G*_i_*·G*_βγ_]. Similarly, we introduced the first-order rate approximation, *d*[*P*]/*dt* ~ [*E*][*S*]*k*_cat_/*K*_m_ if *K*_m_ >> [*S*], to the GTP hydrolysis of G_i_, i.e.,

(b) *K*_m,hyd,Gi_ >> [*G*_i_*-GTP*],

where *K*_m,hyd,Gi_ is the Michaelis constant, and [*G*_i_*-GTP*] is the substrate concentration. The constraint (b) was based on the parameters that *K*_m,hyd,Gi_ ~ 12 μM and [*G*_i_]_tot_ ~ 10 μM (**S1 and S2 Tables**). Only a subpopulation of [*G*_i_]_tot_ forms [*G*_i_*-GTP*]; thus, *K*_m,hyd,Gi_ > [*G*_i_]_tot_ > [*G*_i_*-GTP*], and *K*_m,hyd,Gi_ > [*G*_i_*-GTP*]. We further set the following constraints:

(c) [*D2R*]·[*DA*] / [*D2R*:*DA*] = *K*_d,DA_, where *K*_d,DA_ = *k*_off,DA_/*k*_on,DA_,
(d) *V*_7_ = 0 where *V*_7_ = *k*_on,GiGDP_[*AC*_i_^site^][*G*_i_*-GDP*] (see **section A in S1 Appendix**).

The constraint (c) was set because the binding of DA to D2R rapidly reaches equilibrium (*t*_1/2_ ~ 30 ms, **S2 Table**) [11,19], and the constraint (d) is the assumption that G_i_-GDP never binds to AC, which is compatible with *K*_d,GiGTP_ << *K*_d,GiGDP_ (**S2 Table**) [18]. Note that the constraint (d) was also assumed in the other D2 model as the simultaneous occurrence of G_i_-GTP hydrolysis at the time of its detachment from AC5 [19]. Simplification of the D2 model based on the constraints (a-d) is described in **section A in S1 Appendix**.

Based on the constraints (a-d), we successfully obtained the steady-state ratio of G_olf_-bound and G_i_-free forms of AC, *AC*_basal_ and *AC*_dip_, as follows (Eqs (S53) and (S61) in **section B in S1 Appendix**):

$${AC}_{basal}=\frac{-b_{basal}+\sqrt{b_{basal}^{2}-4c_{basal}}}{2},$$ (13)

$$b_{basal}=\left\{\frac{\left(k_{\mathrm{off},\mathrm{GiGTP}}+k_{RGS}\right)}{k_{\mathrm{on},\mathrm{GiGTP}}}\frac{\chi }{{\left[AC\right]}_{tot}}+(\frac{1}{k_{RGS}}+\frac{1}{k_{\mathrm{off},\mathrm{GiGDP}}})\frac{{\left[D2R\right]}_{tot}}{{\left[AC\right]}_{tot}}k_{DA,basal}-1\right\},$$

$$c_{basal}=-\frac{\left(k_{\mathrm{off},\mathrm{GiGTP}}+k_{RGS}\right)}{k_{\mathrm{on},\mathrm{GiGTP}}}\frac{\chi }{{\left[AC\right]}_{tot}},$$

$$\chi =\left\{ \begin{array}{ll} 1 & \left(standard\;\mathrm{non}‐\mathrm{competitive}\;\mathrm{model}\right)\\ 1+G_{olf} & \left(\mathrm{competitive}\;\mathrm{model}\right) \end{array}\right.,$$

where *k*_*RGS*_ = *k*_cat,hyd,Gi_/*K*_m,hyd,Gi_·[*RGS*]_tot_, *k*_*DA*,basal_ = *k*_cat,exch_ [*DA*]_basal_ / ([*DA*]_basal_ + *K*_d,DA_), and *G*_olf_ = [*G*_olf_]_buff_/*K*_d,Golf_ (see **S1 and S2 Tables and section B in S1 Appendix**). *AC*_dip_ was also obtained by replacing *k*_*DA*,basal_ with *k*_*DA*,dip_. The analytical *AC*_basal_ and *AC*_dip_ were both well fitted with the simulated *AC*_basal_ and *AC*_dip_, respectively (**Figs 3B, 3C, 3D and S4B**; blue and light-blue dotted lines), and the analytical *AC*_basal_ and *AC*_dip_ were the functions of [*RGS*]_tot_, [*D2R*]_tot_, and [*AC*]_tot_, but not the function of [*G*_i_]_tot_. This is because the constraint (a) simplified the G_i_-dependent *V*_1_ (Eqs (5), (S1), and (S5), **section A in S1 Appendix**) into a G_i_-independent form (Eqs (S43) and (S44), **section A in S1 Appendix**). The constraint (a), *K*_m,exch,Gi_ << [*G*_i_:*G*_βγ_], was invalid in the small range of [*G*_i_]_tot_; thus, the analytical and simulated *AC*_basal_ were mismatched under [*G*_i_]_tot_ < ~0.04 μM (**Fig 3B**, second right).

Eq (24) well described the simulated *AC*_basal_ and *AC*_dip_. However, even with the constraints (a–d), Eq (24) was still too complicated to provide an intuitive understanding. We thus further simplified Eq (13) by considering its asymptotic functions under *k*_*RGS*_ << *k*_off,GiGDP_, *k*_off,GiGTP,_, i.e.,

$${\left[D2R\right]}_{\mathrm{tot}}=\frac{1-{AC}_{\mathrm{basal}}}{k_{DA,basal}}\{{\left[AC\right]}_{tot}+K_{d,\mathrm{GiGTP}}\frac{\chi }{{AC}_{\mathrm{basal}}}\}k_{RGS},$$ (14)

and under *k*_*RGS*_ >> *k*_off,GiGDP_, *k*_off,GiGTP,_, i.e.,

$${\left[D2R\right]}_{\mathrm{tot}}=\frac{1-{AC}_{\mathrm{basal}}}{k_{DA,basal}}k_{\mathrm{off},\mathrm{GiGDP}}\{{\left[AC\right]}_{tot}+\frac{k_{RGS}}{k_{\mathrm{on},\mathrm{GiGTP}}}\frac{\chi }{{AC}_{\mathrm{basal}}}\}.$$ (15)

**Fig 5:**
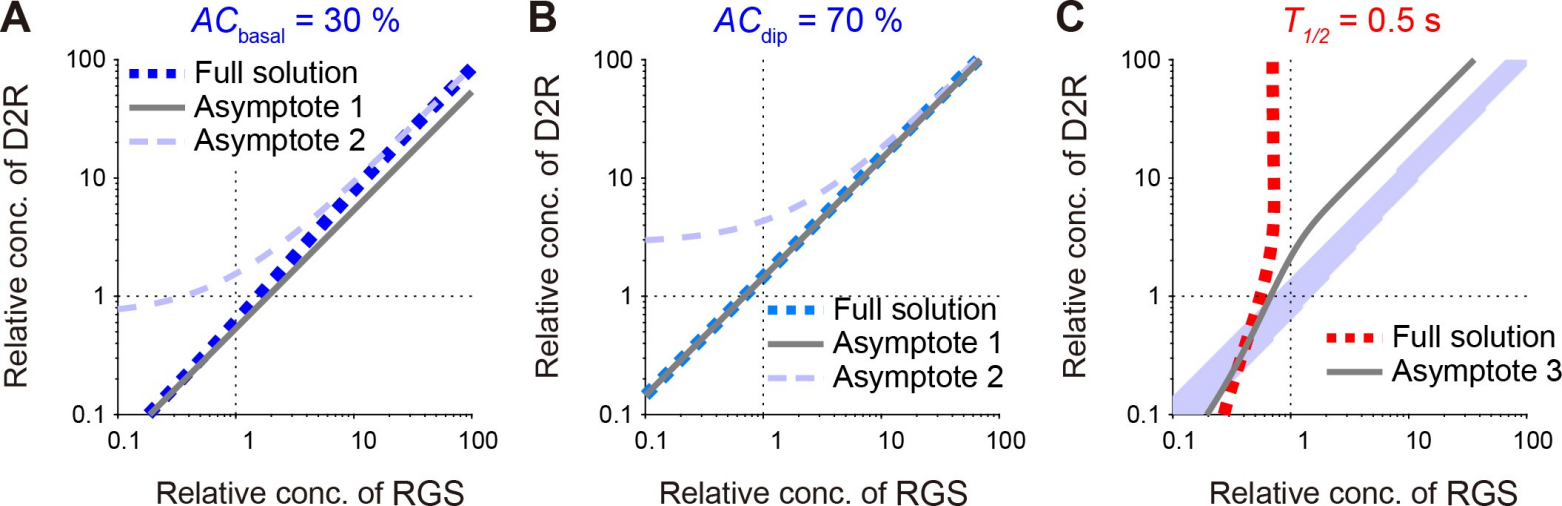
DA-dip detectable regions are characterized by asymptotic functions. (A, B) Analytical isolines of *AC*_basal_ and *AC*_dip_ (*AC*_basal_ = 30%, blue dotted line; *AC*_dip_ = 70%, light-blue dotted lines) are characterized by a transition between two linear functions, Eq (14) and Eq (15) (gray and light-blue dashed lines, respectively). (C) Analytical iso-*T*_1/2_ line (*T*_1/2_ = 0.5 s, red dotted line) is approximated by the function Eq (19) (gray dashed line) if it is located within *AC*_basal_ < 30% and *AC*_dip_ > 70% (blue shaded area).

Here, *k*_*RGS*_ << *k*_off,GiGDP_, *k*_off,GiGTP_ denotes the situation that G_i_-GTP hydrolysis is much slower than the G_i_-dissociation rate from AC, and *k*_*RGS*_ >> *k*_off,GiGDP_, *k*_off,GiGTP_ denotes the reversed situation. Eqs (14) and (15) denote the isolines of *AC*_basal_, i.e., [*D2R*]_*tot*_ = *f*([*RGS*]_tot_; *AC*_basal_), and they are both linear functions of [*RGS*]_tot_ (∝ *k*_*RGS*_); thus, *AC*_basal_ can be characterized by a transition between the two linear functions (**Fig 5A**). The gradients and D2R-intercept depend on *AC*_basal_, [*DA*]_basal_, [*AC*]_tot_, and kinetic constants. One of the asymptotic functions, Eq (15), appeared to be curved in the logarithmic space (**Fig 5A**, light-blue dashed line), but it is due to the D2R-intercept, and indeed linear in the linear space. The other asymptotic function (Eq (14); **Fig 5A**) appeared to be linear, because it has no D2R- or RGS-intercepts. *AC*_dip_ was also derived where *k*_*DA*,basal_ was replaced with *k*_*DA*,dip_, and showed the same characteristics (**Fig 5B**).

We next considered the half maximal time of *AC*_primed_(*t*), *T*_1/2_, where *AC*_primed_(*t* = *T*_1/2_) = (*AC*_basal_ + *AC*_dip_) / 2 after a sudden decrease of [*DA*] at *t* = 0 s (Eq (5); **Fig 3A**). Unfortunately, the dynamics of *AC*_primed_(*t*) was governed by exponentials of exponential functions, which has a complicated form (gamma functions) in the analytical solution. To avoid it, we further introduced three additional constraints to the D2 model:

(e) [*AC*_i_^site^]·[*G*_i_*-GTP*]/[*AC*_i_^site^:*G*_i_*-GTP*] = *K*_d,GiGTP_,
(f) [*AC*_i_^site^]·[*G*_i_*-GDP*]/[ *AC*_i_^site^:*G*_i_*-GDP*] = *K*_d,GiGDP_,
(g) [*AC*_olf_^site^]·[*G*_olf_*-GTP*]/[*AC*_i_^site^:*G*_olf_] = *K*_d,Golf_ (only for the competitive model),

where *K*_d,GiGTP_ = *k*_off,GiGTP_ /*k*_on,GiGTP,_, *K*_d,GiGDP_ = *k*_off,GiGDP_ /*k*_on,GiGDP_, and *K*_d,Golf_ = *k*_off,Golf_ /*k*_on,Golf_. The constraints (e-g) assume that the AC-G_i_ and AC-G_olf_ bindings rapidly reach equilibrium, and the dynamics of *AC*_primed_(*t*) is governed by G_i_-GTP hydrolysis. This operation is called the rapid equilibrium assumption in enzymology. We denoted *AC*_primed_ and *T*_1/2_ under the constraints (e-g) as ${\hat{AC}}_{\mathrm{primed}}$ and ${\hat{T}}_{1/2}$, respectively, and ${\hat{T}}_{1/2}$ was obtained as:

$${\hat{T}}_{1/2}=\frac{1}{k_{RGS}}ln\{\frac{k_{DA,basal}-k_{DA,dip}}{(1-{\hat{AC}}_{1/2})\{{\left[AC\right]}_{tot}+K_{d,\mathrm{GiGTP}}\frac{\chi }{{\hat{AC}}_{1/2}}\}\frac{k_{RGS}}{{\left[D2R\right]}_{\mathrm{tot}}}-k_{DA,dip}}\},$$ (16)

where ${\hat{AC}}_{1/2}=\left({\hat{AC}}_{\mathrm{basal}}+{\hat{AC}}_{\mathrm{dip}}\right)/2$, and ${\hat{AC}}_{basal}$ and ${\hat{AC}}_{dip}$ were given by:

$${\hat{AC}}_{basal}=\frac{-b_{basal}+\sqrt{b_{basal}^{2}-4c_{basal}}}{2},$$ (17)

$$b_{basal}=\frac{K_{d,\mathrm{GiGTP}}}{{\left[AC\right]}_{tot}}\chi +\frac{{\left[D2R\right]}_{\mathrm{tot}}}{{\left[AC\right]}_{tot}}\frac{k_{DA,basal}}{k_{RGS}}-1,$$

$$c_{basal}=-\frac{K_{d,\mathrm{GiGTP}}}{{\left[AC\right]}_{tot}}\chi ,$$

$${\hat{AC}}_{dip}=\frac{-b_{dip}-\sqrt{b_{dip}^{2}-4c_{dip}}}{2},$$ (18)

$$b_{dip}=\frac{K_{d,\mathrm{GiGTP}}}{{\left[AC\right]}_{tot}}\chi +\frac{{\left[D2R\right]}_{\mathrm{tot}}}{{\left[AC\right]}_{tot}}\frac{k_{DA,dip}}{k_{RGS}}-1,$$

$$c_{dip}=-\frac{K_{d,\mathrm{GiGTP}}}{{\left[AC\right]}_{tot}}\chi .$$

The derivations of Eqs (16), (17), and (18) are described in **section C in S1 Appendix**. The analytical ${\hat{T}}_{1/2}$ was well fitted with the simulated *T*_1/2_ (red dotted line, **Figs 4B, 4C, 4D and S4D**), showing the validity of the analytical formulation. The constraints (e, f) are not valid if the hydrolysis rate of G_i_-GTP, *k*_*RGS*_, is much larger than the rate of AC-G_i_ unbinding, and this invalidity appeared in the high [*RGS*]_tot_ (**Fig 4B**, second left). Similarly, the constraint (g) is not valid if *k*_*RGS*_ is much larger than the rate of AC-G_olf_ unbinding, as it also appeared (**S6B Fig**, right).

We finally simplified Eq (27) by considering if ${\hat{AC}}_{1/2}\to 50\%$:

$${\left[D2R\right]}_{tot}=\frac{k_{RGS}}{2}\frac{{\left[AC\right]}_{tot}+2\chi K_{d,\mathrm{GiGTP}}}{(k_{DA,basal}-k_{DA,\mathrm{dip}})\exp(-k_{RGS}∙{\hat{T}}_{1/2})+k_{DA,dip}}.$$ (19)

Here, ${\hat{AC}}_{1/2}\to 50\%$ represents an ideal situation, i.e., *AC*_basal_ = 0% and *AC*_dip_ = 100%, or *AC*_basal_ + *AC*_dip_ = 100%. The asymptotic ${\hat{T}}_{1/2}$ (Fig 4C, gray dashed line) was almost the same as the analytical one (**Fig 5C**, red dotted line) if *AC*_basal_ < 30% and *AC*_dip_ > 70% (**Fig 5C**, blue shaded area). We thus obtained an analytically closed form of the isoline of *T*_1/2_ in the DA-dip detectable region. In Eq (19), ${\hat{T}}_{1/2}$ is the decreasing function of [*RGS*]_tot_ (∝ *k*_*RGS*_). Thus, [*RGS*]_*tot*_ must be higher than the isoline for the shorter DA-dip detection.

In summary, we derived the analytical forms of *AC*_basal,_ *AC*_dip,_ and *T*_1/2_. *AC*_basal_ and *AC*_dip_ were both characterized by the transition between two asymptotic linear functions (Eqs (14) and (15); **Fig 5A and 5B**), and *T*_1/2_ also had an approximate closed form (Eqs (16)–(18); **Fig 4B−4E**). The boundaries of *AC*_basal_ and *AC*_dip_ were linear regardless of the model parameters as far as they satisfied the constraints (a–d), and such a linear relationship was also seen in developing mice (**Fig 3E**) [28].

### AC-concentration dependence

The DA-dip detectability also depended on [*AC*]_tot_ (**Figs 3B and 4B**, center). We thus examined two-way relationships between [*AC*]_tot_ and [*D2R*]_tot_ (**S7C and S7F Fig**) as well as between [*AC*]_tot_ and [*RGS*]_tot_ (**S7D and S7G Fig**). The DA-dip detectable region in [*AC*]_tot_ showed a positive relationship with that in [*D2R*]_tot_ (**S7C Fig**), and a negative relationship with that in [*RGS*]_tot_ (**S7D and S7G Fig**). These [*AC*]_tot_ dependences originated from the sequestration of G_i_-GTP for the inhibition of AC. If G_i_-GTP was set not to be sequestrated by AC, i.e., *V*_4_, …, *V*_7_ were removed only from Eq (S3) (**S7A and S7H Fig**), the [*AC*]_tot_ dependences were completely eliminated (**S7J, S7K and S7N Fig**), while the relationship between [*RGS*]_tot_ and [*D2R*]_tot_ was still preserved (**S7I and S7L Fig**). Furthermore, the lower [*AC*]_tot_ was, the lower [*AC*]_tot_ dependences appeared (**S7C, S7D and S7G Fig**) because the smaller amount of G_i_-GTP was sequestered by AC.

### Dynamics of *AC*_primed_ under psychiatric/movement disorders

**Fig 6:**
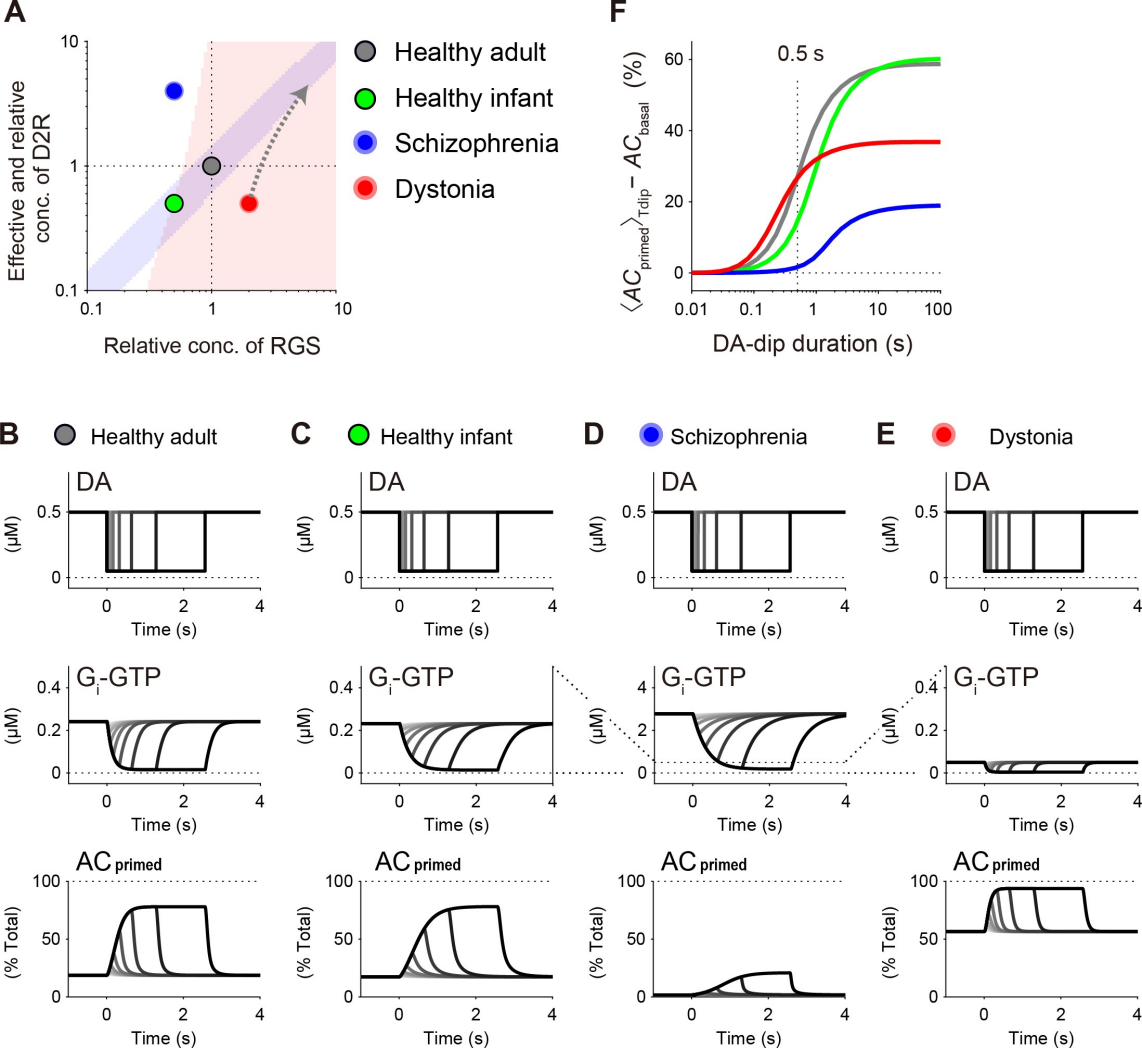
DA dip is undetectable under the pathologic imbalance between D2R and RGS. (A) Levels of D2R and RGS under healthy and pathologic conditions. Healthy infant mice show lower levels of D2R and RGS (green circle) than healthy adult mice do (standard model; gray circle, see Fig 2D) [28]. Schizophrenia patients are known to show higher and lower activities of D2R and RGS, respectively (blue circle) [29,31,32], and a mouse model of DYT1 dystonia shows the opposite changes in their levels (red circle, Fig 2D) [28]. Gray arrow denotes the hypothetical trajectory of changes in RGS and D2R levels if the levels of RGS and D2R are increased from the dystonic levels by the same ratio as in the increasing ratio under the development of healthy mice, i.e., ([*D2R*], [*RGS*]) = *μ* × ([*D2R*]_tot_, [*RGS*]_tot_) + ([*D2R*]_Dystonia_, [*RGS*]_Dystonia_) where [*D2R*]_Dystonia_ and [*RGS*]_Dystonia_ are the dystonic levels of D2R and RGS, and *μ* is the constant (> 0; see Discussion). (B–E) Dynamics of [*G*_*i*_*-GTP*] and *AC*_primed_ against DA dips with a variety of durations (0.01 s, 0.02 s, …, 2.56 s). (F) Summary of the DA-dip duration dependences quantified using <*AC*_primed_>_Tdip_− *AC*_basal_ (see Methods).

Healthy mice show an age-dependent simultaneous increase in the levels of striatal D2R and RGS9–2 during postnatal development (**Fig 3E**) [28,50,51]. This D2R–RGS balance is known to be disrupted in psychiatric/movement disorders. Schizophrenia patients often show a supersensitivity of D2R and/or an increase in DA [29,60], and mice in a corresponding mouse model show the decrease in the gross expression level of RGS9–2 in the striatum [31,32]. The altered levels of D2R and RGS disturb the intracellular signaling of SPNs [61], and such abnormal signaling and subsequent striatal dysfunction is expected to cause these psychological symptoms [62]. By contrast, a mouse model of DYT1 dystonia shows the decrease and increase in D2R and RGS9–2, respectively, in the co-existent fraction of D2R and RGS9–2 (DRM; **Fig 3E**, red points) [28], and the increased GTP hydrolysis may be related to involuntary movements. Thus, we explored the DA-dip detectability under such D2R–RGS imbalances (**Fig 6**).

We first confirmed that the D2 model with the standard set of parameters (healthy-adult model; **Fig 6A**, gray circle) successfully detected short DA dips (~0.5 s; **Fig 6B**). The *AC*_primed_ was sufficiently low under the steady-state G_i_-GTP (**Fig 6B**; *AC*_basal_ = 19% where [*G*_*i*_*-GTP*]_basal_ = 0.24 μM), whereas the *AC*_primed_ was sufficiently and rapidly increased during DA dips (**Fig 6B**; *AC*_dip_ = 78% where [*G*_*i*_*-GTP*]_dip_ = 0.016 μM). Then we set a condition for healthy infant that had 0.5 × [*D2R*]_tot_ and 0.5 × [*RGS*]_tot_ (**Fig 6A**, green circle), and found the similar response of *AC*_primed_ to the DA dips (**Fig 6C**; *AC*_basal_ = 17% where [*G*_*i*_*-GTP*]_basal_ = 0.23 μM; *AC*_dip_ = 78% where [*G*_*i*_*-GTP*]_dip_ = 0.014 μM), although it showed lower sensitivity to the shorter dips. On the contrary, a schizophrenia model that had 4.0 × [*D2R*]_tot_ and 0.5 × [*RGS*]_tot_ (**Fig 6A**, blue circle) did not detect the DA dips because of the excessive amount of G_i_-GTP. It caused the excessively low *AC*_primed_ at the basal state and its lower and slower increase (**Fig 6D**; *AC*_basal_ = 2% where [*G*_*i*_*-GTP*]_basal_ = 2.8 μM; *AC*_dip_ = 21% where [*G*_*i*_*-GTP*]_dip_ = 0.19 μM). Similarly, a dystonia model that had 0.5 × [*D2R*]_*tot*_ and 2.0 × [*RGS*]_tot_ (**Fig 6A**, red circle) showed high *AC*_basal_ and weak responses to the DA dips (Fig 6E; *AC*_basal_ = 57% where [*G*_*i*_*-GTP*]_basal_ = 0.050 μM; *AC*_dip_ = 94% where [*G*_*i*_*-GTP*]_dip_ = 0.0043 μM). In summary, the D2 models for healthy adult and healthy infant responded to DA dips with sufficiently large dynamic ranges (**Fig 6F**, gray and green lines, respectively), although the healthy-infant model was less sensitive to shorter DA dips. The schizophrenia model showed the slower and smaller responses of AC against DA dips (**Fig 6F**, blue line), and the dystonia model showed the rapid but small response (**Fig 6F**, red line).

Apparently, their DA-dip detectability was disrupted by the excessive or insufficient levels of G_i_-GTP at the basal states (**Fig 6B−6E**, center), and they can be understood using a schematic picture (**S8A Fig**). At the basal state, DA-dependent D2R activity works like a faucet (tap) that provides G_i_-GTP with a constant rate (**S8A Fig**, left). The production rate, *V*_1_, is almost independent of [*G*_i_:*G*_βγ_] because [*G*_i_]_tot_ (~9 μM) is much higher than *K*_m,exch,Gi_ (~0.01 μM, **S2 Table**; constraint (a)). The produced G_i_-GTP is drained through RGS, where the draining rate, *V*_2_ + *V*_8_, is proportional to [*G*_*i*_*-GTP*] + [*AC*:*G*_*i*_*-GTP*] (Eqs (S40) and (S41), **section A in S1 Appendix**) because the GTP hydrolysis is unsaturated (constraint (b)). The difference in these rates makes a pool of G_i_-GTP (**S8A Fig**, left). In the healthy-adult model, the pooled G_i_-GTP almost completely inhibits AC, which is represented by a ball (**S8A Fig**). Here, the G_i_-soaked part of AC is inhibited, and the G_i_-free part is disinhibited (**S8A Fig**, inset). During the DA-dip, the production of G_i_-GTP almost stops (**S8A Fig**, right), and the level of G_i_-GTP is decreased for the activation of AC for LTP and/or the change of neuronal excitability. In the schizophrenia model, the larger [*D2R*]_tot_ makes the production rate of G_i_-GTP, *V*_1_, much higher than its draining rate, *V*_2_ + *V*_8_ (S8D Fig), leading to the complete inhibition of AC. In the dystonia model, the production rate is conversely lower than the draining rate (**S8E Fig**). These imbalances caused the decrease the levels of DA-dip detection (**Fig 6F**).

### Time windows for *AC*_active_ under psychiatric/movement disorders

**Fig 7:**
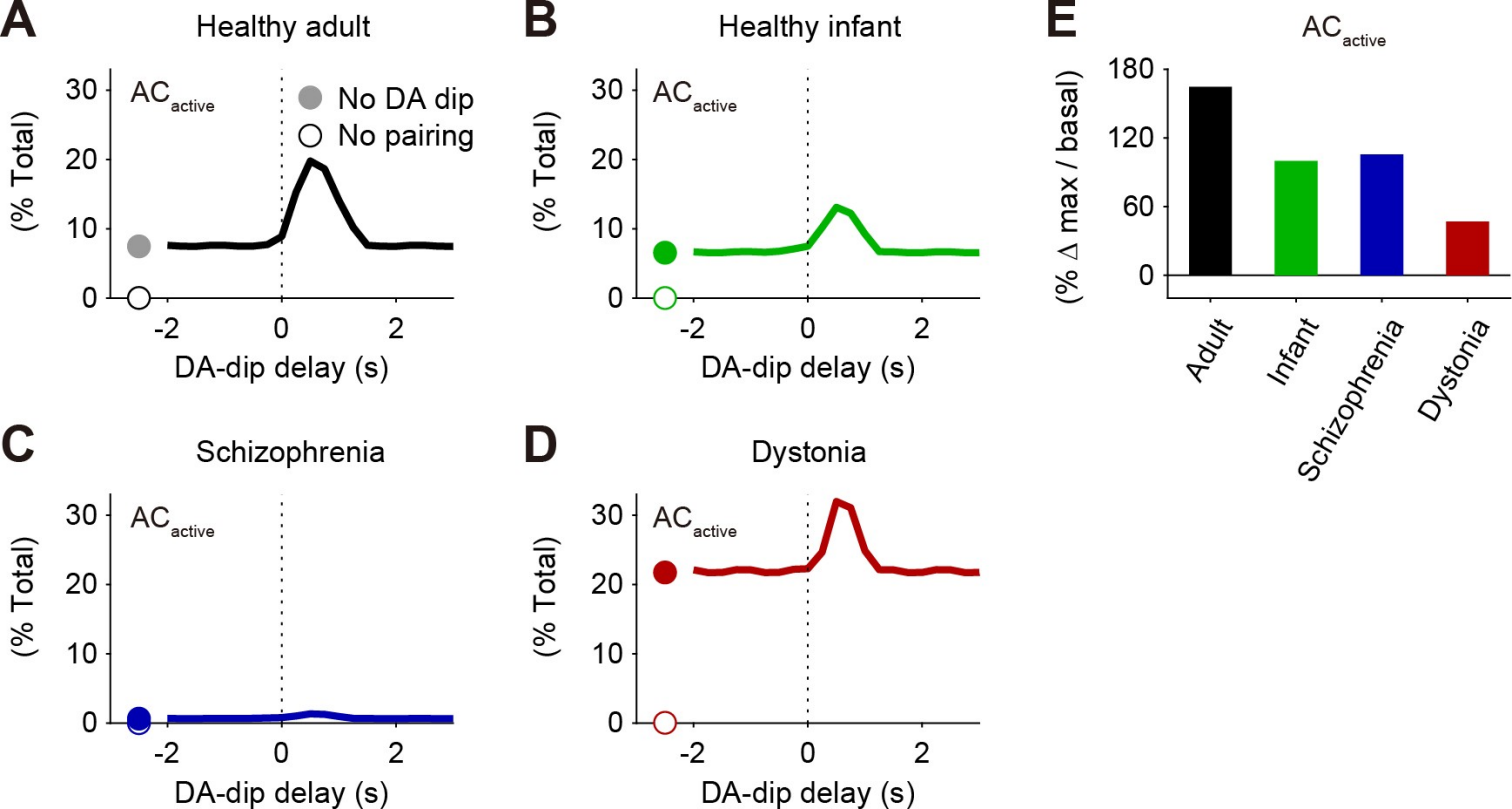
Decreased efficacy of the coincidence detection in pathologic/movement disorders. (A-D) Time windows for DA-dip delay against pre–post pairing on the activity of AC1, [*AC*_active_], in the models of healthy adult (A), healthy infant (B), schizophrenia (C), and DYT1 dystonia (D). For each DA-di delay, a 0.5-s DA dip is paired with 1-s pre-post pairing. Obtained maximal [*AC*_active_] are normalized by the total concentrations of AC1, [*AC*]_tot_. (E) Maximal amplitudes of *AC*_active_ normalized by the basal level.

Finally, we examined the timing detection for AC1 in D2 SPNs in the healthy adult, healthy infant, schizophrenia, and DYT1 dystonia (**Fig 7**). Compared to the healthy-adult model (the standard set of parameters, **S1 and S2 Tables** and **Fig 7A**), the healthy-infant model showed the smaller level of DA-dip detection in *AC*_acitve_ because the DA dips had a short period of 0.5 s (**Fig 7B**). The schizophrenia model did not show any increase in *AC*_active_ (**Fig 7C**), while the dystonia model detected the DA-dip, but the timing-independent component of *AC*_acitve_ was high (**Fig 7D**). Because of the high timing-independent *AC*_active_, the peak-to-basal ratio of *AC*_active_ in the DYT1 dystonia became the lowest (**Fig 7E**), indicating the small signal-to-noise ratio even if the activity of the downstream signaling can adapt to the high timing-independent levels of *AC*_active_.

Following a previous study [24], we also simulated AC5 coincidence detection between a phasic burst of adenosine (duration: 1 s) and a DA dip/burst (duration: 2 s; **S9A−S9C Fig**). The time windows for the delay of DA dip/burst were similar to those in AC1 (**S9D−S9G Fig**), and the dystonia model rather showed DA-burst detection, because the longer DA burst completely inhibited the increase in *AC*_active_ due to the adenosine burst (**S9G Fig**, thin lines).

## Discussion

Here, we showed that a D2R–RGS balance was required for the detection of DA dips in D2 SPNs, where the DA-dip detection is important for the LTP and/or change of neuronal excitability. High-level RGS was further required for the detection of short (~0.5 s) DA dips. These requirements were satisfied in healthy development but disrupted in our models of schizophrenia and DYT1 dystonia.

The D2 model highlights the importance of RGS. In particular, a type of RGS, RGS9–2, is known to be specifically and abundantly expressed in the striatum [27,34,63]. The expression of RGS9–2 is co-regulated with that of D2R [28,64], and RGS9–2 itself is associated and colocalized with D2R [65]. The present study provides a reason why RGS9–2 is so tightly coupled with D2R. It is necessary for the reliable detection of short DA dips. The abundance of RGS9 is also seen in the retinal phototransduction processes [66], and the rapid hydrolysis of transduction via RGS9 is vital for the rapid visual processing both in rods and cones [67]. Although G-protein signaling is often discussed from its chronic aspects [68], it can also serve for sub-second information processing. In addition, a variety of RGS subtypes are expressed in the brain in a region-specific manner [63,69]. RGS8 and RGS10 are characteristically expressed in cerebellar Purkinje cells as well as in the stratum granulosum of the hippocampus, respectively [63]. They may also play roles for region-specific information processing.

Nair et al. have already demonstrated DA-dip detection by AC5 in the model of D2 SPNs [19,24], in which a set of model parameters were determined based on observed molecular activities. The D2 signaling model has shown the requirement of a longer DA dip for the AC5 response (c.f., **Fig 6**) [19]. It has also shown the requirement of higher concentration of basal DA for the AC5 response, which was shown as the region of *AC*_basal_ < 30% in the D2 model (**S5A Fig**, left). Further, the D2 signaling model has shown the requirement of lower concentration of DA during a DA dip [24], as shown in *AC*_dip_ > 70% of the D2 model (**S5B Fig**, left). However, the D2 signaling model does not incorporate RGS, but the GTP hydrolysis of G_i_ is assumed to occur with a constant rate. Here, we extensively examined the concentration dependence of five target molecules (**Fig 3B**), and found the requirement of the D2R–RGS balance for the DA dip detection, together with the analytical solutions that prove the universality of the balance requirement. Nair et al. have also demonstrated a possible coincidence detection between DA durst and acetylcholine (ACh) dip, the latter of which leads to the deactivation of a G_i_-coupled receptor (muscarinic M4 ACh receptor; M4R) [24]. RGS should also play a critical role in such other types of G_i_ inactivation-driven events.

In the present study, we derived analytical solutions for the DA-dip detection. They were valid in a wide range of parameters, as far as the D2 model satisfied the constraints (a–g). In particular, the constraints (a, b) were related to famous simplifications of the MM formulation. The constraint (a) enabled the catalyst-saturated approximation, and the constraint (b) enabled the first-order rate approximation. They contributed not only to the simplification but also to stable DA-dip detection. For example, if the G_i_-GTP hydrolysis were conversely saturated, the GTP hydrolysis rate, *V*_2_ + *V*_8_ (Eq (S65) in **section C in S1 Appendix**), would be independent of [*GTP*] (= [*G*_i_-*GTP*] + [*AC*:*G*_i_-*GTP*]), but only dependent on [*RGS*]. Then, [*GTP*] would become an increasing or decreasing function depending only on [*RGS*], and G_i_ took only one of two stable states, i.e., fully hydrolyzed or non-hydrolyzed states. *AC*_primed_ could not show a rapid increase from the fully non-hydrolyzed state of G_i_ (i.e., 100% G_i_-GTP), nor detect a short DA-dip. The kinetics constants and molecular concentrations seem to be arranged for effective DA-dip detection, which may be a design principle in the biological system, as often quoted in the field of systems biology [70].

In schizophrenia, the balance between D2R and RGS activities is considered to be biased toward D2R (**Fig 6A**), because D2R blockers works as antipsychotic drugs for schizophrenia [52,53], and schizophrenia shows show a supersensitivity of D2R and/or an increase in striatal DA [29,60] as well as a decrease in striatal RGS9–2 [31,32]. Our D2 model predicts that the excessive activity of postsynaptic D2R completely inhibits AC, disabling the detection of any DA dips as well as subsequent LTP (**Fig 7C**). Correspondingly, chronic upregulation of D2R is known to result in the rewiring of connections first from the striatum to the globus pallidus (GPe) [71] and next within the frontal cortex [61,62]. Such rewiring may lead to the persistent symptoms of schizophrenia. D2R blockers are widely used for the medication of schizophrenia [52], but the present study predicts that the excess blockage of D2R again disables DA-dip detection. Indeed, excessive treatment with the D2R blockers is known to induce extrapyramidal side effects including tardive dyskinesia [31,72], a movement disorder that shares the similar symptoms with DYT1 dystonia [73] whose mouse model shows the D2R–RGS imbalance toward RGS (Fig 2D).

DYT1 dystonia is a hyperkinetic movement disorder, which originates from a Δgag mutation in the *TOR1A* gene. It decreases the gross expression levels of both D2R and RGS9–2 [73,74], but the level of RGS9-2 is selectively increased in the DRM where D2R is located, and the D2R–RGS balance is biased toward RGS. In our D2 model, the excess RGS first decreases the level of G_i_-GTP, and then increases the AC activity for cAMP production. Excess cAMP is expected to be a cause of abnormal LTP [33,54,75,76]. Such synaptic abnormality leads to the abnormal neuronal activities in GPe [77] and thalamus [78], and they may cause muscle contractions and irregular tremors [79]. Notwithstanding with these reports, DA drugs do not provide clinical benefit [80], and the viral overexpression of RGS9–2 rather restores normal neuronal electrophysiology [28]. These inconsistent observations are reconciled in the scheme of the D2 model as follows. First, DA drugs should not work because they cannot accurately control the level of D2R activity to counteract with RGS (**Fig 6A**). Next, the overexpression of RGS9–2 also rescues the decreased expression level of D2R [28], and the increased levels of RGS and D2R can be balanced within the DA-dip detectable region. Indeed, this rebalancing would be realized if the levels of RGS and D2R were increased from the dystonic levels by the same ratio as in normal development (**Fig 6A**, gray arrow). The overexpression of RGS9–2 thus can restore normal neuronal activity [28]. Together, the D2 model explains many aspects of DYT1 dystonia in the space of D2R and RGS, including the aspects of healthy development and schizophrenia.

The balance requirement between D2R and RGS itself has been recognized in the field of psychiatry [32,53], and here we re-formalized it as requirements for the DA-dip detection for LTP in D2 SPNs. Note that pairs of [*D2R*]_tot_ and [*RGS*]_tot_ for the healthy and pathologic conditions were determined only for exemplifying purpose (Fig 6A), and their absolute values are case-dependent even in mice experiments (see Methods). In particular, schizophrenia is caused by both environmental and genetic factors, and many combinations of the genes affect schizophrenia [81]. Thus, the pairs of effective [*D2R*]_tot_ and [*RGS*]_tot_ are expected to span a wide range depending on the patients, even if it is caused by excessive D2R activity.

The other major movement disorder, L-DOPA induced dyskinesia (LID), could also be related to the D2R–RGS imbalance. The depletion of DA is associated with Parkinson’s disease, and the DA depletion is restored by administration of a DA precursor, L-DOPA [82]. The L-DOPA medication leads to varying levels of basal DA, which is followed by the co-adaptation of D2R and RGS9–2 [28,65]. The incompleteness of the adaptation may be a cause of LID. Indeed, RGS9–2 knock-out (KO) develops LID [65], and the overexpression of RGS9–2 diminishes its involuntary movements [35]. LID also shows abnormal LTP [13,83]. However, other recent studies have shown that LID are primarily linked to the abnormal activity of D1 SPNs [84–86], and the dissection of mixed effects is necessary to understand this currently untreatable disease.

In the present study, we showed the D2R–RGS balance as a shared requirement of both AC1 and AC5. As it is known, AC5 constitutes ~80% of the total cAMP levels in SPNs [17], and contributes to synaptic plasticity [21]. Considering its abundance, AC5 should also perform the coincidence detection in some forms of synaptic plasticity as well as the plastic changes in neuronal excitability [22,23]. The AC5 coincidence detection is predicted to occur between G_olf_ and G_i_ (**S8 Fig**) [20,24]. However, in our primary target experiment, an AC1-specific inhibitor fully suppressed AC-dependent cAMP signaling, and DA signal alone did not activate the D1R–G_olf_–AC–cAMP signaling, but further pre–post pairing (presumably Ca^2+^) was required for the cAMP signal [5]. These observations support the role of Ca^2+^-sensitive AC1, and further, the AC1-based D2 LTP model successfully predicted the narrow time window (~2 s) (**Fig 1A**) [10]. Such Ca^2+^-sensitive ACs have been known as a coincidence detector for the classical conditioning in Aplysia and Drosophila [15,87–89]; thus, the Ca^2+^ requirement may be evolutionarily conserved [90]. The cause of missing AC5 signal in the target experiment is unknown [5], while an AC5-based model predicts much longer time window (~10 s) [91]. In general, the expression of AC1 in the striatum is known to be decreased with the development, while the Ca^2+^-CaM-dependent activity of AC persists [92].

AC1 and AC5 are both expressed in the cortex and hippocampus [16]. However, these regions only show weak DA signal because of the small numbers of DA fibers [93,94], and the DA signal likely plays more modulatory roles with longer time constants (~10 min) [6,95]. Rather, in these brain regions, adenosine can show persistent and transient signals with a time range of ~5 s [96,97]. Adenosine A1 receptors (A1AR) then produce G_i/o_-GTP, while A2AR produce G_s/olf_-GTP [98,99]. Thus, adenosine dip may lead to the reduction of G_i/o_-GTP via A1AR, similarly to the DA-dip signal through D2R. In the striatum, cholinergic interneurons also show tonic and ~0.1-s phasic activities [100], and the ACh release stimulates postsynaptic M4R in D1 SPNs for G_i_-GTP [20,24,101]. The produced G_i_-GTP may also activate AC, similar to DA-dip signal. Further, DA and ACh signals are known to interact with each other. The activity of DA fibers modulates the activity of cholinergic interneurons via co-released glutamate [102–104], and ACh conversely stimulates the DA fibers [105]. The ACh–DA interaction may enhance the coincidence detection *in vivo* [106].

An important limitation of the current D2 model is the mass assumption. In addition to well-known dimerization of D2R and A2AR [107], recent experiments have suggested that AC5, G_i_, and G_olf_ also participate in the formation of a macromolecular complex that accelerated reactions among the constituents [108,109]. Such a macromolecular complex no longer obeys the mass assumption; thus, their reactions cannot be formulated by Eqs (1) and (2), but should be described in a more mechanistic manner. In the macromolecular complex, the DA signal would be more rapidly and directly transferred to the activity of AC, the short DA-dip might be more easily detected by AC. This is an attractive scenario and an important direction for future studies. Nevertheless, the D2 model should first be built based on the mass assumption, especially before the validation of its dominance in the physiological condition. As an example, the D2R:G_i_:G_βγ_ pre-coupled complex has been discovered and examined [110–112], but it seems not to be dominant in the physiological condition, from the viewpoint of the affinity of D2R for DA (*K*_d,DA_ in pre-coupled complex, 25 nM; *K*_d,DA_ in the physiological condition, ~10 μM) [1,19,43,113,114]. In addition, even if such a macromolecular complex is found to work dominantly, the current D2 model will serve as a good reference.

The present study has addressed the DA-dip detection in D2 SPNs. Similarly, phasic DA bursts are known to trigger LTP in D1 SPNs [5]. Yet, do the similar requirements exist in D1 SPNs? In D1 SPNs, DA binds to D1R, and the DA-bound D1R produces G_olf_-GTP. However, it is known that the hydrolysis of G_olf_-GTP is not mediated by any of the RGS, but occurs in an autocatalytic manner [46]; thus, we cannot ask the same question about D1 SPNs. Instead, we can raise another important question: how can the phasic bursts of DA trigger LTP in D1 SPNs, despite the fact that the basal DA signal alone does not trigger the LTP [1]? This question implies the existence of an adaptation mechanism for basal DA signal. A possible candidate is a feedback loop that involves a specific phosphorylation of D1R [115]. The DA-bound D1R produces G_olf_-GTP, which activates AC. The activated AC produces cAMP and then the active form of PKA. The PKA in turn phosphorylates D1R, leading to a ~100-fold decrease in the activity of D1R, with a time constant of ~10 min [115]. Thus, the D1R–AC–PKA signaling constitutes a negative feedback loop in which D1R responds solely to the phasic DA signal. Simulation of such a feedback loop will address the stability of D1 SPNs against the fluctuation of basal DA signal. However, it requires a different model with a different level of abstraction. Thus, this topic should be addressed in other future studies.

## Supporting information

S1 Fig Schematic of the D2 model (standard non-competitive model). Arrows denote the first order reactions or enzymatic reactions, and their fluxes are denoted by *V*_1_,…, *V*_12_. (A) Reactions of the D2R–G_i_–AC part. The input DA (circled) first regulates a G_i_-protein cycle (top), then G_i_-AC binding cycle (bottom). G_i_-GTP/ G_i_-GDP binds to a specific site of AC (AC_i_^site^). (B) Under the scheme of non-competitive binding, G_olf_, G_i_, and Ca^2+^-CaM independently interact with their specific sites of AC (AC_olf_^site^, AC_i_^site^, and AC_CaM_^site^, respectively). Simultaneous binding of G_olf_ and Ca^2+^-CaM is required for the activity of AC1 (top), and the binding of G_olf_ alone leads to the activity of AC5 (bottom). The binding of G_i_ inhibits the activities of both AC1 and AC5. Reactions with dashed arrows are the same as the reactions *V*_4_,…, *V*_7_ indicated in panel A.
(TIF)

S2 Fig Schematic of the D2 model (Competitive model). Arrows denote the first order reactions or enzymatic reactions, and their fluxes are denoted by *V*_1_,…, *V*_20_. (A) Reactions of the D2R–G_i_–AC part. Same as S1A Fig, but the G_i_ binding to AC disables the binding of G_olf_ and Ca^2+^-CaM. (B) If AC is free from G_i_, the AC can interact with G_olf_ and Ca^2+^-CaM. Simultaneous binding of G_olf_ and Ca^2+^-CaM is required for the activity of AC1 (top), and the binding of G_olf_ alone sufficiently activates AC5 (bottom). Under the competitive binding, *AC*_primed_ corresponds to the levels of yellow-bordered states. Reactions with dashed arrows are the same as the reactions *V*_4_,…, *V*_7_ indicated in panel A.
(TIF)

S3 Fig Dynamics of molecular activities in response to a DA dip. Red arrows denote the onset of a 1-s DA dip. (A) Observed molecules. (B) Optogenetically-evoked dynamics of DA. (C–I) Subsequent molecular activities. (J) Square-drop signal as a representative of DA dip. (K-Q) Subsequent molecular activities.
(TIF)

S4 FigDA-dip detectable region in the competitive model.(A) *AC*_primed_ is observed under the stepwise decreasing signal of DA. (B) Concentration dependence of the steady state levels of *AC*_primed_, *AC*_basal_ and *AC*_dip_ in the competitive model. (C) G_olf_-dependence of *ΔAC*_activity_ in the non-competitive and competitive models (see Eqs (7) and (10)). ${\Delta AC}_{active}={{AC}_{active}|}_{[DA]={\left[DA\right]}_{dip}}{{-AC}_{active}|}_{[DA]={\left[DA\right]}_{basal}}$ where ${{AC}_{active}|}_{[DA]={\left[DA\right]}_{basal}}$ and ${{AC}_{active}|}_{[DA]={\left[DA\right]}_{dip}}$ denote the activities of AC5 under [*DA*]_basal_ and [*DA*]_dip_, respectively. The biphasic G_olf_ concentration dependence appears only in the competitive model, as shown in Bruce et al. (2019) [20]. (D) Parameter dependence of *T*_1/2_ in the competitive model.(TIF)

S5 Fig Requirement of D2R–RGS balance under various levels of [*DA*]_basal_ and [*DA*]_dip_. (A) [*DA*]_basal_ is set to be 0.125, 0.25, …, 4 μM (left), and the areas of *AC*_basal_ < 30% (top) and *T*_1/2_ < 0.5 s (bottom) are plotted in the space of [*RGS*]_tot_ and [*DA*]_tot_. Dotted lines denote the analytical solutions, and colored areas denote the simulation results. (B) [*DA*]_dip_ is set to be 0.0125, 0.025, …, 0.4 μM (left), and the areas of *AC*_dip_ > 70% (top) and *T*_1/2_ < 0.5 s (bottom) are plotted.
(TIF)

S6 Fig Reaction rate of G_olf_ affects *T*_1/2_ only in the competitive model. Here, the binding/unbinding reaction rate of G_olf_, *τ* = 1/(*k*_on,Golf_[*G*_olf_]_buff_+*k*_off,Golf_), is subjected to change, whereas *K*_d,Golf_ (= *k*_off,Golf_ / *k*_on,Golf_) and [*G*_olf_]_buff_ are kept constant. (A, B) *AC*_basal_, *AC*_dip_, and *T*_1/2_ in the standard non-competitive model (A), and competitive model (B). Green dotted line in panel B denotes *τ* = 0.5/log 2 (*T*_1/2_ = 0.5 s).
(TIF)

S7 Fig AC-concentration dependence is based on the sequestration of G_i_ by AC. (A–G) Areas of *AC*_basal_ < 30% (blue), *AC*_dip_ > 70% (light blue), and *T*_1/2_ < 0.5 s (pink) in the standard non-competitive model (A). These areas are plotted in the spaces of [*D2R*]_tot_ versus [*RGS*]_tot_ (B, E), [*D2R*]_tot_ versus [*AC*]_tot_ (C, F), and [*RGS*]_tot_ versus [*AC*]_tot_ (D, G). Dotted lines denote their analytical solutions. (H–N) Same as panels A–G, but the standard D2 model is modified so that AC sequesters G_i_, i.e., *V*_4_, *V*_5_, *V*_6_, and *V*_7_ are removed only from Eq (S3) but not from Eq (S12).
(TIF)

S8 Fig Schematic of the D2-model dynamics. (A) At the steady state (left), DA-bound D2R provides a constant flux of G_i_-GTP (*V*_1_; see **section A in S1 Appendix**), while the G_i_-GTP is drained through RGS with a speed (*V*_2_ + *V*_8_) that is proportional to the amount of G_i_-GTP. Difference in the fluxes generates a pool of G_i_-GTP that inhibits AC, which is represented by a sunk ball (AC). During the period of a DA dip (right), the supply of G_i_-GTP nearly completely stops, and the pooled G_i_-GTP is drained rapidly. AC is then disinhibited to be activated by G_olf_ and Ca^2+^-CaM. (B-E) The G_i_ inhibition of AC in the healthy adult, healthy infant, schizophrenia, and dystonia, all of which are described in **Fig 6A−6E**. (C) In the healthy-infant model, the influx and efflux of G_i_-GTP are both small, and the smaller efflux causes delayed disinhibition of AC during a DA dip. (D) In the schizophrenia model, hyperactive D2R provides a larger amount of G_i_-GTP, thus AC is fully inhibited and its disinhibition is delayed. (E) The larger efflux of G_i_-GTP in the dystonia model results in the chronic activity of AC.
(TIF)

S9 Fig AC5 performs the coincidence detection between G_olf_ and G_i_. A transient burst in adenosine (thus G_olf_) coincides with a DA dip/burst with a variety of delay times. (A) Schematic of AC5 signaling. (B) Adenosine burst as a square wave of G_olf_ (basal, 0.16 μM; burst, 0.8 μM; duration, 1 s). (C) Square-wave dip and burst of DA (basal, 0.5 μM; dip, 0.05 μM; burst, 2 μM; duration, 2 s). (D-G) Example traces of the AC5 activities (*AC*_active_; top), and DA-dip/burst delay dependence of the maximal *AC*_active_ (bottom) in the healthy-adult, healthy-infant, schizophrenia, and dystonia models. In the example traces, adenosine and DA signals were given during the periods indicated by blue and red bars, respectively. Similar simulation (adenosine versus transient DA dips) has been conducted by Nair et al. (2015) [24].
(TIF)

S1 Table Molecular concentrations. Densities of membrane molecules should have the unit of membrane area (/μm^2^), but not volume (μM). However, many of the referenced experiments/simulations have described them under homogenate conditions; therefore, we also adopted volume concentration for consistency. Note that *X*_area_ /μm^2^ ~ *X*_volume_ μM × 20, because a spherical spine with a radius *r*_spine_ ~ 0.1 μm has the number of surface molecules *X*_area_ × (4π*r*_spine_^2^), and the number of cytosolic molecules is *X*_volume_ × 10^−6^*N*_A_ × [4π*r*_spine_^3^/3 × 10^−15^] where *N*_A_ = 6.02 ×10^23^ (Avogadro constant).
(PDF)

S2 Table Reaction rate constants. (PDF)

S1 Appendix Model definition and analytical derivations. (A) Definition of the D2 model. (B) Derivation of analytical *AC*_basal_ and *AC*_dip_. (C) Derivation of analytical *T*_1/2_.
(PDF)
