# Supplementary figures and images for: The critical balance between dopamine D2 receptor and RGS for the sensitive detection of a transient decay in dopamine signal
Source: PLoS Comput Biol. 2021 Sep 30;17(9):e1009364. doi: 10.1371/journal.pcbi.1009364 (PMC8483376; doi:10.1371/journal.pcbi.1009364)

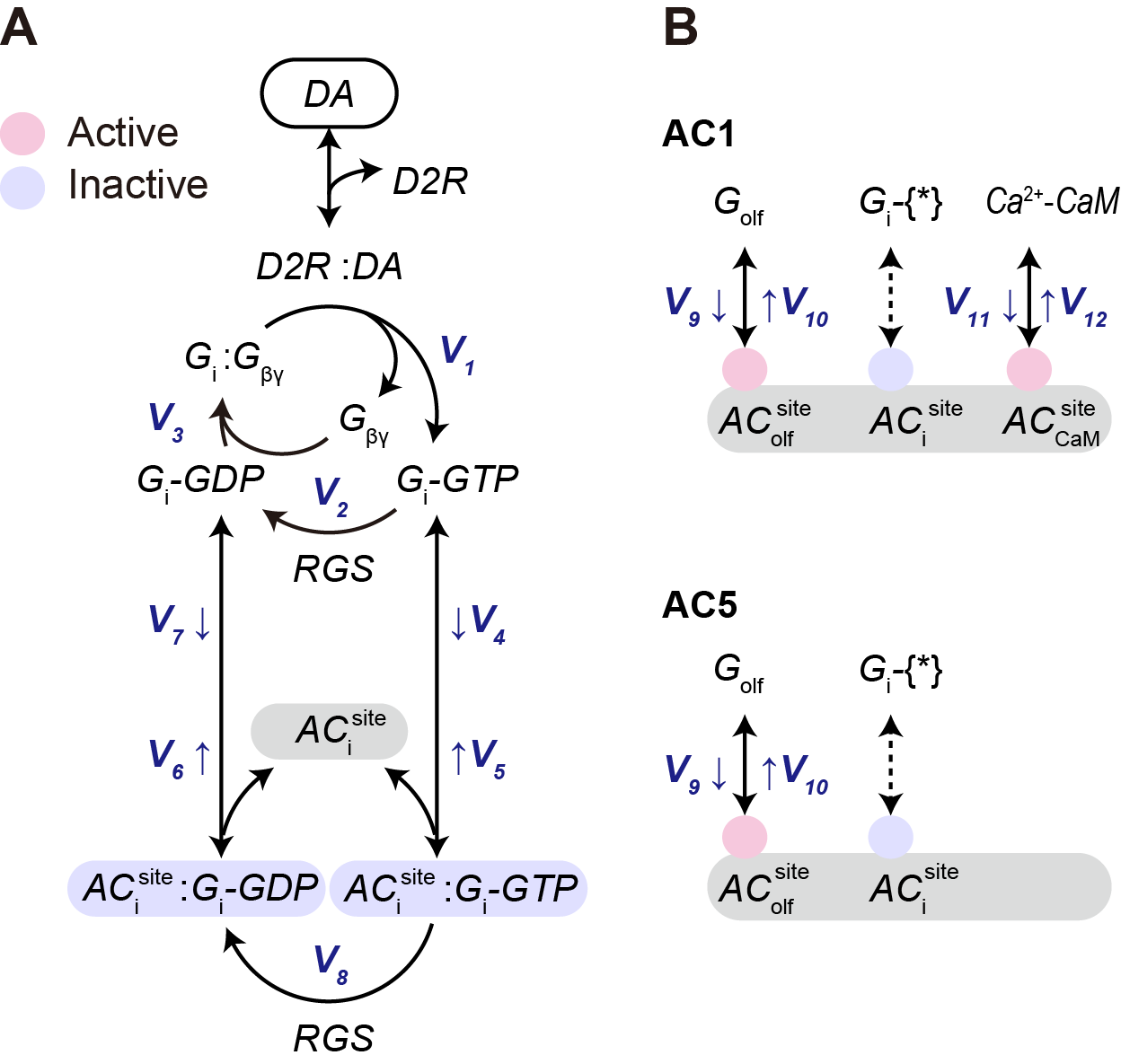

Supplement: S1 Fig — Arrows denote the first order reactions or enzymatic reactions, and their fluxes are denoted by V1,…, V12. (A) Reactions of the D2R–Gi–AC part. The input DA (circled) first regulates a Gi-protein cycle (top), then Gi-AC binding cycle (bottom). Gi-GTP/ Gi-GDP binds to a specific site of AC (ACisite). (B) Under the scheme of non-competitive binding, Golf, Gi, and Ca2+-CaM independently interact with their specific sites of AC (AColfsite, ACisite, and ACCaMsite, respectively). Simultaneous binding of Golf and Ca2+-CaM is required for the activity of AC1 (top), and the binding of Golf alone leads to the activity of AC5 (bottom). The binding of Gi inhibits the activities of both AC1 and AC5. Reactions with dashed arrows are the same as the reactions V4,…, V7 indicated in panel A. (TIF) [file pcbi.1009364.s001.tif]

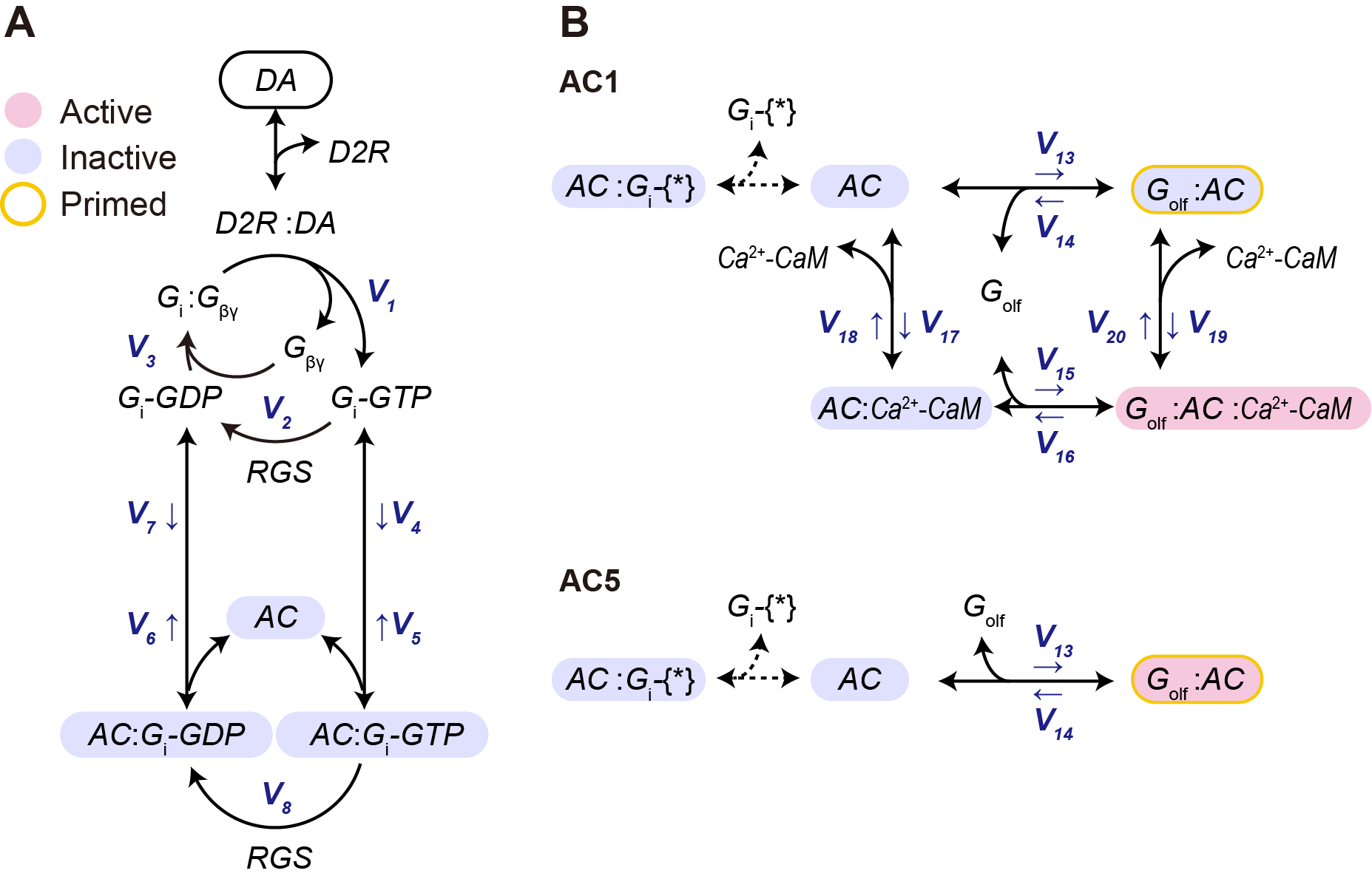

Supplement: S2 Fig — Arrows denote the first order reactions or enzymatic reactions, and their fluxes are denoted by V1,…, V20. (A) Reactions of the D2R–Gi–AC part. Same as S1A Fig, but the Gi binding to AC disables the binding of Golf and Ca2+-CaM. (B) If AC is free from Gi, the AC can interact with Golf and Ca2+-CaM. Simultaneous binding of Golf and Ca2+-CaM is required for the activity of AC1 (top), and the binding of Golf alone sufficiently activates AC5 (bottom). Under the competitive binding, ACprimed corresponds to the levels of yellow-bordered states. Reactions with dashed arrows are the same as the reactions V4,…, V7 indicated in panel A. (TIF) [file pcbi.1009364.s002.tif]

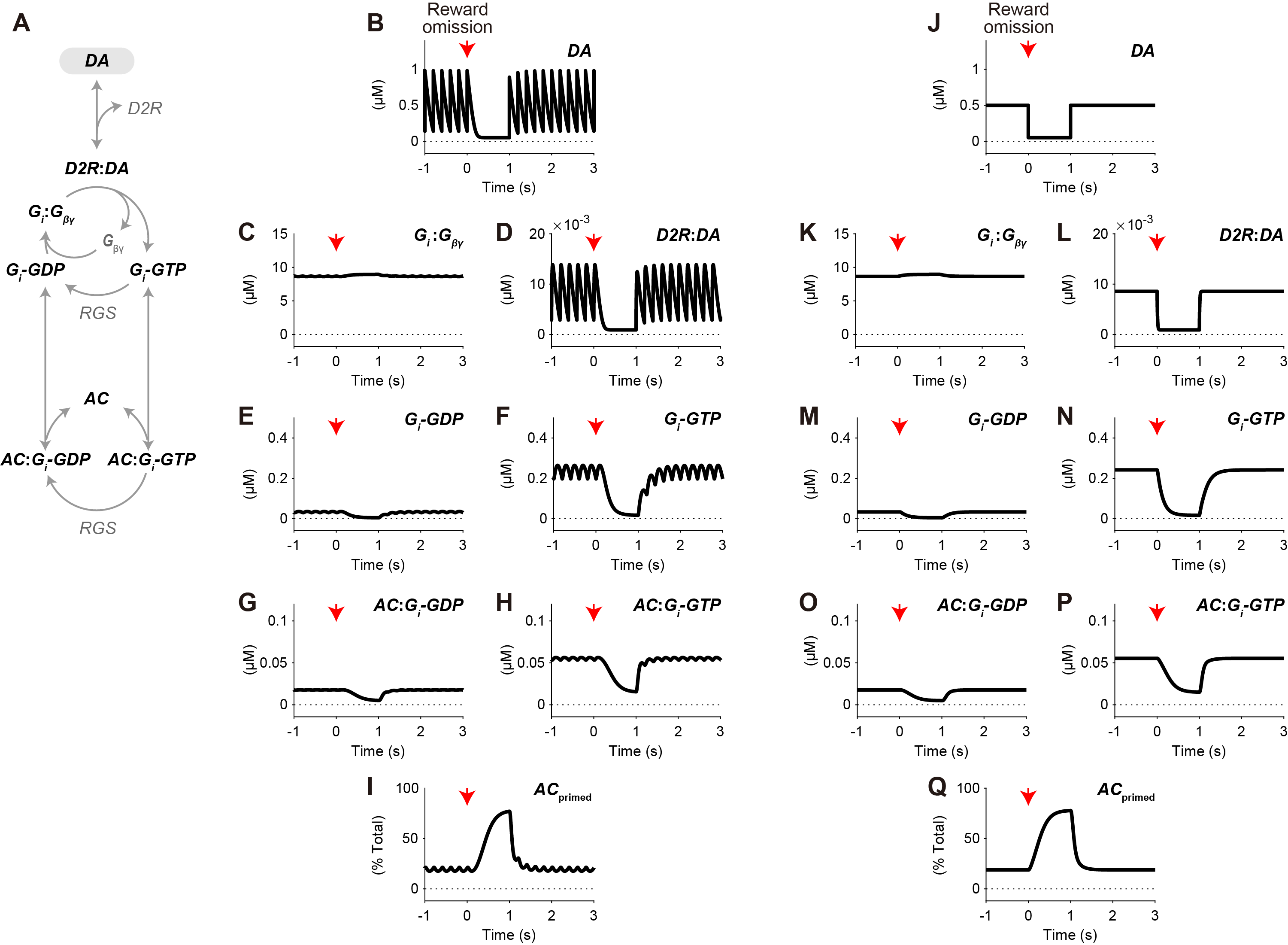

Supplement: S3 Fig — Red arrows denote the onset of a 1-s DA dip. (A) Observed molecules. (B) Optogenetically-evoked dynamics of DA. (C–I) Subsequent molecular activities. (J) Square-drop signal as a representative of DA dip. (K-Q) Subsequent molecular activities. (TIF) [file pcbi.1009364.s003.tif]

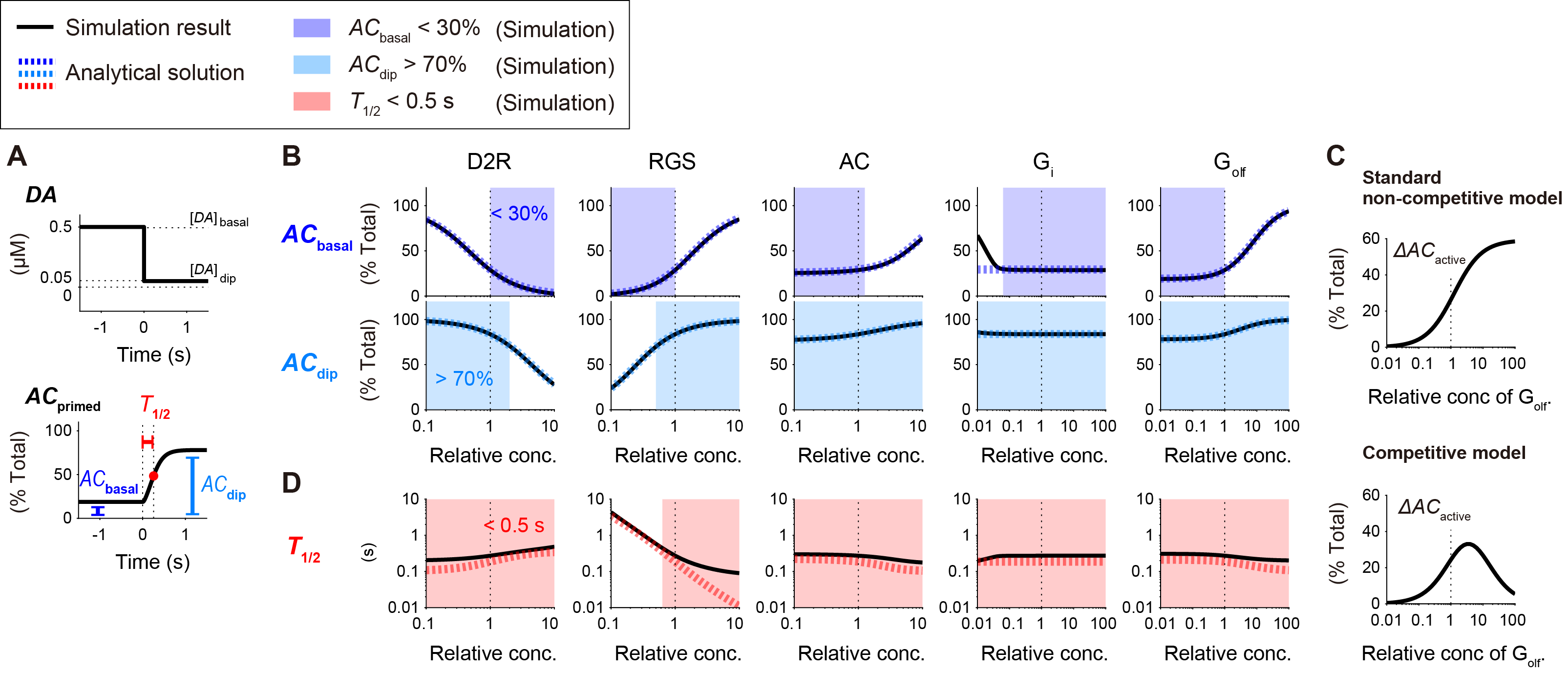

Supplement: S4 Fig — (A) ACprimed is observed under the stepwise decreasing signal of DA. (B) Concentration dependence of the steady state levels of ACprimed, ACbasal and ACdip in the competitive model. (C) Golf-dependence of ΔACactivity in the non-competitive and competitive models (see Eqs (7) and (10)). ΔACactive=ACactive|[DA]=[DA]dip−ACactive|[DA]=[DA]basal where ACactive|[DA]=[DA]basal and ACactive|[DA]=[DA]dip denote the activities of AC5 under [DA]basal and [DA]dip, respectively. The biphasic Golf concentration dependence appears only in the competitive model, as shown in Bruce et al. (2019) [20]. (D) Parameter dependence of T1/2 in the competitive model. (TIF) [file pcbi.1009364.s004.tif]

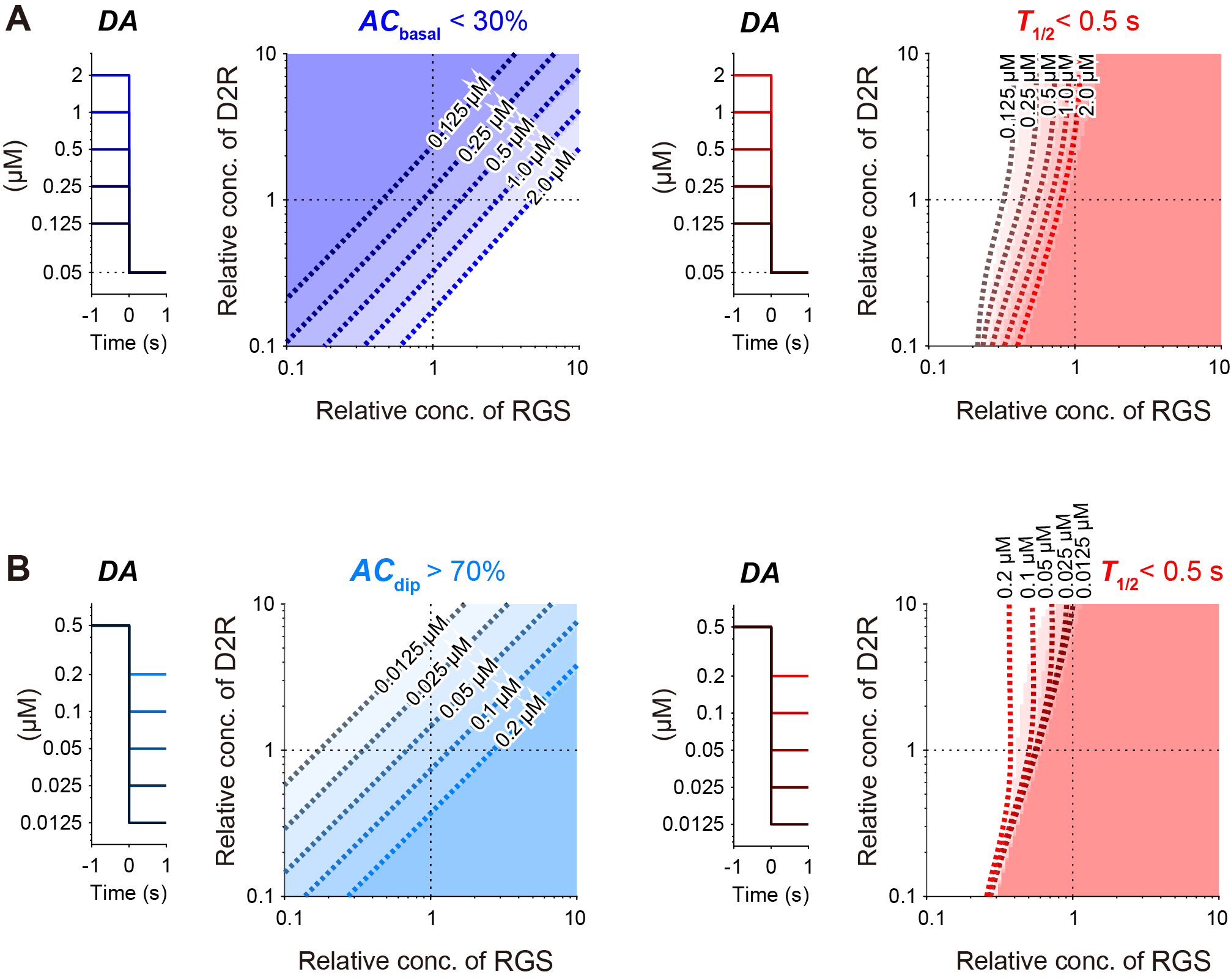

Supplement: S5 Fig — (A) [DA]basal is set to be 0.125, 0.25, …, 4 μM (left), and the areas of ACbasal < 30% (top) and T1/2 < 0.5 s (bottom) are plotted in the space of [RGS]tot and [DA]tot. Dotted lines denote the analytical solutions, and colored areas denote the simulation results. (B) [DA]dip is set to be 0.0125, 0.025, …, 0.4 μM (left), and the areas of ACdip > 70% (top) and T1/2 < 0.5 s (bottom) are plotted. (TIF) [file pcbi.1009364.s005.tif]

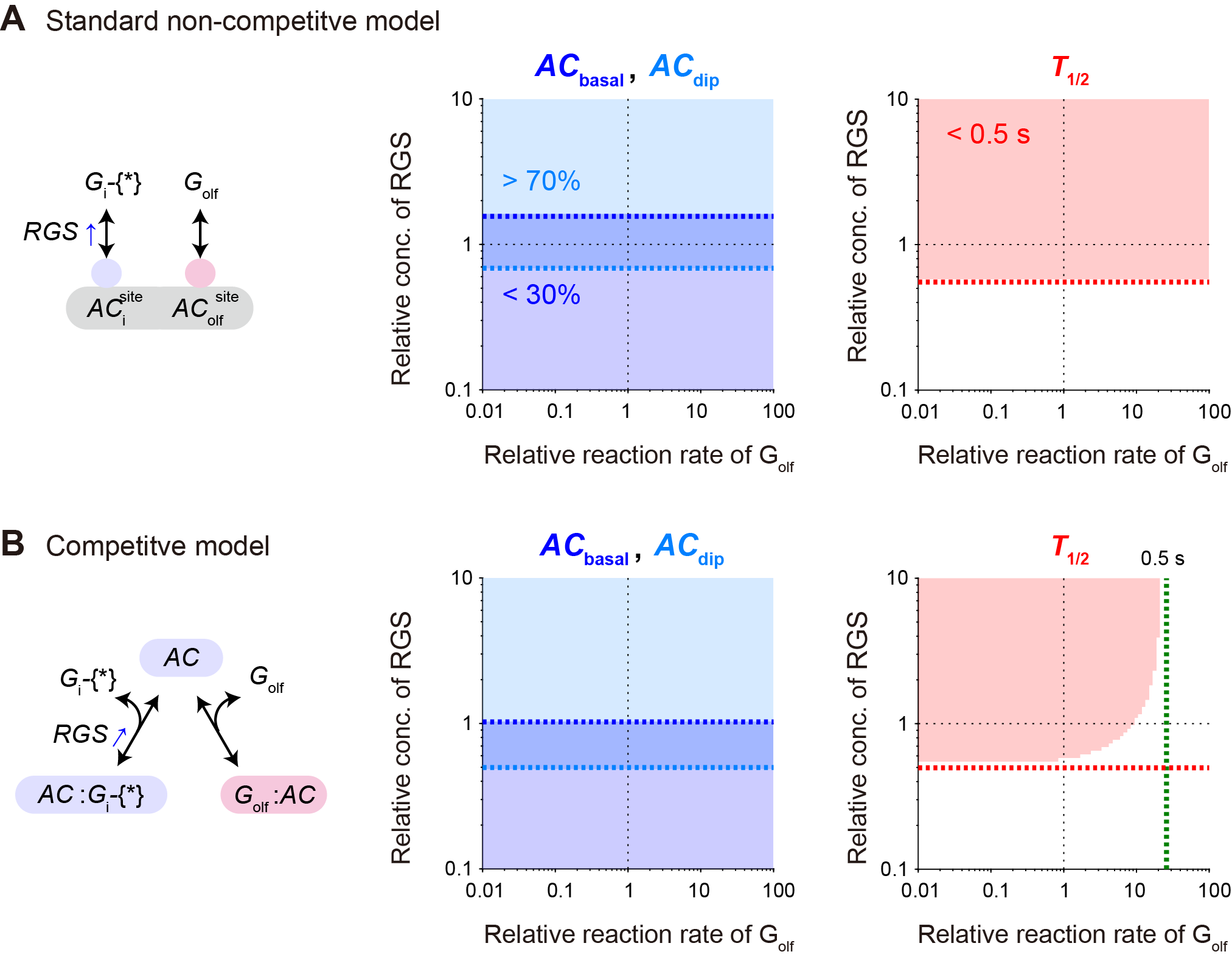

Supplement: S6 Fig — Here, the binding/unbinding reaction rate of Golf, τ = 1/(kon,Golf[Golf]buff+koff,Golf), is subjected to change, whereas Kd,Golf (= koff,Golf / kon,Golf) and [Golf]buff are kept constant. (A, B) ACbasal, ACdip, and T1/2 in the standard non-competitive model (A), and competitive model (B). Green dotted line in panel B denotes τ = 0.5/log 2 (T1/2 = 0.5 s). (TIF) [file pcbi.1009364.s006.tif]

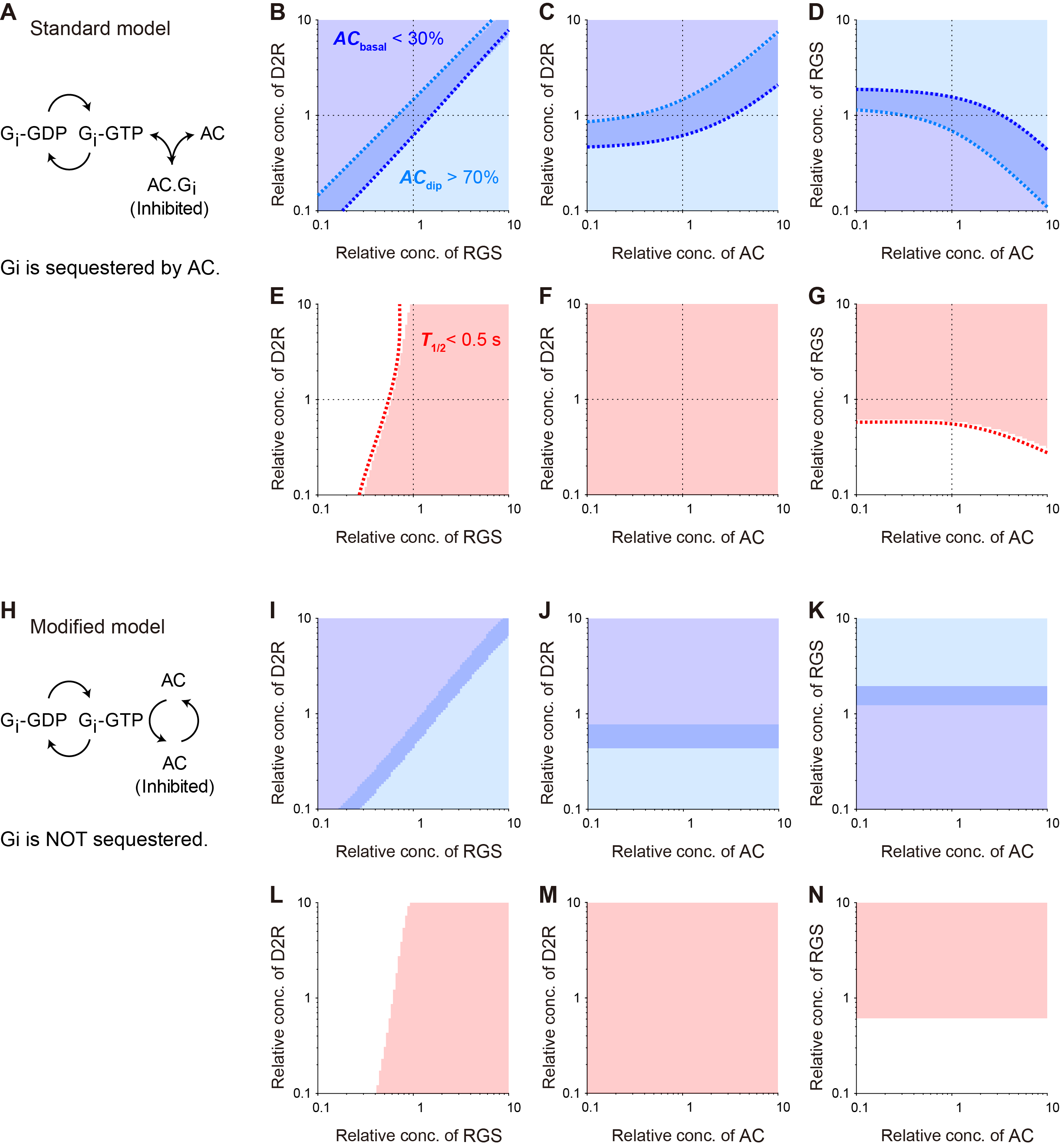

Supplement: S7 Fig — (A–G) Areas of ACbasal < 30% (blue), ACdip > 70% (light blue), and T1/2 < 0.5 s (pink) in the standard non-competitive model (A). These areas are plotted in the spaces of [D2R]tot versus [RGS]tot (B, E), [D2R]tot versus [AC]tot (C, F), and [RGS]tot versus [AC]tot (D, G). Dotted lines denote their analytical solutions. (H–N) Same as panels A–G, but the standard D2 model is modified so that AC sequesters Gi, i.e., V4, V5, V6, and V7 are removed only from Eq (S3) but not from Eq (S12). (TIF) [file pcbi.1009364.s007.tif]

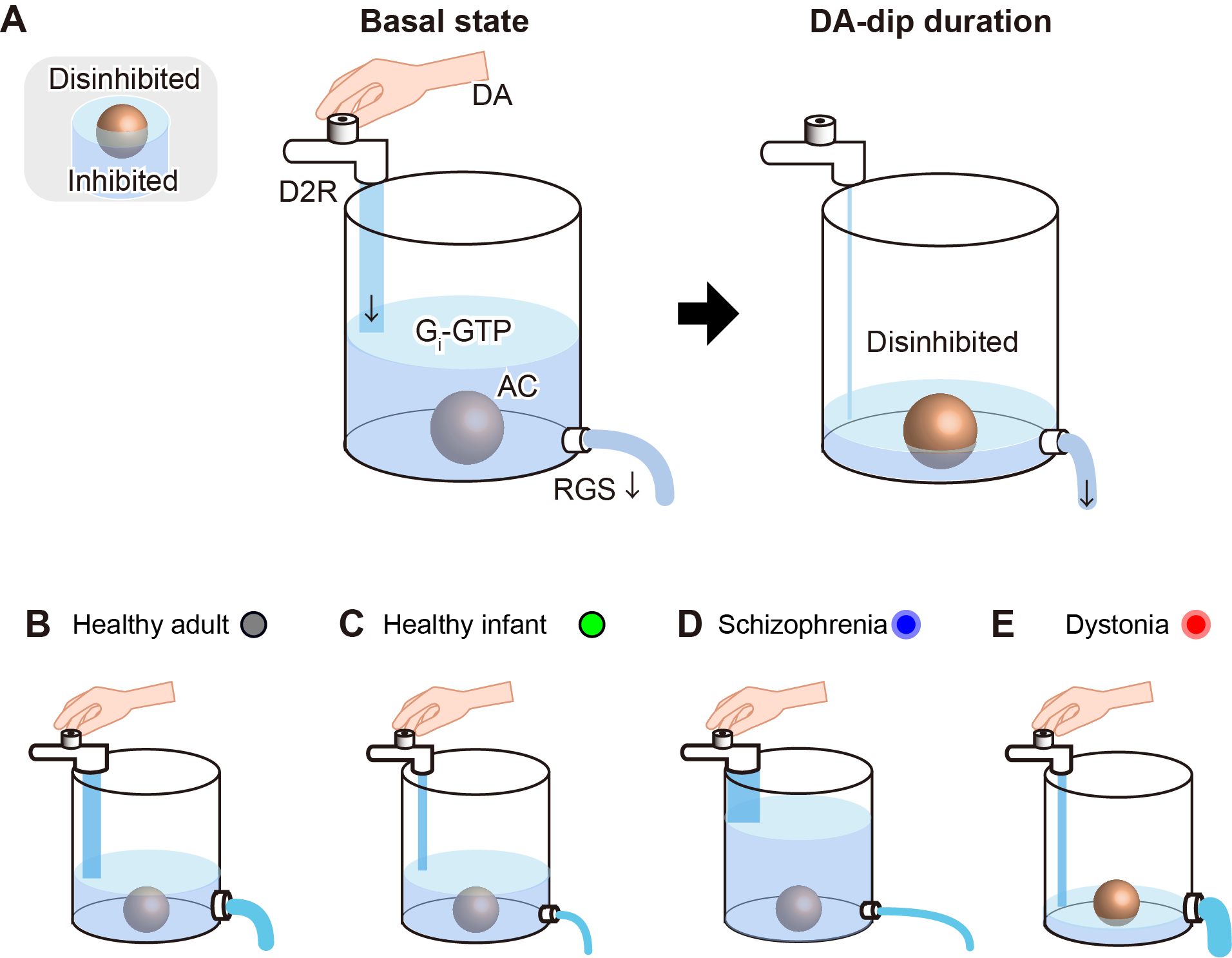

Supplement: S8 Fig — (A) At the steady state (left), DA-bound D2R provides a constant flux of Gi-GTP (V1; see section A in S1 Appendix), while the Gi-GTP is drained through RGS with a speed (V2 + V8) that is proportional to the amount of Gi-GTP. Difference in the fluxes generates a pool of Gi-GTP that inhibits AC, which is represented by a sunk ball (AC). During the period of a DA dip (right), the supply of Gi-GTP nearly completely stops, and the pooled Gi-GTP is drained rapidly. AC is then disinhibited to be activated by Golf and Ca2+-CaM. (B-E) The Gi inhibition of AC in the healthy adult, healthy infant, schizophrenia, and dystonia, all of which are described in Fig 6A−6E. (C) In the healthy-infant model, the influx and efflux of Gi-GTP are both small, and the smaller efflux causes delayed disinhibition of AC during a DA dip. (D) In the schizophrenia model, hyperactive D2R provides a larger amount of Gi-GTP, thus AC is fully inhibited and its disinhibition is delayed. (E) The larger efflux of Gi-GTP in the dystonia model results in the chronic activity of AC. (TIF) [file pcbi.1009364.s008.tif]

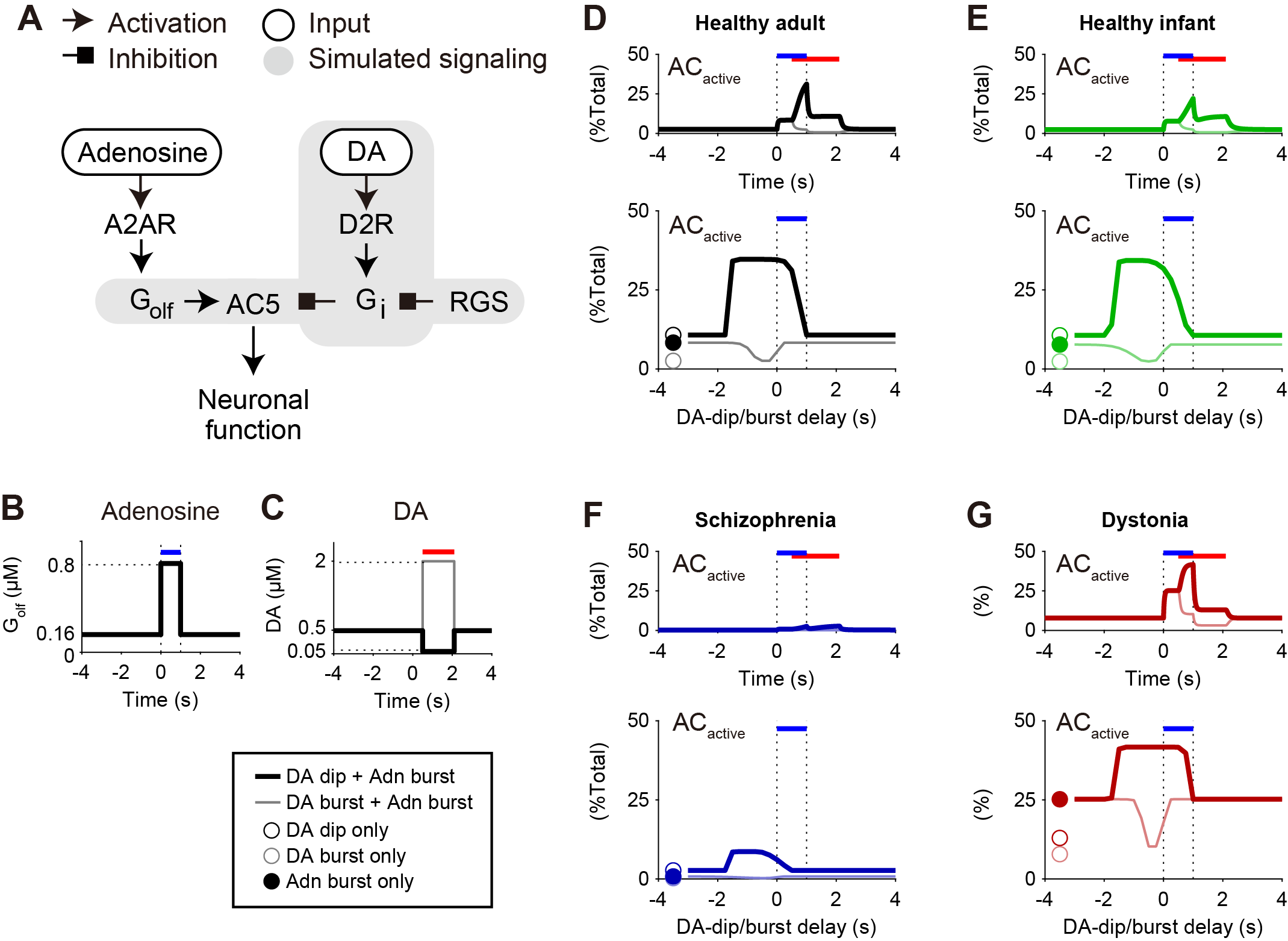

Supplement: S9 Fig — A transient burst in adenosine (thus Golf) coincides with a DA dip/burst with a variety of delay times. (A) Schematic of AC5 signaling. (B) Adenosine burst as a square wave of Golf (basal, 0.16 μM; burst, 0.8 μM; duration, 1 s). (C) Square-wave dip and burst of DA (basal, 0.5 μM; dip, 0.05 μM; burst, 2 μM; duration, 2 s). (D-G) Example traces of the AC5 activities (ACactive; top), and DA-dip/burst delay dependence of the maximal ACactive (bottom) in the healthy-adult, healthy-infant, schizophrenia, and dystonia models. In the example traces, adenosine and DA signals were given during the periods indicated by blue and red bars, respectively. Similar simulation (adenosine versus transient DA dips) has been conducted by Nair et al. (2015) [24]. (TIF) [file pcbi.1009364.s009.tif]
