# Supplementary material for: The critical balance between dopamine D2 receptor and RGS for the sensitive detection of a transient decay in dopamine signal
Source: PLoS Comput Biol. 2021 Sep 30;17(9):e1009364. doi: 10.1371/journal.pcbi.1009364 (PMC8483376; doi:10.1371/journal.pcbi.1009364)
Supplement: S1 Table — Densities of membrane molecules should have the unit of membrane area (/μm2), but not volume (μM). However, many of the referenced experiments/simulations have described them under homogenate conditions; therefore, we also adopted volume concentration for consistency. Note that Xarea /μm2 ~ Xvolume μM × 20, because a spherical spine with a radius rspine ~ 0.1 μm has the number of surface molecules Xarea × (4πrspine2), and the number of cytosolic molecules is Xvolume × 10−6NA × [4πrspine3/3 × 10−15] where NA = 6.02 ×1023 (Avogadro constant). (PDF) [file pcbi.1009364.s010.pdf]

S1 Table

Molecular name	Concentration	Notes and references
DA (Buffered)	$[DA]_{basal}$ = 0.5 μM, $[DA]_{dip}$ = 0.05 μM, $[DA]_{opto}$ = 0.841 μM	$[DA]_{basal}$ and $[DA]_{dip}$ were determined based on Iino et al. [1]. $[DA]_{opto}$ was set to give an average concentration of 0.5 μM under 5-Hz stimulation. The DA level was not affected by the binding to D2R (buffered).
D2R	0.18 μM	In many types of cells, the molar ratio of G-protein coupled receptor, G protein, and AC is ~1: 100: 3 [2]. Also, the concentration of D2R in the NAc (0.6~0.8 pmol/mg) is by two orders smaller than that of G_{olf}:$G_{\beta\gamma}$ [3,4], and the amount of G_{olf} is similar to that of G_i [5]. We here set D2R: G_i = 1: 50 because D2R is expressed in D2 SPNs, but not in D1 SPNs. The total amount of G_{olf} and G_i seems to be smaller than that of $G_{\beta\gamma}$ [6], and the molar ratio of G_{olf} and AC is ~ 30: 1 [7]. We set G_i: AC = 100: 1 because G_{olf}: G_i is ~ 1: 1, and the punctate localization of AC1 was excluded [8].
G_i	9 μM	
$G_{\beta\gamma}$ (Buffered)	6 μM	
AC	0.09 μM	
RGS	0.9 μM	RGS9–2 is highly expressed in the striatum [9]. The molar ratio of RGS9–2 and $G_{\beta5}$ in the striatum is 4: 8 [10]. $G_{\beta5}$ was assumed to share 20% of total G_β [11].
G_{olf} (Buffered)	$[G_{olf}]_{buff}$ = 0.8 μM	$[G_{olf}]_{buff}$ denotes a fixed and buffered concentration of G_{olf}-GTP, which is continuously provided by active A2AR. The upper-bound concentration of G_{olf}-GTP, $[G_{olf}]_{tot}$, is ~ $[G_i]_{tot}$ [5].

References

1. Iino Y, Sawada T, Yamaguchi K, Tajiri M, Ishii S, Kasai H, et al. Dopamine D2 receptors in discrimination learning and spine enlargement. Nature. 2020; 579(7800): 555-560. Epub 2020/03/28. pmid: 32214250.
2. Ostrom RS, Post SR, Insel PA. Stoichiometry and compartmentation in G protein-coupled receptor signaling: implications for therapeutic interventions involving G(s). J Pharmacol Exp Ther. 2000; 294(2): 407-412. pmid: 10900212.
3. Boyson SJ, McGonigle P, Molinoff PB. Quantitative autoradiographic localization of the D1 and D2 subtypes of dopamine receptors in rat brain. J Neurosci. 1986; 6(11): 3177-3188. Epub 1986/11/01. pmid: 3534157.
4. Herve D. Identification of a specific assembly of the g protein Golf as a critical and regulated module of dopamine and adenosine-activated cAMP pathways in the striatum. Front Neuroanat. 2011; 5: 48. Epub 2011/09/03. pmid: 21886607.
5. Cai G, Wang HY, Friedman E. Increased dopamine receptor signaling and dopamine receptor-G protein coupling in denervated striatum. J Pharmacol Exp Ther. 2002; 302(3): 1105-1112. pmid: 12183669.
6. Schwindinger WF, Mihalcik LJ, Giger KE, Betz KS, Stauffer AM, Linden J, et al. Adenosine A_{2A} receptor signaling and G_{olf} assembly show a specific requirement for the γ7 subtype in the striatum. J Biol Chem. 2010; 285(39): 29787-29796. Epub 2010/07/20. pmid: 20639202.
7. Zalduegui A, Lopez de Jesus M, Callado LF, Meana JJ, Salles J. Levels of $Gs\alpha_{(short\ and\ long)}$, $G\alpha_{(olf)}$ and $G\beta_{(common)}$ subunits, and calcium-sensitive adenylyl cyclase isoforms (1, 5/6, 8) in post-mortem human brain caudate and cortical membranes: comparison with rat brain membranes and potential stoichiometric relationships. Neurochem Int. 2011; 58(2): 180-189. Epub 2010/12/01. pmid: 21115086.
8. Visel A, Alvarez-Bolado G, Thaller C, Eichele G. Comprehensive analysis of the expression patterns of the adenylate cyclase gene family in the developing and adult mouse brain. J Comp Neurol. 2006; 496(5): 684-697. pmid: 16615126.
9. Gold SJ, Ni YG, Dohlman HG, Nestler EJ. Regulators of G-protein signaling (RGS) proteins: Region-specific expression of nine subtypes in rat brain. Journal of Neuroscience. 1997; 17(20): 8024-8037. pmid: ISI:A1997XZ44900042.
10. Anderson GR, Lujan R, Martemyanov KA. Changes in Striatal Signaling Induce Remodeling of RGS Complexes Containing Gβ5 and R7BP Subunits. Molecular and Cellular Biology. 2009; 29(11): 3033-3044. pmid: ISI:000266006500010.
11. Betty M, Harnish SW, Rhodes KJ, Cockett MI. Distribution of heterotrimeric G-protein beta and gamma subunits in the rat brain. Neuroscience. 1998; 85(2): 475-486. Epub 1998/06/11. pmid: 9622245.
