# Supplementary material for: The critical balance between dopamine D2 receptor and RGS for the sensitive detection of a transient decay in dopamine signal
Source: PLoS Comput Biol. 2021 Sep 30;17(9):e1009364. doi: 10.1371/journal.pcbi.1009364 (PMC8483376; doi:10.1371/journal.pcbi.1009364)
Supplement: S2 Table — (PDF) [file pcbi.1009364.s011.pdf]

S2 Table

Reaction name	Rate constant	Notes and references
$K_{m,DAT}$	0.2 μM	$K_{m,DAT}$ is ~0.2 μM [1], and $k_{cat,DAT}[DAT]$ was set to give a half-valued period ($t_{1/2,DA}$) of ~ 0.1 s [2,3].
$k_{cat,DAT}[DAT]$	8.9 $\mu M^{-1}s^{-1}$	
$k_{on,DA}$	10 $\mu M^{-1}s^{-1}$	DA binds to D2R with a time constant of $t_{1/2}$ ~ 30 ms [2,4]. $K_{d,DA}$ = 10 μM ($k_{off,DA}/k_{on,DA}$) was taken from the previous study [5,6], which corresponds to that at the low affinity sites of D2R (2.5 μM) [7]. In Yapo et al. (2017), $k_{off,DA}$ is also set to be 100 s^{-1} [5].
$k_{off,DA}$	100 s^{-1}	
$k_{on,G\beta\gamma}$	10 $\mu M^{-1}s^{-1}$	O'Neill et al. [8].
$k_{cat,exch,Gi}$	230 s^{-1}	Biochemical experiments show that $K_{m,exch,Gi}$ of D2R for G_i is ~1 nM (Fig 3 in Senogles et al.) [9], and $K_{m,exch,Gi}$ of rhodopsin for G_t , which is similar to D2R for G_i, is much smaller than 0.2 μM [10]. The catalytic rate constant, $k_{cat,exch,Gi}$, was determined to be consistent with the rapid response of G_i-dependent GIRK current in a physiological experiment [11]. In general, k_{cat} is very sensitive to the experimental condition [12]. Thus, $k_{cat,exch,Gi}$ was determined based on the more reliable physiological experiment.
$K_{m,exch,Gi}$	0.01 μM	
$k_{on,GiGTP}$	200 $\mu M^{-1}s^{-1}$	IC_{50}s of G_i-GTP for AC1 and AC5 are 0.04~0.18 μM [13]. We assume that IC_{50} ~ $K_{d,,}$ which is valid under non-competitive inhibition and the G_i-buffering condition. $K_{d,GiGTP}$ was thus set to be 0.04 μM. Also, IC_{50} of G_i-GTP for an AC is ~0.037 μM, and IC_{50} of G_i-GDP for the AC is ~1 μM. $K_{d,GiGTP}$ was thus assumed to be 27-fold lower than $K_{d,GiGDP}$. The rate constants were assumed because k_{cat} depends on the experimental condition [12].
$k_{off,GiGTP}$	8 s^{-1}	
$k_{on,GiGDP}$	20 $\mu M^{-1}s^{-1}$	
$k_{off,GiGDP}$	21.6 s^{-1}	
$k_{cat,hyd,Gi}$	90 s^{-1}	$K_{m,hyd,Gi}$ of RGS4, 7, and 8 are ≫ 3 μM [14], and $K_{m,hyd}$ of RGS9 for G_t is ~12 μM [15]. The product turnover rate, $k_{cat,hyd,Gi}$, was assumed because k_{cat} depends on the experimental condition [12].
$K_{m,hyd,Gi}$	12 μM	
$k_{on,Golf}$	20 $\mu M^{-1}s^{-1}$	Bruce et al. [16]. We assumed that G_{olf}-GTP rapidly binds to AC, then autocatalytically hydrolyzed G_{olf}-GDP is rapidly detached from AC.
$k_{off,Golf}$	20 s^{-1}	

References

1. Dreyer JK, Herrik KF, Berg RW, Hounsgaard JD. Influence of phasic and tonic dopamine release on receptor activation. J Neurosci. 2010; 30(42): 14273-14283. pmid: 20962248.
2. Patriarchi T, Cho JR, Merten K, Howe MW, Marley A, Xiong WH, et al. Ultrafast neuronal imaging of dopamine dynamics with designed genetically encoded sensors. Science. 2018; 360(6396). pmid: 29853555.
3. Labouesse MA, Cola RB, Patriarchi T. GPCR-Based Dopamine Sensors-A Detailed Guide to Inform Sensor Choice for In vivo Imaging. Int J Mol Sci. 2020; 21(21). Epub 2020/11/01. pmid: 33126757.
4. Lohse MJ, Nikolaev VO, Hein P, Hoffmann C, Vilardaga JP, Bunemann M. Optical techniques to analyze real-time activation and signaling of G-protein-coupled receptors. Trends Pharmacol Sci. 2008; 29(3): 159-165. Epub 2008/02/12. pmid: 18262662.
5. Yapo C, Nair AG, Clement L, Castro LR, Hellgren Kotaleski J, Vincent P. Detection of phasic dopamine by D1 and D2 striatal medium spiny neurons. J Physiol. 2017; 595(24): 7451-7475. pmid: 28782235.
6. Urakubo H, Yagishita S, Kasai H, Ishii S. Signaling models for dopamine-dependent temporal contiguity in striatal synaptic plasticity. PLoS Comput Biol. 2020; 16(7): e1008078. Epub 2020/07/24. pmid: 32701987.
7. So CH, Varghese G, Curley KJ, Kong MM, Alijaniaram M, Ji X, et al. D1 and D2 dopamine receptors form heterooligomers and cointernalize after selective activation of either receptor. Mol Pharmacol. 2005; 68(3): 568-578. pmid: 15923381.
8. O'Neill PR, Karunarathne WK, Kalyanaraman V, Silvius JR, Gautam N. G-protein signaling leverages subunit-dependent membrane affinity to differentially control betagamma translocation to intracellular membranes. Proc Natl Acad Sci U S A. 2012; 109(51): E3568-3577. Epub 2012/12/06. pmid: 23213235.
9. Senogles SE, Spiegel AM, Padrell E, Iyengar R, Caron MG. Specificity of receptor-G protein interactions. Discrimination of Gi subtypes by the D2 dopamine receptor in a reconstituted system. J Biol Chem. 1990; 265(8): 4507-4514. Epub 1990/03/15. pmid: 2137824.
10. Randazzo PA, Jian X, Chen PW, Zhai P, Soubias O, Northup JK. Quantitative Analysis of Guanine Nucleotide Exchange Factors (GEFs) as Enzymes. Cell Logist. 2013; 3: e27609. Epub 2013/01/01. pmid: 25332840.
11. Marcott PF, Mamaligas AA, Ford CP. Phasic dopamine release drives rapid activation of striatal D2-receptors. Neuron. 2014; 84(1): 164-176. pmid: 25242218.
12. Daniel RM, Danson MJ. Temperature and the catalytic activity of enzymes: a fresh understanding. FEBS Lett. 2013; 587(17): 2738-2743. Epub 2013/07/03. pmid: 23810865.
13. Taussig R, Tang WJ, Hepler JR, Gilman AG. Distinct patterns of bidirectional regulation of mammalian adenylyl cyclases. J Biol Chem. 1994; 269(8): 6093-6100. pmid: 8119955.
14. Lan KL, Zhong HL, Nanamori M, Neubig RR. Rapid kinetics of regulator of G-protein signaling (RGS)-mediated $G\alpha_i$ and $G\alpha_o$ deactivation - $G\alpha$ specificity of RGS4 and RGS7. Journal of Biological Chemistry. 2000; 275(43): 33497-33503. pmid: ISI:000090104600045.
15. Martemyanov KA, Arshavsky VY. Kinetic approaches to study the function of RGS9 isoforms. Methods Enzymol. 2004; 390: 196-209. pmid: 15488179.
16. Bruce NJ, Narzi D, Trpevski D, van Keulen SC, Nair AG, Rothlisberger U, et al. Regulation of adenylyl cyclase 5 in striatal neurons confers the ability to detect coincident neuromodulatory signals. PLoS Comput Biol. 2019; 15(10): e1007382. Epub 2019/10/31. pmid: 31665146.
