# Supplementary material for: The critical balance between dopamine D2 receptor and RGS for the sensitive detection of a transient decay in dopamine signal
Source: PLoS Comput Biol. 2021 Sep 30;17(9):e1009364. doi: 10.1371/journal.pcbi.1009364 (PMC8483376; doi:10.1371/journal.pcbi.1009364)
Supplement: S1 Appendix — (A) Definition of the D2 model. (B) Derivation of analytical ACbasal and ACdip. (C) Derivation of analytical T1/2. (PDF) [file pcbi.1009364.s012.pdf]

S1 Appendix:

“The critical balance between dopamine D2 receptor and RGS for the sensitive detection of a transient decay in dopamine signal”

Hidetoshi Urakubo*, Sho Yagishita, Haruo Kasai, Yoshiyuki Kubota, and Shin Ishii

* hurakubo@gmail.com (HU)

A. Definition of the D2 model

Case 1: Standard non-competitive model

We first built the D2 model under the non-competitive binding between G_{olf} and "G_i, and Ca^{2+}-CaM," which is called the standard non-competitive model (**S1 Fig**). In the standard model, the concentration of DA-bound D2R, [*D2R*:*DA*], was given by (**S1A Fig**):

$$\frac{d}{dt}[D2R{:}DA] = k_{\mathrm{on,DA}}[D2R][DA] - k_{\mathrm{off,DA}}[D2R]\,, \tag{S1}$$

$$[D2R]_{tot} = [D2R] + [D2R{:}DA]\,, \tag{S2}$$

where the reaction rate constants, k_x, are summarized in **S2 Table**. The G_i cycle was formulated as:

$$\frac{d}{dt}\begin{pmatrix}[G_\mathrm{i}\text{-}GTP]\\ [G_\mathrm{i}\text{-}GDP]\\ [G_\mathrm{i}{:}G_{\beta\gamma}]\\ [G_{\beta\gamma}]\end{pmatrix} = \begin{pmatrix}V_1 - V_2 - V_4 + V_5\\ V_2 - V_3 + V_7 - V_6\\ -V_1 + V_3\\ V_1 - V_3\end{pmatrix}, \tag{S3}$$

$$[G_\mathrm{i}]_{tot} = [G_\mathrm{i}\text{-}GTP] + [G_\mathrm{i}\text{-}GDP] + [G_\mathrm{i}{:}G_{\beta\gamma}] + [AC_\mathrm{i}^\mathrm{site}{:}G_\mathrm{i}\text{-}GTP] + [AC_\mathrm{i}^\mathrm{site}{:}G_\mathrm{i}\text{-}GDP]\,, \tag{S4}$$

where $AC_\mathrm{i}^\mathrm{site}$ is the G_i binding site of AC. The fluxes, V_1, …,V_7, were formulated as:

$$V_1 = \frac{k_\mathrm{cat,exch,Gi}[D2R{:}DA][G_\mathrm{i}{:}G_{\beta\gamma}]}{(K_\mathrm{m,exch} + [G_i{\cdot}G_{\beta\gamma}])}\,, \tag{S5}$$

$$V_2 = \frac{k_\mathrm{cat,hyd,Gi}[RGS]_\mathrm{tot}[G_\mathrm{i}\text{-}GTP]}{(K_\mathrm{m,hyd,Gi} + [G_\mathrm{i}\text{-}GTP])}\,, \tag{S6}$$

$$V_3 = k_{\beta\gamma}[G_{\beta\gamma}]_\mathrm{buff}[G_\mathrm{i}\text{-}GDP]\,, \tag{S7}$$

$$V_4 = k_\mathrm{on,GiGTP}[AC_\mathrm{i}^\mathrm{site}][G_\mathrm{i}\text{-}GTP]\,, \tag{S8}$$

$$V_5 = k_\mathrm{off,GiGTP}[AC_\mathrm{i}^\mathrm{site}{:}G_\mathrm{i}\text{-}GTP]\,, \tag{S9}$$

$$V_6 = k_\mathrm{off,GiGDP}[AC_\mathrm{i}^\mathrm{site}{:}G_\mathrm{i}\text{-}GDP]\,, \tag{S10}$$

$$V_7 = k_\mathrm{on,GiGDP}[AC_\mathrm{i}^\mathrm{site}][G_\mathrm{i}\text{-}GDP]\,. \tag{S11}$$

Similarly, the binding reactions of G_i to AC were formulated as:

$$\frac{d}{dt}\begin{pmatrix}[AC_\mathrm{i}^\mathrm{site}{:}G_\mathrm{i}\text{-}GTP]\\ [AC_\mathrm{i}^\mathrm{site}{:}G_\mathrm{i}\text{-}GDP]\end{pmatrix} = \begin{pmatrix}V_4 - V_5 - V_8\\ -V_6 + V_7 + V_8\end{pmatrix}, \tag{S12}$$

$$[AC]_{\text{tot}} = \left[AC_{\text{i}}^{\text{site}}\right] + \left[AC_{\text{i}}^{\text{site}}\text{: } G_i\text{-}GTP\right] + \left[AC_{\text{i}}^{\text{site}}\text{: } G_i\text{-}GDP\right]. \qquad (S13)$$

The fluxes, V_8, was formulated as:

$$V_8 = \frac{k_{\text{cat,hyd,Gi}}[RGS]_{\text{tot}}[AC_{\text{i}}^{\text{site}}\text{: } G_i\text{-}GTP]}{\left(K_{\text{m,hyd,Gi}} + [AC_{\text{i}}^{\text{site}}\text{: } G_i\text{-}GTP]\right)}. \qquad (S14)$$

We then modeled G_{olf} signaling. In our primary target, a continuous A2AR activity produced a constant and fixed concentration of G_{olf}-GTP, $[G_{\text{olf}}]_{\text{buff}}$ [1]. The G_{olf}-GTP bound to a specific site of AC, $AC_{\text{olf}}^{\text{site}}$, and was rapidly detached from AC due to its autonomous hydrolysis [2], as follows (**S1B Fig**):

$$\frac{d}{dt}\left[AC_{\text{olf}}^{\text{site}}\text{: } G_{\text{olf}}\right] = V_9 - V_{10}, \qquad (S15)$$

$$V_9 = k_{\text{on,Golf}}\left[AC_{\text{olf}}^{\text{site}}\right][G_{\text{olf}}]_{\text{buff}}, \qquad (S16)$$

$$V_{10} = k_{\text{off,Golf}}\left[AC_{\text{olf}}^{\text{site}}\text{: } G_{\text{olf}}\right], \qquad (S17)$$

$$[AC]_{\text{tot}} = \left[AC_{\text{olf}}^{\text{site}}\right] + \left[AC_{\text{olf}}^{\text{site}}\text{: } G_{\text{olf}}\right]. \qquad (S18)$$

We also simulated the increase in Ca^{2+}-CaM due to pre–post pairing. The pre–post pairing was constituted of 10-consective elemental pairs at 10 Hz during 0~1 s (**Fig 1D**). These pairs led to Ca^{2+} influx via VGCC and/or NMDAR. The Ca^{2+} bound to CaM, and was uptaken via a Ca^{2+} pump. The Ca^{2+}-CaM bound to a specific site of AC, $AC_{\text{CaM}}^{\text{site}}$, as follows (**S1B Fig**):

$$\frac{d}{dt}\left[AC_{\text{CaM}}^{\text{site}}\text{: } Ca^{2+}\text{-}CaM_i\right] = V_{11} - V_{12}, \qquad (S19)$$

$$V_{11} = k_{\text{on,CaM,i}}\left[AC_{\text{CaM}}^{\text{site}}\right][Ca^{2+}\text{-}CaM_i], \qquad (S20)$$

$$V_{12} = k_{\text{off,CaM,i}}\left[AC_{\text{CaM}}^{\text{site}}\text{: } Ca^{2+}\text{-}CaM_i\right], \qquad (S21)$$

$$[AC]_{\text{tot}} = \left[AC_{\text{CaM}}^{\text{site}}\right] + \sum_{i=1}^{9}\left[AC_{\text{CaM}}^{\text{site}}\text{: } Ca^{2+}\text{-}CaM_i\right]. \qquad (S22)$$

where $i = 1,\ldots,9$ represent the states of Ca^{2+}-CaM that come from the number of Ca^{2+} ions binding to the C-terminal lobe (0, 1, 2; three states) and N-terminal lobe (0, 1, 2; three states) of a CaM (3 × 3 states). Detailed scheme and parameters of the Ca^{2+} signaling are described in Urakubo et al. (2020) [3]. The MATLAB code of the full model is available at the public repository GitHub

(https://github.com/urakubo/ModelRP2.git).

We finally modeled the concentration dynamics of active-state AC, $[AC_{\text{active}}]$ (**S1B Fig**):

$$\frac{[AC_{\text{active}}]}{[AC]_{\text{tot}}} = \begin{cases} \frac{[AC_{\text{i}}^{\text{site}}]}{[AC]_{\text{tot}}} \cdot \frac{[AC_{\text{olf}}^{\text{site}}: G_{\text{olf}}]}{[AC]_{\text{tot}}} \cdot \sum_{i=1}^{9} \frac{[AC_{\text{CaM}}^{\text{site}}: Ca^{2+}\text{-}CaM_i]}{[AC]_{\text{tot}}} & \text{(AC1)} \\ \frac{[AC_{\text{i}}^{\text{site}}]}{[AC]_{\text{tot}}} \cdot \frac{[AC_{\text{olf}}^{\text{site}}: G_{\text{olf}}]}{[AC]_{\text{tot}}} & \text{(AC5)} \end{cases}, \tag{S23}$$

We picked up the shared part of AC1 and AC5, as a primed state of AC for the activity:

$$AC_{\text{primed}} = \frac{[AC_{\text{i}}^{\text{site}}]}{[AC]_{\text{tot}}} \cdot \frac{[AC_{\text{olf}}^{\text{site}}: G_{\text{olf}}]}{[AC]_{\text{tot}}} \cdot \frac{K_{\text{d,Golf}} + [G_{\text{olf}}]_{\text{buff}}}{[G_{\text{olf}}]_{\text{buff}}}. \tag{S24}$$

where $[G_{\text{olf}}]_{\text{buff}}/\{K_{\text{d,Golf}} + [G_{\text{olf}}]_{\text{buff}}\}$ denotes a normalization factor, the maximal ratio of AC that binds to G_{olf}.

Case 2: Competitive model

We next modeled the D2 model under the competitive binding between G_{i} and "G_{olf} and Ca^{2+}-CaM" (competitive model, **S2 Fig**). The formulas of the G_{i} cycle were identical to Eqs (S1), …, (S7) (**S2A Fig**), and the G_{i}–AC binding reactions were also identical to Eqs (S8), …, (S12), and (S14), except that $AC_{\text{i}}^{\text{site}}$ was replaced with *AC*. The binding reactions of AC1 were formalized as (**S2B Fig**, top):

$$\frac{d}{dt}\begin{pmatrix} [AC] \\ [G_{\text{olf}}: AC] \\ [AC: Ca^{2+}\text{-}CaM_i] \\ [G_{\text{olf}}: AC: Ca^{2+}\text{-}CaM_i] \end{pmatrix} = \begin{pmatrix} V_5 + V_6 - V_4 - V_7 - V_{13} + V_{14} - V_{17} + V_{18} \\ V_{13} - V_{14} - V_{19} + V_{20} \\ V_{17} - V_{18} - V_{15} + V_{16} \\ V_{15} - V_{16} + V_{19} - V_{20} \end{pmatrix}, \tag{S25}$$

$$\begin{aligned} [AC]_{\text{tot}} &= [AC] + [AC: G_{\text{i}}\text{-}GTP] + [AC: G_{\text{i}}\text{-}GDP] + [G_{\text{olf}}: AC] \\ &+ \sum_{i=1}^{9} \{[AC: Ca^{2+}\text{-}CaM_i] + [G_{\text{olf}}: AC: Ca^{2+}\text{-}CaM_i]\}. \end{aligned} \tag{S26}$$

The binding reactions of AC5 were formalized as (**S2B Fig**, bottom):

$$\frac{d}{dt}\begin{pmatrix} [AC] \\ [G_{\text{olf}}: AC] \end{pmatrix} = \begin{pmatrix} V_5 + V_6 - V_4 - V_7 - V_{13} + V_{14} \\ V_{13} - V_{14} \end{pmatrix}, \tag{S27}$$

$$[AC]_{\text{tot}} = [AC] + [AC: G_{\text{i}}\text{-}GTP] + [AC: G_{\text{i}}\text{-}GDP] + [G_{\text{olf}}: AC] \tag{S28}$$

The fluxes, V_{13}, …,V_{20}, were formulated as:

$$V_{13} = k_{\text{on,Golf}}[G_{\text{olf}}][AC], \tag{S29}$$

$$V_{14} = k_{\text{off,Golf}}[G_{\text{olf}}{:}\,AC], \tag{S30}$$

$$V_{15} = k_{\text{on,Golf}}[G_{\text{olf}}][AC{:}\,Ca^{2+}\text{-}CaM_i], \tag{S31}$$

$$V_{16} = k_{\text{off,Golf}}[G_{\text{olf}}{:}\,AC{:}\,Ca^{2+}\text{-}CaM_i], \tag{S32}$$

$$V_{17} = k_{\text{on,CaM,i}}[AC][Ca^{2+}\text{-}CaM_i], \tag{S33}$$

$$V_{18} = k_{\text{off,CaM,i}}[AC{:}\,Ca^{2+}\text{-}CaM_i], \tag{S34}$$

$$V_{19} = k_{\text{on,CaM,i}}[G_{\text{olf}}{:}\,AC][Ca^{2+}\text{-}CaM_i], \tag{S35}$$

$$V_{20} = k_{\text{off,CaM,i}}[G_{\text{olf}}{:}\,AC{:}\,Ca^{2+}\text{-}CaM_i]. \tag{S36}$$

Then, the concentration of active-state AC, $[AC_{\text{active}}]$, was formulated as:

$$\frac{[AC_{\text{active}}]}{[AC]_{\text{tot}}} = \begin{cases} \sum_{i=1}^{9} \frac{[G_{\text{olf}}{:}\,AC{:}\,Ca^{2+}\text{-}CaM_i]}{[AC]_{\text{tot}}} & \text{(AC1)} \\ \frac{[G_{\text{olf}}{:}\,AC]}{[AC]_{\text{tot}}} & \text{(AC5)} \end{cases}. \tag{S37}$$

We picked up the shared part of AC1 and AC5, as a ready-for-active state of AC, AC_{primed}, as:

$$AC_{\text{primed}} = \frac{[G_{\text{olf}}{:}\,AC]}{[AC]_{\text{tot}}} \cdot \frac{K_{\text{d,Golf}} + [G_{\text{olf}}]_{\text{buff}}}{[G_{\text{olf}}]_{\text{buff}}}. \tag{S38}$$

Simplification for analytical treatment

To enable their analytical treatments, we introduced the following four constraints to the D2 model:

(a) $K_{\text{m,exch,Gi}} << [G_i{\cdot}G_{\beta\gamma}]$,

(b) $K_{\text{m,hyd,Gi}} >> [G_i\text{-}GTP]$,

(c) $[D2R]{\cdot}[DA] / [D2R{:}DA] = K_{\text{d,DA}}$, where $K_{\text{d,DA}} = k_{\text{off,DA}}/k_{\text{on,DA}}$,

(d) $V_7 = 0$.

Based on the constraints (a, b), Eqs (S5), (S6), and (S14) were simplified as:

$$V_1 \sim k_{\mathrm{cat,exch,Gi}}[D2R:DA]\,, \tag{S39}$$

$$V_2 \sim k_{RGS}[G_{\mathrm{i}}\text{-}GTP]\,, \tag{S40}$$

$$V_8 \sim k_{RGS}[AC:G_{\mathrm{i}}\text{-}GTP]\,, \tag{S41}$$

$$\text{where } k_{RGS} = k_{\mathrm{cat,hyd,Gi}}/K_{\mathrm{m,hyd,Gi}}\cdot[RGS]_{\mathrm{tot}}\,. \tag{S42}$$

Here, Eq (S39) corresponds to the catalyst-saturated approximation of the MM formulation, and Eqs (S40) and (S41) correspond to its first-order rate approximations [4]. Also, under the constraint (c) and Eq (5) and (S1), Eq (S5) was rewritten as,

$$V_1 = \begin{cases} k_{\mathrm{DA,basal}}\,[D2R]_{\mathrm{tot}} & (t < 0) \\ k_{\mathrm{DA,dip}}\,[D2R]_{\mathrm{tot}} & (t \geq 0) \end{cases}, \tag{S43}$$

$$\begin{aligned} k_{\mathrm{DA,basal}} &= \frac{k_{\mathrm{cat,exch}}[DA]_{\mathrm{basal}}}{[DA]_{\mathrm{basal}} + k_{\mathrm{off,DA}}/k_{\mathrm{on,DA}}}\,, \\ k_{\mathrm{DA,dip}} &= \frac{k_{\mathrm{cat,exch}}[DA]_{\mathrm{dip}}}{[DA]_{\mathrm{dip}} + k_{\mathrm{off,DA}}/k_{\mathrm{on,DA}}}\,. \end{aligned} \tag{S44}$$

To obtain $T_{1/2}$, we further introduced two additional constraints to the D2 model:

(e) $[AC_{\mathrm{primed}}]\cdot[G_i\text{-}GTP]/[AC{:}G_i\text{-}GTP] = K_{\mathrm{d,GiGTP}}$,

(f) $[AC_{\mathrm{primed}}]\cdot[G_i\text{-}GDP]/[AC{:}G_i\text{-}GDP] = K_{\mathrm{d,GiGDP}}$,

(g) $[AC]\cdot[G_{\mathrm{olf}}\text{-}GTP]/[AC{:}G_{\mathrm{olf}}] = K_{\mathrm{d,Golf}}$ (only for the competitive model),

where $K_{\mathrm{d,GiGTP}} = k_{\mathrm{off,GiGTP}}/k_{\mathrm{on,GiGTP}}$,, $K_{\mathrm{d,GiGDP}} = k_{\mathrm{off,GiGDP}}/k_{\mathrm{on,GiGDP}}$, and $K_{\mathrm{d,Golf}} = k_{\mathrm{off,Golf}}/k_{\mathrm{on,Golf}}$. Here, the constraints (e, f) correspond to the rapid equilibrium assumption of G_{i}, and the constraints (g) corresponds to the rapid equilibrium assumption of G_{olf}.

B. Derivations of analytical AC_{basal} and AC_{dip}

We obtained the steady-state AC_{primed} under $[DA] = [DA]_{\mathrm{basal}}$, AC_{basal}, for the standard non-competitive model (case 1) and competitive model (case 2). AC_{dip} was also obtained by replacing $[DA]_{\mathrm{basal}}$ with $[DA]_{\mathrm{dip}}$.

Case 1: Standard non-competitive model

From Eq (S24), AC_{basal} can be described as:

$$AC_{\mathrm{basal}} = \frac{[AC_{\mathrm{i}}^{\mathrm{site}}]_{\mathrm{basal}}}{[AC]_{\mathrm{tot}}} \cdot \frac{[AC_{\mathrm{olf}}^{\mathrm{site}}: G_{\mathrm{olf}}]_{\mathrm{basal}}}{[AC]_{\mathrm{tot}}} \cdot \frac{1 + G_{\mathrm{olf}}}{G_{\mathrm{olf}}}, \tag{S45}$$

where $G_{\mathrm{olf}} = [G_{\mathrm{olf}}]_{\mathrm{buff}} / K_{\mathrm{d,Golf}}$. First, considering Eq (S18) and $K_{\mathrm{d,Golf}} = [AC_{\mathrm{olf}}^{\mathrm{site}}]_{\mathrm{basal}}[G_{\mathrm{olf}}]_{\mathrm{buff}} / [AC_{\mathrm{olf}}^{\mathrm{site}}{:}G_{\mathrm{olf}}]_{\mathrm{basal}}$,

$$\frac{[AC_{\mathrm{olf}}^{\mathrm{site}}: G_{\mathrm{olf}}]_{\mathrm{basal}}}{[AC]_{\mathrm{tot}}} = \frac{G_{\mathrm{olf}}}{1 + G_{\mathrm{olf}}}\ . \tag{S46}$$

Thus, the second and third components of Eq (S45) cancel each other out, and simply,

$$AC_{\mathrm{basal}} = \frac{[AC_{\mathrm{i}}^{\mathrm{site}}]_{\mathrm{basal}}}{[AC]_{\mathrm{tot}}}. \tag{S47}$$

Then, target equations were picked up from Eqs (S3) and (S12) together with the constraint (d) as follows:

$$\frac{d}{dt}\begin{pmatrix} [G_{\mathrm{i}}\text{-}GTP] \\ [AC_{\mathrm{i}}^{\mathrm{site}}: G_{\mathrm{i}}\text{-}GTP] \\ [AC_{\mathrm{i}}^{\mathrm{site}}: G_{\mathrm{i}}\text{-}GDP] \end{pmatrix} = \begin{pmatrix} V_1 - V_2 - V_4 + V_5 \\ V_4 - V_5 - V_8 \\ -V_6 + V_8 \end{pmatrix}. \tag{S48}$$

We solved Eq (S48) under $d/dt = 0$ and $[DA] = [DA]_{\mathrm{basal}}$. Using Eqs (S8), (S9), (S39), (S40), (S43), and (S44), the first line of Eq (S48) was:

$$\begin{aligned}
&(V_1 - V_2 - V_4 + V_5)/[AC]_{\mathrm{tot}} \\
&= k_{\mathrm{DA,basal}} \frac{[D2R]_{\mathrm{tot}}}{[AC]_{\mathrm{tot}}} - \left\{ \frac{k_{RGS}}{[AC]_{\mathrm{tot}}} + k_{\mathrm{on,GiGTP}} \frac{[AC_{\mathrm{i}}^{\mathrm{site}}]_{\mathrm{basal}}}{[AC]_{\mathrm{tot}}} \right\} [G_i\text{-}GTP] \\
&\qquad + k_{\mathrm{off,GiGTP}} \frac{[AC_{\mathrm{i}}^{\mathrm{site}}: G_{\mathrm{i}}\text{-}GTP]_{\mathrm{basal}}}{[AC]_{\mathrm{tot}}}
\end{aligned}$$

$$= k_{\mathrm{DA,basal}} \frac{[D2R]_{\mathrm{tot}}}{[AC]_{\mathrm{tot}}} - \left\{ \frac{k_{RGS}}{[AC]_{\mathrm{tot}}} + k_{\mathrm{on,GiGTP}} AC_{\mathrm{basal}} \right\} [G_{\mathrm{i}}\text{-}GTP]_{\mathrm{basal}}$$

$$+ k_{\mathrm{off,GiGTP}} \frac{\left[AC_{\mathrm{i}}^{\mathrm{site}}: G_{\mathrm{i}}\text{-}GTP\right]_{\mathrm{basal}}}{[AC]_{\mathrm{tot}}}$$

$$= 0\,. \tag{S49}$$

Using Eqs (S8), (S9), and (S41), the second line of Eq (S48) was:

$$(V_4 - V_5 - V_8)/[AC]_{\mathrm{tot}}$$

$$= k_{\mathrm{on,GiGTP}} \frac{\left[AC_{\mathrm{i}}^{\mathrm{site}}\right]}{[AC]_{\mathrm{tot}}} [G_{\mathrm{i}}\text{-}GTP]_{\mathrm{basal}} - \left\{k_{\mathrm{off,GiGTP}} + k_{RGS}\right\} \frac{\left[AC_{\mathrm{i}}^{\mathrm{site}}: G_{\mathrm{i}}\text{-}GTP\right]_{\mathrm{basal}}}{[AC]_{\mathrm{tot}}}$$

$$= k_{\mathrm{on,GiGTP}} AC_{\mathrm{basal}} [G_{\mathrm{i}}\text{-}GTP]_{\mathrm{basal}} - \left\{k_{\mathrm{off,GiGTP}} + k_{RGS}\right\} \frac{\left[AC_{\mathrm{i}}^{\mathrm{site}}: G_{\mathrm{i}}\text{-}GTP\right]_{\mathrm{basal}}}{[AC]_{\mathrm{tot}}}$$

$$= 0\,. \tag{S50}$$

Using Eqs (S10) and (S41), the third line of Eq (S48) was:

$$(-V_6 + V_8)/[AC]_{\mathrm{tot}}$$

$$= -k_{\mathrm{off,GiGDP}} \frac{\left[AC_{\mathrm{i}}^{\mathrm{site}}: G_{\mathrm{i}}\text{-}GDP\right]_{\mathrm{basal}}}{[AC]_{\mathrm{tot}}} + k_{RGS} \frac{\left[AC_{\mathrm{i}}^{\mathrm{site}}: G_{\mathrm{i}}\text{-}GTP\right]_{\mathrm{basal}}}{[AC]_{\mathrm{tot}}}$$

$$= -k_{\mathrm{off,GiGDP}} \left[\left\{ \frac{\left[AC_{\mathrm{i}}^{\mathrm{site}}: G_{\mathrm{i}}\text{-}GDP\right]_{\mathrm{basal}} + \left[AC_{\mathrm{i}}^{\mathrm{site}}: G_{\mathrm{i}}\text{-}GTP\right]_{\mathrm{basal}}}{[AC]_{\mathrm{tot}}} \right\} - \frac{\left\{k_{\mathrm{off,GiGDP}} + k_{RGS}\right\}}{k_{\mathrm{off,GiGDP}}} \frac{\left[AC_{\mathrm{i}}^{\mathrm{site}}: G_{\mathrm{i}}\text{-}GTP\right]_{\mathrm{basal}}}{[AC]_{\mathrm{tot}}} \right]$$

$$= -k_{\mathrm{off,GiGDP}} \left[1 - AC_{\mathrm{basal}} - \frac{\left\{k_{\mathrm{off,GiGDP}} + k_{RGS}\right\}}{k_{\mathrm{off,GiGDP}}} \frac{\left[AC_{\mathrm{i}}^{\mathrm{site}}: G_{\mathrm{i}}\text{-}GTP\right]_{\mathrm{basal}}}{[AC]_{\mathrm{tot}}} \right]$$

$$= 0\,. \tag{S51}$$

Then, $[G_{\mathrm{i}}\text{-}GTP]_{\mathrm{basal}}$ and $[AC_{\mathrm{i}}^{\mathrm{site}}:G_{\mathrm{i}}\text{-}GTP]_{\mathrm{basal}}/[AC]_{\mathrm{tot}}$ in Eqs (S50) and (S51) were substituted into (S49),

$$-\frac{\left(k_{\mathrm{off,GiGTP}} + k_{RGS}\right)}{k_{\mathrm{on,GiGTP}}[AC]_{\mathrm{tot}}} + \left\{ \frac{\left(k_{\mathrm{off,GiGTP}} + k_{RGS}\right)}{k_{\mathrm{on,GiGTP}}[AC]_{\mathrm{tot}}} + \left(\frac{1}{k_{RGS}} + \frac{1}{k_{\mathrm{off,GiGDP}}} \right) \frac{[D2R]_{\mathrm{tot}}}{[AC]_{\mathrm{tot}}} k_{\mathrm{DA,basal}} - 1 \right\} AC_{\mathrm{basal}} + {AC_{\mathrm{basal}}}^2 = 0 \tag{S52}$$

Finally,

$$AC_{\mathrm{basal}} = \frac{-b_1 + \sqrt{b_1^2 - 4c_1}}{2}, \tag{$S53$}$$

$$b_1 = \left\{ \frac{\left(k_{\text{off,GiGTP}} + k_{RGS}\right)}{k_{\text{on,GiGTP}}[AC]_{\text{tot}}} + \left(\frac{1}{k_{RGS}} + \frac{1}{k_{\text{off,GiGDP}}} \right) \frac{[D2R]_{\text{tot}}}{[AC]_{\text{tot}}} k_{\text{DA,basal}} - 1 \right\},$$

$$c_2 = -\frac{\left(k_{\text{off,GiGTP}} + k_{RGS}\right)}{k_{\text{on,GiGTP}}[AC]_{\text{tot}}},$$

where $k_{RGS} = k_{\text{cat,hyd,Gi}}/K_{\text{m,hyd,Gi}} \cdot [RGS]_{\text{tot}}$.

Case 2: Competitive model

From Eq (S38), AC_{basal} can be described as:

$$AC_{\text{basal}} = \frac{[G_{\text{olf}}\!:AC]_{\text{basal}}}{[AC]_{\text{tot}}} \cdot \frac{1 + G_{\text{olf}}}{G_{\text{olf}}}, \tag{S54}$$

Together with the dissociation constant $K_{\text{d,Golf}} = [AC]_{\text{basal}}[G_{\text{olf}}]_{\text{buff}}/[G_{\text{olf}}\!:AC]_{\text{basal}}$, Eq (S54) was transformed to:

$$\frac{[AC]_{\text{basal}}}{[AC]_{\text{tot}}} = \frac{AC_{\text{basal}}}{1 + G_{\text{olf}}}\,. \tag{S55}$$

Also, considering Eq (S26) under no presence of Ca^{2+}-CaM,

$$\frac{[AC\!:G_{\text{i}}\text{-}GDP]_{\text{basal}} + [AC\!:G_{\text{i}}\text{-}GTP]_{\text{basal}}}{[AC]_{\text{tot}}} = 1 - AC_{\text{basal}}\,. \tag{S56}$$

Using Eqs (S55) and (S56), the first line of Eq (S48) was:

$$\begin{aligned}
&(V_1 - V_2 - V_4 + V_5)/[AC]_{\text{tot}} \\
&= k_{\text{DA,basal}} \frac{[D2R]_{\text{tot}}}{[AC]_{\text{tot}}} - \left\{ \frac{k_{RGS}}{[AC]_{\text{tot}}} + k_{\text{on,GiGTP}} \frac{[AC]_{\text{basal}}}{[AC]_{\text{tot}}} \right\} [G_{\text{i}}\text{-}GTP] + k_{\text{off,GiGTP}} \frac{[AC\!:G_{\text{i}}\text{-}GTP]_{\text{basal}}}{[AC]_{\text{tot}}} \\
&= k_{\text{DA,basal}} \frac{[D2R]_{\text{tot}}}{[AC]_{\text{tot}}} - \left\{ \frac{k_{RGS}}{[AC]_{\text{tot}}} \frac{1}{k_{\text{on,GiGTP}}} \frac{1 + G_{\text{olf}}}{AC_{\text{basal}}} + 1 \right\} k_{\text{on,GiGTP}} \frac{AC_{\text{basal}}}{1 + G_{\text{olf}}} [G_{\text{i}}\text{-}GTP] \\
&\qquad\qquad + k_{\text{off,GiGTP}} \frac{[AC\!:G_{\text{i}}\text{-}GTP]}{[AC]_{\text{tot}}} \\
&= 0\,.
\end{aligned} \tag{S57}$$

The second line of Eq (S48) was:

$$\begin{aligned}
&(V_4 - V_5 - V_8)/[AC]_{\text{tot}} \\
&= k_{\text{on,GiGTP}} \frac{[AC]_{basal}}{[AC]_{\text{tot}}} [G_i\text{-}GTP] - \left\{ k_{\text{off,GiGTP}} + k_{RGS} \right\} \frac{[AC\!:G_{\text{i}}\text{-}GTP]_{\text{basal}}}{[AC]_{\text{tot}}}
\end{aligned}$$

$$= k_{\text{on,GiGTP}} \frac{AC_{\text{basal}}}{1+G_{\text{olf}}} [G_i\text{-}GTP] - \{k_{\text{off,GiGTP}} + k_{RGS}\} \frac{[AC{:}\,G_{\text{i}}\text{-}GTP]_{\text{basal}}}{[AC]_{\text{tot}}}$$

$$=0\,. \tag{S58}$$

The third line was:

$$(-V_6 + V_8)/[AC]_{\text{tot}}$$

$$= -k_{\text{off,GiGDP}} \frac{[AC{:}\,G_{\text{i}}\text{-}GDP]_{\text{basal}}}{[AC]_{\text{tot}}} + k_{RGS} \frac{[AC{:}\,G_{\text{i}}\text{-}GTP]_{\text{basal}}}{[AC]_{\text{tot}}}$$

$$= -k_{\text{off,GiGDP}} \left[1 - AC_{\text{basal}} - \frac{\{k_{\text{off,GiGDP}} + k_{RGS}\}}{k_{\text{off,GiGDP}}} \frac{[AC{:}\,G_{\text{i}}\text{-}GTP]_{\text{basal}}}{[AC]_{\text{tot}}}\right]$$

$$= 0\,. \tag{S59}$$

Then, $[G_{\text{i}}\text{-}GTP]_{\text{basal}}$ and $[AC{:}G_{\text{i}}\text{-}GTP]_{\text{basal}}/[AC]_{\text{tot}}$ in Eqs (S58) and (S59) were substituted into (S57),

$$0 = -\alpha + \left[\alpha + k_{\text{DA,basal}} \left(\frac{1}{k_{RGS}} + \frac{1}{k_{\text{off,GiGDP}}}\right) \frac{[D2R]_{\text{tot}}}{[AC]_{\text{tot}}} - 1\right] AC_{\text{basal}} + {AC_{\text{basal}}}^2\,, \tag{$S60$}$$

$$\alpha = \frac{\{k_{\text{off,GiGTP}} + k_{RGS}\}}{k_{\text{on,GiGTP}}[AC]_{\text{tot}}} (1 + G_{\text{olf}})\,.$$

Finally,

$$AC_{\text{basal}} = \frac{-b_2 + \sqrt{b_2^2 - 4c_2}}{2}, \tag{$S61$}$$

$$b_1 = \left\{\frac{(k_{\text{off,GiGTP}} + k_{RGS})}{k_{\text{on,GiGTP}}} \frac{(1+G_{\text{olf}})}{[AC]_{\text{tot}}} + \left(\frac{1}{k_{RGS}} + \frac{1}{k_{\text{off,GiGDP}}}\right) \frac{[D2R]_{\text{tot}}}{[AC]_{\text{tot}}} k_{\text{DA,basal}} - 1\right\},$$

$$c_2 = -\frac{(k_{\text{off,GiGTP}} + k_{RGS})}{k_{\text{on,GiGTP}}} \frac{(1+G_{\text{olf}})}{[AC]_{\text{tot}}}\,.$$

C. Derivation of analytical $T_{1/2}$

In the standard non-competitive model, we first obtained $\widehat{AC}_{\text{primed}}$ that denotes AC_{primed} under the constraints (e, f, g). The constraints (d, f) ($V_7 = 0$, or $k_{\text{on,GiGDP}} = 0$) gave $[AC{:}G_{\text{i}}\text{-}GDP] = 0$ for any t; thus, $\widehat{AC}_{\text{primed}}$ was derived based on Eqs (S13) and (S47) with the assumption of constant $[G_{\text{olf}}]_{\text{buff}}$:

$$\widehat{AC}_{\text{primed}} = \frac{[AC_{\text{i}}^{\text{site}}]}{[AC]_{\text{tot}}} = 1 - \frac{[AC_{\text{i}}^{\text{site}}{:}\,G_i\text{-}GTP]}{[AC]_{\text{tot}}}. \qquad (S62)$$

Eqs (S13) and (S62), and $[AC{:}G_{\text{i}}\text{-}GDP] = 0$ were then substituted into the constraint (e):

$$\widehat{AC}_{\text{primed}}{}^{2} + \left\{\frac{[GTP] + K_{\text{d,GiGTP}}}{[AC]_{\text{tot}}} - 1\right\}\widehat{AC}_{\text{primed}} - \frac{K_{\text{d,GiGTP}}}{[AC]_{\text{tot}}} = 0 \ , \qquad (S63)$$

or

$$[GTP] = \left\{\frac{K_{\text{d,GiGTP}}}{\widehat{AC}_{\text{primed}}} + [AC]_{\text{tot}}\right\}(1 - \widehat{AC}_{\text{primed}}). \qquad (S64)$$

Eq. (S63) is a simplified form of Eq. (S52) under $k_{\text{off,GiGDP}} \gg k_{RGS}$ and $k_{\text{off,GiGTP}} \gg k_{RGS}$. Then, the dynamics of $[GTP] = [G_{\text{i}}\text{-}GTP] + [AC_{\text{i}}^{\text{site}}{:}G_{\text{i}}\text{-}GTP]$ was derived using Eqs (S3) and (S12), under Eqs (S40) –(S42) (constraint (b)):

$$\begin{aligned} \frac{d}{dt}[GTP] &= \frac{d}{dt}[G_{\text{i}}\text{-}GTP] + \frac{d}{dt}[AC_{\text{i}}^{\text{site}}{:}\,G_{\text{i}}\text{-}GTP] \\ &= (V_1 - V_2 - V_4 + V_5) + (V_4 - V_5 - V_8) \\ &= V_1 - (V_2 + V_8) \\ &= V_1 - k_{RGS}[GTP]. \end{aligned} \qquad (S65)$$

On Eqs (S43) and (S44):

$$[GTP] = \begin{cases} \dfrac{[D2R]_{\text{tot}}}{k_{RGS}} k_{\text{DA,basal}} & (t \le 0) \\ \dfrac{[D2R]_{\text{tot}}}{k_{RGS}} \{(k_{\text{DA,basal}} - k_{\text{DA,dip}})\exp(-k_{RGS}t) + k_{\text{DA,dip}}\} & (t > 0) \end{cases} . \qquad (S66)$$

Finally, substituting Eq (S66) into Eq (S64), the half maximal time constant, $\hat{T}_{1/2}$, under the standard non-competitive model was obtained as follows:

$$\hat{T}_{1/2} = \frac{1}{k_{RGS}} \ln \left\{ \frac{k_{\mathrm{DA,basal}} - k_{\mathrm{DA,dip}}}{\left(1 - \widehat{AC}_{1/2}\right)\left\{[AC]_{\mathrm{tot}} + \frac{K_{\mathrm{d,GiGTP}}}{\widehat{AC}_{1/2}}\right\}\frac{k_{RGS}}{[D2R]_{\mathrm{tot}}} - k_{\mathrm{DA,dip}}} \right\}, \quad (S67)$$

where $\widehat{AC}_{1/2} = (\widehat{AC}_{\mathrm{basal}} + \widehat{AC}_{\mathrm{dip}})/2$ was given based on Eq (S52) and (S63) as follows:

$$\widehat{AC}_{\mathrm{basal}} = \frac{-b_3 + \sqrt{b_3^2 - 4c_3}}{2}, \quad (S68)$$

$$b_3 = \frac{K_{\mathrm{d,GiGTP}}}{[AC]_{\mathrm{tot}}} + \frac{[D2R]_{\mathrm{tot}}}{[AC]_{\mathrm{tot}}}\frac{k_{\mathrm{DA,basal}}}{k_{RGS}} - 1,$$

$$c_3 = -\frac{K_{\mathrm{d,GiGTP}}}{[AC]_{\mathrm{tot}}},$$

$$\widehat{AC}_{\mathrm{dip}} = \frac{-b_4 + \sqrt{b_4^2 - 4c_4}}{2}, \quad (S69)$$

$$b_4 = \frac{K_{\mathrm{d,GiGTP}}}{[AC]_{\mathrm{tot}}} + \frac{[D2R]_{\mathrm{tot}}}{[AC]_{\mathrm{tot}}}\frac{k_{\mathrm{DA,dip}}}{k_{RGS}} - 1,$$

$$c_4 = -\frac{K_{\mathrm{d,GiGTP}}}{[AC]_{\mathrm{tot}}}.$$

We also obtained $\hat{T}_{1/2}$ in the competitive model under the rapid equilibrium assumption of G_{olf} and G_i, i.e., the constraints (g) and (e, f), respectively. The constraint (g) gave $\widehat{AC}_{\mathrm{primed}} = (1 + G_{\mathrm{olf}})\,[AC]/[AC]_{\mathrm{tot}}$ (see Eq (S55)), and the constraint (d, f) gave [*AC*:*G*i-*GDP*] = 0. They simplified Eq (S28) (identical to Eq (S26) under no Ca^{2+}-CaM) into:

$$[GTP] = \left\{\frac{K_{\mathrm{d,GiGTP}}}{\widehat{AC}_{\mathrm{primed}}}(1 + G_{\mathrm{olf}}) + [AC]_{\mathrm{tot}}\right\}\left(1 - \widehat{AC}_{\mathrm{primed}}\right). \quad (S70)$$

Substituting Eq (S70) into Eq (S66), the half maximal time constant, $\hat{T}_{1/2}$, was obtained as follows:

$$\hat{T}_{1/2} = \frac{1}{k_{RGS}} \ln \left\{ \frac{k_{\mathrm{DA,basal}} - k_{\mathrm{DA,dip}}}{\left(1 - \widehat{AC}_{1/2}\right)\left\{[AC]_{\mathrm{tot}} + K_{\mathrm{d,GiGTP}}\frac{1 + G_{\mathrm{olf}}}{\widehat{AC}_{1/2}}\right\}\frac{k_{RGS}}{[D2R]_{\mathrm{tot}}} - k_{\mathrm{DA,dip}}} \right\}, \quad (S71)$$

where $\widehat{AC}_{1/2} = (\widehat{AC}_{\mathrm{basal}} + \widehat{AC}_{\mathrm{dip}})/2$ was given by:

$$\widehat{AC}_{\mathrm{basal}} = \frac{-b_5 + \sqrt{b_5^2 - 4c_5}}{2}, \quad (S72)$$

$$b_5 = \frac{K_{\mathrm{d,GiGTP}}}{[AC]_{\mathrm{tot}}}(1 + G_{\mathrm{olf}}) + \frac{[D2R]_{\mathrm{tot}}}{[AC]_{\mathrm{tot}}}\frac{k_{\mathrm{DA,basal}}}{k_{RGS}} - 1 \,,$$

$$c_5 = -\frac{K_{\mathrm{d,GiGTP}}}{[AC]_{\mathrm{tot}}}\,(1 + G_{\mathrm{olf}}) \,,$$

$$\widehat{AC}_{\mathrm{dip}} = \frac{-b_6 + \sqrt{b_6^2 - 4c_6}}{2}, \qquad (S73)$$

$$b_6 = \frac{K_{\mathrm{d,GiGTP}}}{[AC]_{\mathrm{tot}}}(1 + G_{\mathrm{olf}}) + \frac{[D2R]_{\mathrm{tot}}}{[AC]_{\mathrm{tot}}}\frac{k_{\mathrm{DA,dip}}}{k_{RGS}} - 1 \,,$$

$$c_6 = -\frac{K_{\mathrm{d,GiGTP}}}{[AC]_{\mathrm{tot}}}\,(1 + G_{\mathrm{olf}}) \,.$$

References

1. Iino Y, Sawada T, Yamaguchi K, Tajiri M, Ishii S, Kasai H, et al. Dopamine D2 receptors in discrimination learning and spine enlargement. Nature. 2020; 579(7800): 555-560. Epub 2020/03/28. pmid: 32214250.
2. Hollinger S, Hepler JR. Cellular regulation of RGS proteins: modulators and integrators of G protein signaling. Pharmacol Rev. 2002; 54(3): 527-559. Epub 2002/09/12. pmid: 12223533.
3. Urakubo H, Yagishita S, Kasai H, Ishii S. Signaling models for dopamine-dependent temporal contiguity in striatal synaptic plasticity. PLoS Comput Biol. 2020; 16(7): e1008078. Epub 2020/07/24. pmid: 32701987.
4. Katanaev VL, Chornomorets M. Kinetic diversity in G-protein-coupled receptor signalling. Biochem J. 2007; 401(2): 485-495. pmid: 16989639.
